# Targeted proximity-labelling of protein tyrosines *via* flavin-dependent photoredox catalysis with mechanistic evidence for a radical–radical recombination pathway†

**DOI:** 10.1039/d3sc00638g

**Published:** 2023-05-17

**Authors:** Taylor O. Hope, Tamara Reyes-Robles, Keun Ah Ryu, Steven Mauries, Nicole Removski, Jacinthe Maisonneuve, Rob C. Oslund, Olugbeminiyi O. Fadeyi, Mathieu Frenette

**Affiliations:** a Department of Chemistry, NanoQAM, Centre Québécois des Matériaux Fonctionnels (CQMF), Université du Québec à Montréal Montréal Québec H3C 3P8 Canada frenette.mathieu@uqam.ca; b Exploratory Science Center, Merck & Co., Inc. Cambridge MA USA niyi@induprolabs.com rob@induprolabs.com

## Abstract

Flavin-based photocatalysts such as riboflavin tetraacetate (RFT) serve as a robust platform for light-mediated protein labelling *via* phenoxy radical-mediated tyrosine–biotin phenol coupling on live cells. To gain insight into this coupling reaction, we conducted detailed mechanistic analysis for RFT-photomediated activation of phenols for tyrosine labelling. Contrary to previously proposed mechanisms, we find that the initial covalent binding step between the tag and tyrosine is not radical addition, but rather radical–radical recombination. The proposed mechanism may also explain the mecha-nism of other reported tyrosine-tagging approaches. Competitive kinetics experiments show that phenoxyl radicals are generated with several reactive intermediates in the proposed mechanism—primarily with the excited riboflavin-photocatalyst or singlet oxygen—and these multiple pathways for phenoxyl radical generation from phenols increase the likelihood of radical–radical recombination.

## Abstract 2

Labelling of protein tyrosines with phenol-containing tags is shown using riboflavin based-photocatalysts with a smaller labelling radius than peroxidase-based methods. Mechanistic insight also suggests a radical recombination mechanism.

## Graphical abstract

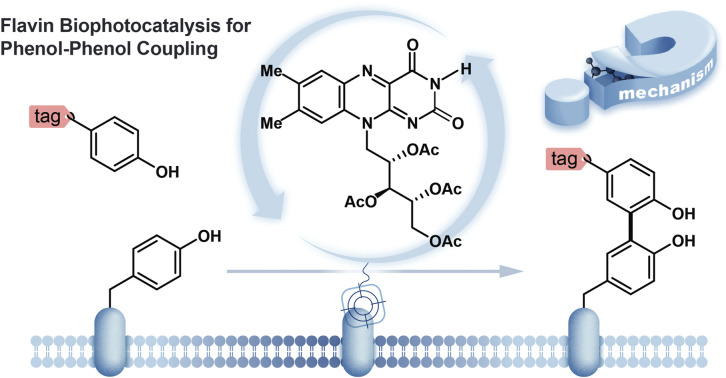

## Introduction

Technologies that identify interacting proteins are crucial to understand fundamental biological processes and to enable new drug target discoveries. Visible light photocatalysis has emerged as an attractive platform for this purpose to achieve selective chemical transformations on and within biological materials with spatiotemporal control.^1–11^ A key feature is that visible light selectively excites a photocatalyst; the excited photocatalyst then activates chemical tags for covalent protein labelling. Tag molecules are usually activated to become reactive intermediates with short lifetimes, such as radicals,^10,11^ carbenes,^8^ or nitrenes,^9^ to limit the labelling radius within a complex biological environment. Importantly, a single photocatalyst can activate multiple tags resulting in substantial signal amplification for labelling with bioorthogonal handles such as biotin, azides, alkynes, or fluorophores for downstream protein analysis.^1,12^

**Fig. 1:**
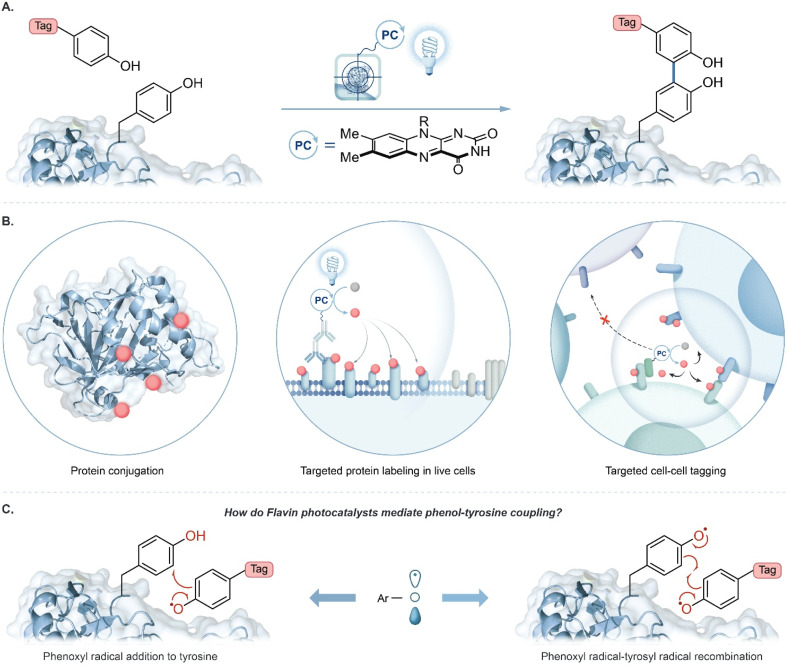
(A) Flavin-based photocatalytic tyrosine-tagging by phenol containing tags. (B) Biological applications of RFT-mediated photocatalysis in protein and cellular environments. (C) Competing mechanistic proposals for the labelling of tyrosine by phenoxyl radicals: radical addition to tyrosine *versus* radical recombination.

A major trend in proximity protein tagging is to exploit the reactivity of tyrosine at a protein's surface. Notably, peroxidase-enabled tyrosine labelling has been developed for profiling protein environments in numerous cellular contexts.^13^ In this system initiated by exogenous hydrogen peroxide, a phenol-containing tag is oxidized to phenoxyl radicals by heme-containing peroxidases resulting in the labelling of nearby proteins. Recently, we reported the use of a flavin-derived cofactor, riboflavin tetraacetate (RFT), as a photocatalyst for the generation of phenoxyl radical intermediates for tyrosine-based protein labelling (Fig. 1A).^4^ Blue-light activated RFT was shown to achieve proximity labelling of proteins, live cells, and cell–cell contact regions (Fig. 1B). Similar to other reported tyrosine tagging methods, radical addition onto a neutral tyrosine is traditionally proposed as a key step in the mechanism.^10,11,14–16^ However, to date, no direct evidence has been reported for this mechanism. Here, we examine the proposed radical addition mechanism and a competing radical–radical recombination pathway (Fig. 1C).

## Results and discussion

**Fig. 2:**
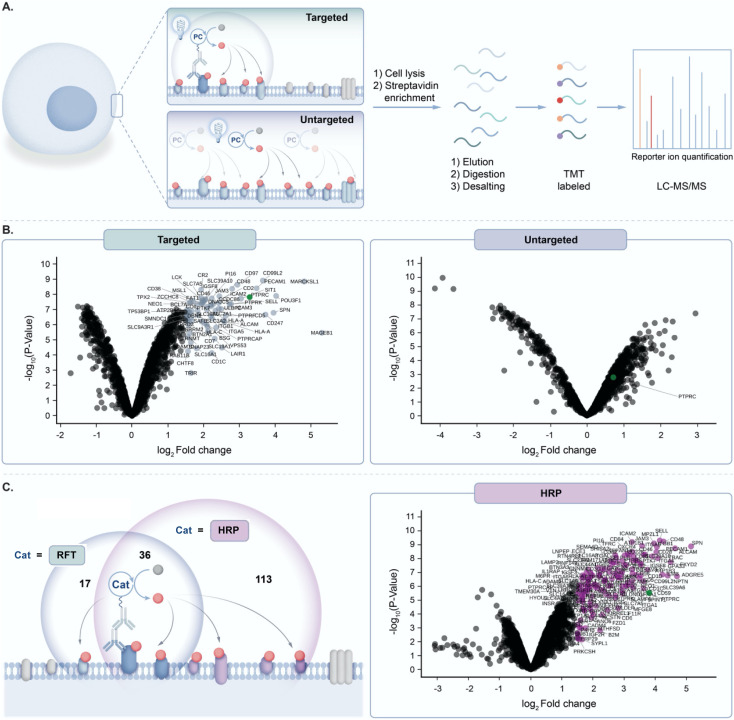
(A) Schematic depicting targeted and untargeted labelling of CD45 (PTPRC) on the surface of Jurkat cells. Cells are labeled with an anti-CD45 primary antibody/secondary antibody RFT photocatalyst conjugate (targeted) or with free RFT photocatalyst (untargeted) followed by cell lysis, protein enrichment and digestion, and LC-MS/MS-based proteomic analysis. (B) Left, volcano plot of statistical significance *vs.* fold-enrichment for CD45-targeted *vs.* isotype-targeted biotinylation on Jurkat cells using an anti-CD45 primary antibody/secondary antibody RFT conjugate with 10 minutes of visible light activation in the presence of biotin tyramide. Significantly enriched proteins (*p*-value < 0.05 and log_2_ FC > 1.5) are indicated in light blue and CD45 (PTPRC) is labeled in green (*n* = 3 experiments). Right, volcano plot of statistical significance *vs.* fold-enrichment of free RFT photocatalyst and biotin tyramide *vs.* biotin tyramide only on Jurkat cells after 10 minutes of visible light activation. CD45 (PTPRC) is labeled in green (*n* = 3 experiments). (C) Left, schematic depicting CD45 targeted labelling between RFT and peroxidase (HRP)-based methods. Circles reflect a Venn diagram of the significantly enriched proteins from targeted labelling with HRP (purple circle, (C) right panel) or RFT (light blue circle, Fig. S2†). Right, volcano plot of statistical significance *vs.* fold-enrichment for CD45-targeted *vs.* isotype-targeted biotinylation on Jurkat cells using an anti-CD45 primary antibody/secondary antibody peroxidase conjugate (HRP) or isotype/secondary antibody peroxidase conjugate with 1 minute of labelling in the presence of biotin tyramide and hydrogen peroxide. Significantly enriched proteins (*p*-value < 0.05 and log_2_ FC > 1.5) are indicated in purple and CD45 (PTPRC) is labeled in green (*n* = 3 experiments).

Prior to exploring the mechanistic basis of RFT-mediated phenol–phenol coupling, we developed a novel proteomic workflow to evaluate the effect of targeted activation of RFT in a localized microenvironment. Having previously demonstrated by flow cytometry and confocal imaging that we can achieve RFT-mediated protein labelling on live cells,^4^ this proteomic workflow focused on the evaluation of targeted *versus* untargeted activation of RFT localized to the cell surface (Fig. 2A). In these experiments, RFT was conjugated to a secondary antibody that can bind to a primary antibody recognizing cell surface proteins. We selected PTPRC (CD45), a T cell marker highly expressed on the cell surface,^17^ as our target.

Accordingly, targeted delivery of RFT to PTPRC *via* the primary/secondary antibody system, followed by irradiation with blue light in the presence of biotin tyramide led to detection of PTPRC among the highest enriched proteins (Fig. 2B). Known PTPRC interactors such as the PTPRC associated protein (PTPRCAP), CD2, and LCK were also highly co-enriched with PTPRC (Fig. 2B and S1†). In contrast, untargeted delivery of free RFT catalysts to live cells (Fig. 2A) followed by irradiation with blue light in the presence of biotin tyramide failed to enrich PTPRC (Fig. 2B). Since the peroxidase enzyme similarly leverages biotin tyramide to achieve protein labelling,^13^ we compared our RFT protein system to peroxidase-based labelling of CD45 on live cells followed by mass spectrometry based proteomic analysis. We observed a nearly three-fold increase in total protein enrichment using peroxidase-based labelling compared to RFT (Fig. 2C and S2†) suggesting a much larger effective labelling radius achieved by the enzyme-based method. Collectively these results highlight the ability of targeted activation of RFT to achieve localized phenoxyl radical tagging within complex cellular systems.

**Fig. 3:**
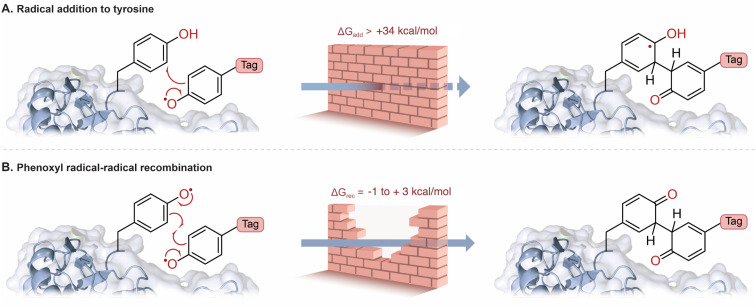
(A) The addition of a phenoxyl radical tag to tyrosine is highly unfavorable (Table S10†). (B) Radical–radical recombination of phenoxyl radicals is more favorable, and rearromatization leads to a strong covalent bond (Table S9†). The relative rate constants were estimated according to Eyring's equation (see ESI† for details).

Likewise, tyrosine radical addition onto a neutral aromatic system has been proposed (Fig. 1C).^10,14^ In both cases, prohibitively large activation energies are calculated for these proposed mechanisms (Tables S12 and S15†). A major contribution of this study is to bring convincing evidence against radical addition to tyrosine; the thermodynamic cost of this radical addition step is prohibitively unfavorable (Fig. 3A, Δ*G*_DFT_ > 34 kcal mol^−1^, Table S10†). The recombination of phenoxyl radicals, however, is much more probable (Fig. 3B, Δ*G*_DFT_ = −1 to +3 kcal mol^−1^, Table S9†). The slight endergonic recombination is a testament to phenoxyl radical stability—of course, the initial recombination product will rapidly tautomerize to regenerate aromaticity for a largely favorable end-product (Δ*G*_DFT_ = −36 kcal mol^−1^, Table S9†). Photocatalysis often employs metal-based complexes due to their long-lived excited state lifetimes and tunable redox potentials,^18–20^ however, more bio-compatible photocatalysts such as RFT are desirable in protein labelling.^21^ We explored RFT photochemistry and found several pathways by which phenoxyl radicals, from tags and tyrosine, could be generated. The multiple pathways that generate phenoxyl radicals support evidence of radical–radical recombination as a likely mechanism in these systems.

**Fig. 4:**
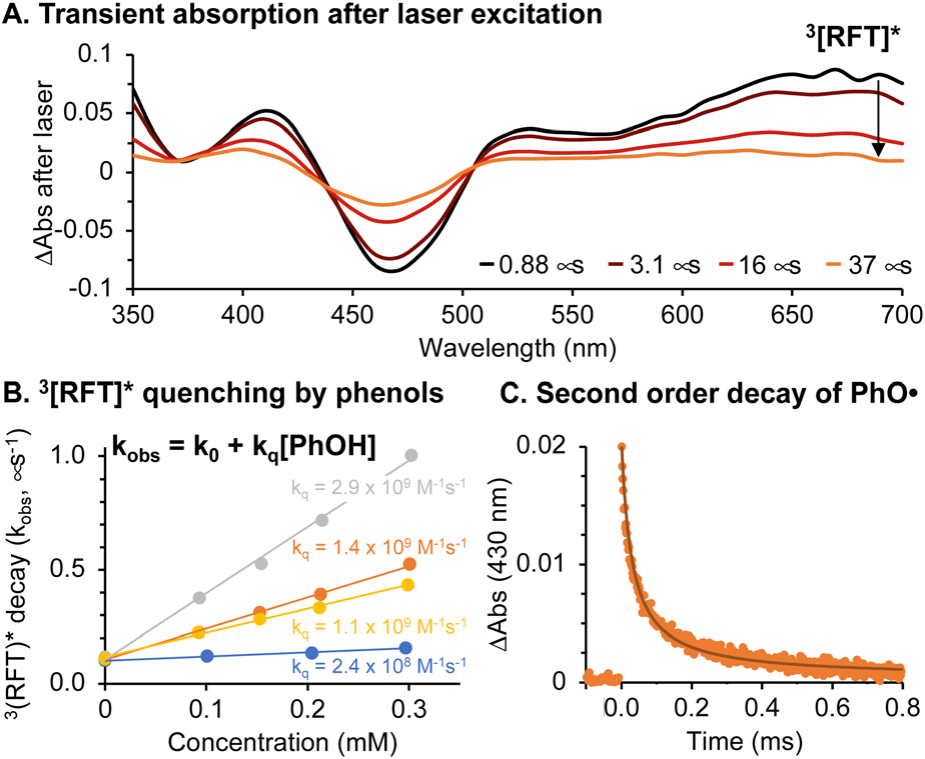
(A) Transient absorption of RFT (0.06 mM) in deoxygenated acetonitrile : water (1 : 1) following 355 nm laser excitation. (B) Stern–Volmer quenching of ^3^[RFT]* with 2,6-dimethoxyphenol (grey), biotin tyramide (orange), Ac–Tyr–NHMe (yellow) and BSA (blue). (C) Transient absorption of phenoxyl radicals from biotin tyramide and fit to second order decay kinetics (see ESI and Fig. S17† for details).

The photocatalytic cycle begins with blue light absorption by RFT followed by formation of a triplet excited state, 3[RFT]*. We observed ^3^[RFT]* to display a relatively long excited state lifetime of 12 μs in deoxygenated solution, as determined by following its characteristic absorption at 700 nm in laser-flash photolysis experiments (Fig. 4A).

Due to their oxidizing nature, flavin-based triplet excited states can initiate radical chemistry.^22^ In our system, phenol-containing tags or tyrosines in a protein will be rapidly oxidized by ^3^[RFT]* to generate phenoxyl radicals. The rate constant (*k*_q_) for this bimolecular process was determined by Stern–Volmer kinetics (Fig. 4B), which shows the oxidation of phenols with ^3^[RFT]* to be fast in measured examples (10^8^ to 10^9^ M^−1^ s^−1^) (Table S2†). When generated in laser flash photolysis experiments, phenoxyl radicals display a typical 2^nd^ order decay as shown in Fig. 4C since they undergo a radical–radical recombination as their main decay pathway.

**Fig. 5:**
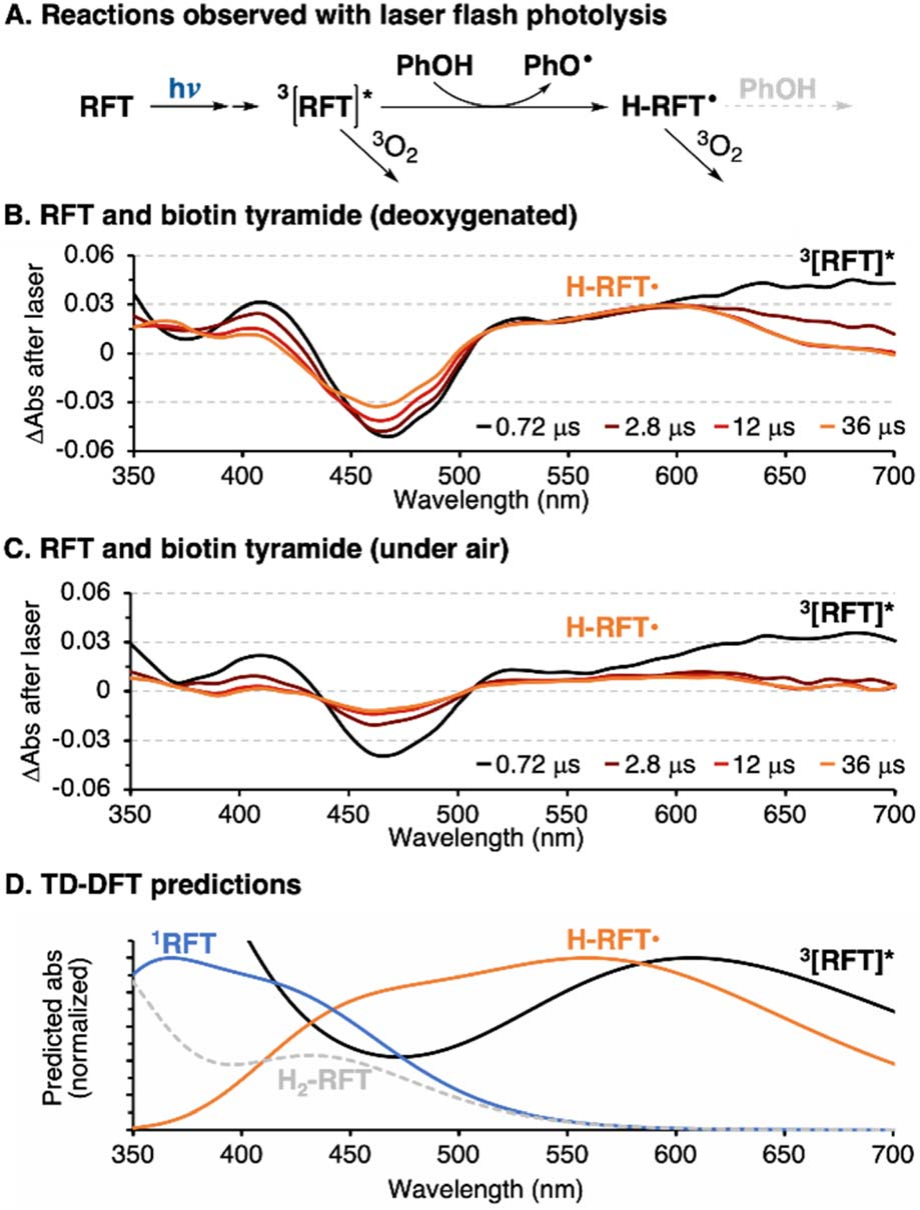
(A) Reactions observed by laser-flash photolysis. (B) Transient absorption of RFT (0.06 mM) and biotin tyramide (0.3 mM) in the absence of oxygen after a 355 nm laser pulse. (C) Transient absorption of the same RFT (0.06 mM) and biotin tyramide (0.3 mM) solution open to air. (D) TD-DFT predicted absorption spectra of ground state RFT (blue), ^3^[RFT]* (black), H-RFT˙ (orange) and H_2_-RFT (dashed grey).

In the absence of oxygen, ^3^[RFT]* will quickly be reduced by phenols, either from a tag or tyrosine group (Fig. 5A, Table S5†). The transient absorption of ^3^[RFT]* at 700 nm rapidly decays in the presence of phenols to form a relatively stable intermediate with an absorption centered at 600 nm (Fig. 5B). We identify this peak as the semi reduced form, H-RFT˙, with supporting evidence provided by TD-DFT predicted spectra (Fig. 5D). Semi-reduced flavins with similar chemical structures absorb in the same region.^22^ Both transient absorption peaks associated with ^3^[RFT]* and H-RFT˙ are strongly attenuated by the introduction of oxygen (Fig. 5C).

We applied this photocatalytic coupling method on nucleophilic phenol substrates and tyrosine containing peptides where we observed tyrosine–phenol cross-coupling in moderate yields (Fig. S3 and Table S4†). During the optimization of our synthetic method, we found oxygen to be crucially important in maximizing the conversion efficiency (Table S4†). While the synthetic and protein labelling conditions differ in phenol type and concentration, this observation led us to investigative the role oxygen plays in the overall reaction mechanism.

Flavins are well-known singlet oxygen (^1^O_2_) sensitizers^22^ and ^1^O_2_ can oxidize phenols to phenoxyl radicals.^24–27^ Using competitive kinetics with the probe 9,10-diphenylanthracene (DPA),^23^ we measured the rate constant for the reaction of ^1^O_2_ with electron rich phenols and a tyrosine analog to be ∼10^7^ M^−1^ s^−1^ (Fig. S24†). The H-abstraction from phenols by ^1^O_2_ is calculated to be favorable with a Δ*G*_DFT_ from −12.3 to −18.0 kcal mol^−1^ (Table S7†).

**Fig. 6:**
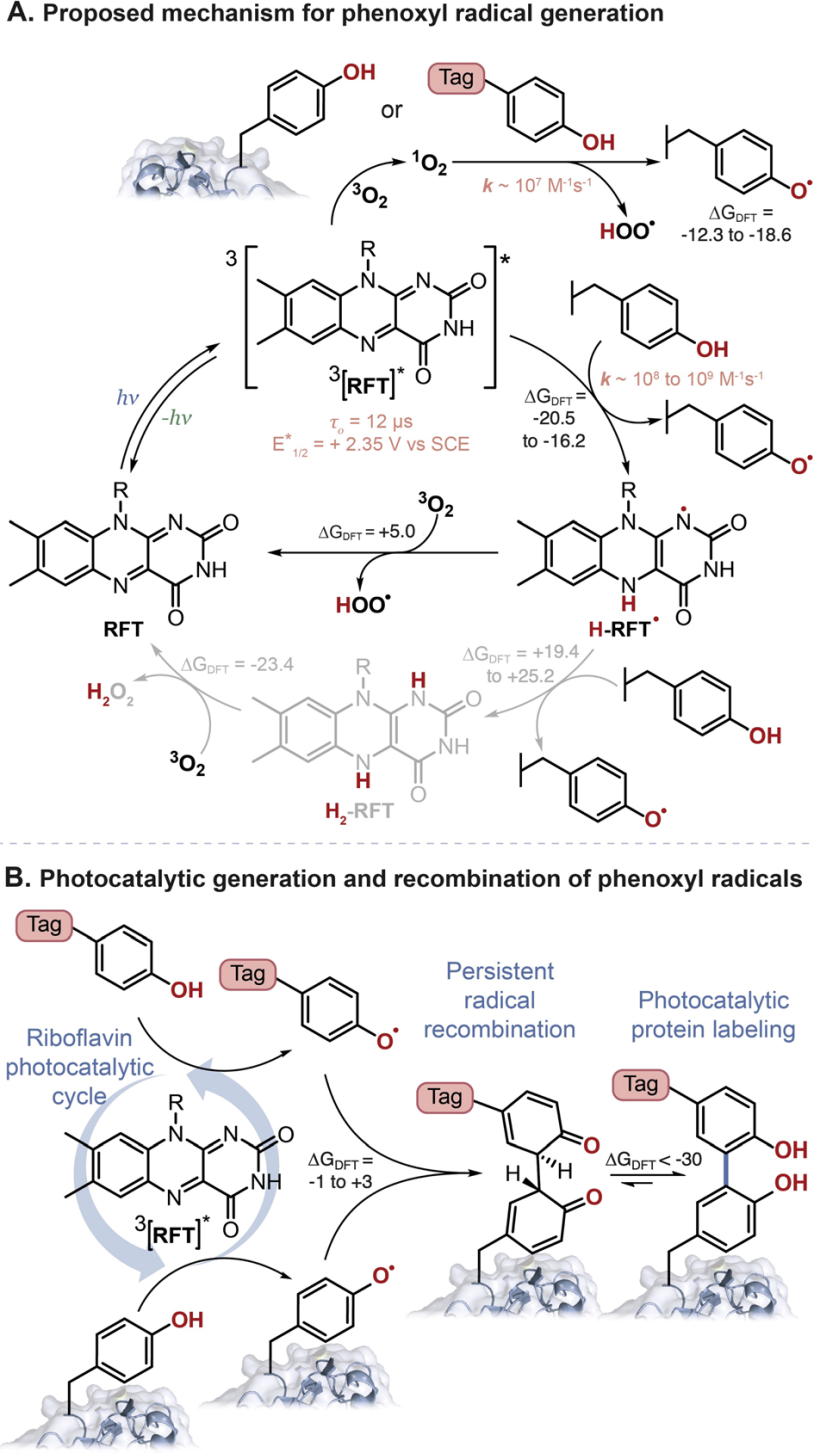
(A) Proposed photocatalytic reaction mechanism for the generation of phenoxyl radicals from tyrosine-containing protein and phenol-containing tag molecules. (B) Phenoxyl radical are persistent radicals, and as such will recombine followed by rearomatization, as the preferred mechanism for phenol–phenol coupling. Δ*G*_DFT_, shown in kcal mol^−1^, were calculated using B3LYP-D3BJ/6-311+G(2d,2p)//CPCM(H_2_O) with various phenols (see ESI† for details).

Oxygen also plays a role in the photocatalytic turnover of RFT. Following excitation, ^3^[RFT]* will react with phenols to form phenoxyl radicals and H-RFT˙. Two pathways to regenerate RFT from H-RFT˙ are considered. As often proposed in the literature for flavins, H-RFT˙ can be further reduced to H_2_-RFT,^11,22,28^ however, we calculate this reduction to be significantly unfavorable in our system. Phenoxyl radical generation from the reaction between phenols and H-RFT˙ gave Δ*G*_DFT_ values in the range of +19.4 to +25.2 kcal mol^−1^ (Table S6†). A second pathway to regenerate RFT from H-RFT˙ bypasses the need to generate H_2_-RFT. In the proposed mechanism (Fig. 6A), oxygen will directly oxidize H-RFT˙ back to RFT with the formation of HOO˙. This pathway has a more favorable Δ*G*_DFT_ of +4.99 kcal mol^−1^ than the full reduction (Δ*G*_DFT_ > +17 kcal mol^−1^).

The detection of H_2_O_2_ as a by-product has been used as evidence for the catalytic cycle to involve H_2_-RFT.^16^ Indeed, the reduction of H_2_-RFT, in a presumably multistep process, could favorably generate H_2_O_2_ in the presence of oxygen (Δ*G*_DFT_ = −23.4 kcal mol^−1^). However, H_2_O_2_ can also be generated in pathways that do not include H_2_-RFT. The reaction of ^1^O_2_ with phenols will generate HOO˙ as could the reaction of ^3^O_2_ with H-RFT˙. Both H-abstraction by HOO˙ and recombination of two HOO˙ can explain the presence of H_2_O_2._

Finally, as proposed in Fig. 6B the phenoxyl radicals will recombine and favourably tautomerize (Δ*G*_DFT_ = −1 to +3 kcal mol^−1^ and ∼−36 kcal mol^−1^, for each step respectively. See Table S9†).

Traditionally, the need for two radicals to recombine in a synthetically useful transformation is rare—the concentration of transient radicals is often too low to explain high yielding reactions. In this case, however, the persistence of phenoxyl radicals is well-documented—several antioxidants are based on phenoxyl radical's lack of side-reactions.^29^ In biological systems, phenoxyl radicals are relatively persistent due to their low reactivity with oxygen and their inability to perform H-abstraction with most biomolecules—their estimated lifetime is ∼0.1 ms in cell media.^8^

The proposed mechanism described herein helps explain the success of phenoxyl radical labelling methods. Radical–radical recombination follows bimolecular kinetics, and the efficiency of this process will dramatically decrease as the distance increases away from the photochemical source of phenoxyl radicals. Another important factor in the success of this protein tagging method is the strength of the bonds formed. Radical–radical recombination becomes more predominant with increasing radical persistence; however, the recombination of persistent radicals can lead to weak and reversible bonds as is the case for certain carbon-centered radicals.^30^ The recombination of phenoxyl radical is followed by tautomeric aromatization leading to bond strengthening. Altogether, these mechanistic insights will be an important consideration as different tag motifs are considered in photochemical protein tagging. Furthermore, the radical–radical recombination mechanism described here might be a potential pathway that occurs in the case of peroxidase-based labelling systems.^31,32^

## Conclusion

We envision that the insights gained from our mechanistic analysis will lead to improved catalytic performance of these flavin-based systems and further encourage the development of new and effective light-mediated labelling strategies in the field of chemical biology.

## Data availability

Computational data is resumed in the ESI.† No other data in data repositories.

## Author contributions

The manuscript was written by T. O. H., R. C. O., O. O. F. and M. F. All authors have given approval to the final version of the manuscript.

## Conflicts of interest

T. R. R., K. A. R., R. C. O., and O. O. F. were employed by Merck Sharp & Dohme Corp., a subsidiary of Merck & Co., Inc., Rah-way, NJ, USA during the experimental planning, execution and/or preparation of this manuscript.

## Supplementary Material

SC-014-D3SC00638G-s001

## References

[cit1] Ryu K. A., Kaszuba C. M., Bissonnette N. B., Oslund R. C., Fadeyi O. O. (2021). Interrogating biological systems using visible-light-powered catalysis. Nat. Rev. Chem..

[cit2] Müller M., Gräbnitz F., Barandun N., Shen Y., Wendt F., Steiner S. N., Severin Y., Vetterli S. U., Mondal M., Prudent J. R., Hofmann R. (2021). Light-mediated discovery of surfaceome nanoscale organization and intercellular receptor interaction networks. Nat. Commun..

[cit3] Buksh B. F., Knutson S. D., Oakley J. V., Bissonnette N. B., Oblinsky D. G., Schwoerer M. P., Seath C. P., Geri J. B., Rodriguez-Rivera F. P., Parker D. L., Scholes G. D. (2022). μMap-Red: Proximity Labelling by Red Light Photocatalysis. J. Am. Chem. Soc..

[cit4] Oslund R. C., Reyes-Robles T., White C. H., Tomlinson J. H., Crotty K. A., Bowman E. P., Chang D., Peterson V. M., Li L., Frutos S., Vila-Perello M., Vlerick D., Cromie K., Perlman D. H., Ingale S., O’Hara S. D., Roberts L. R., Piizzi G., Hett E. C., Hazuda D. J., Fadeyi O. O. (2022). Detection of Cell-Cell Interactions *via* Photocatalytic Cell Tagging. Nat. Chem. Biol..

[cit5] Wang H., Zhang Y., Zeng K., Qiang J., Cao Y., Li Y., Fang Y., Zhang Y., Chen Y. (2021). Selective Mitochondrial Protein Labelling Enabled by Biocompatible Photocatalytic Reactions inside Live Cells. JACS Au.

[cit6] Tamura T., Takato M., Shiono K., Hamachi I. (2020). Development of a photoactivatable proximity labelling method for the identification of nuclear proteins. Chem. Lett..

[cit7] Trowbridge A. D., Seath C. P., Rodriguez-Rivera F. P., Li B. X., Dul B. E., Schwaid A. G., Buksh B. F., Geri J. B., Oakley J. V., Fadeyi O. O., Oslund R. C., Ryu K. A., White C., Reyes-Robles T., Tawa P., Parker Jr D. L., MacMillan D. W. C. (2022). Small molecule photocatalysis enables drug target identification *via* energy transfer. Proc. Natl. Acad. Sci. U. S. A..

[cit8] Geri J. B., Oakley J. V., Reyes-Robles T., Wang T., McCarver S. J., White C. H., Rodriguez-Rivera F. P., Parker D. L., Hett E. C., Fadeyi O. O., Oslund R. C., MacMillan D. W. C. (2020). Microenvironment mapping *via* Dexter energy transfer on immune cells. Science.

[cit9] Tay N. , Ryu K. A., Weber J., Olow A., Reichman D., Oslund R., Fadeyi O. O. and Rovis T., Targeted Activation in Localized Protein Environments *via* Deep Red Photoredox Catalysis, ChemRxiv, 2021, preprint, 10.26434/chemrxiv-2021-x9bjv, accessed 2022-07-28 PMC9840673 36216892

[cit10] Sato S., Nakamura H. (2013). Ligand-Directed Selective Protein Modification Based on Local Single-Electron-Transfer Catalysis. Angew. Chem., Int. Ed..

[cit11] Li B. X., Kim D. K., Bloom S., Huang R. Y. C., Qiao J. X., Ewing W. R., Oblinsky D. G., Scholes G. D., MacMillan D. W. C. (2021). Site-selective tyrosine bioconjugation *via* photoredox catalysis for native-to-bioorthogonal protein transformation. Nat. Chem..

[cit12] Stephanopoulos N., Francis M. B. (2011). Choosing an effective protein bioconjugation strategy. Nat. Chem. Biol..

[cit13] Hung V., Udeshi N. D., Lam S. S., Loh K. H., Cox K. J., Pedram K., Carr S. A., Ting A. Y. (2016). Spatially resolved proteomic mapping in living cells with the engineered peroxidase APEX2. Nat. Protoc..

[cit14] Tsushima M., Sato S., Nakamura H. (2017). Selective Purification and Chemical Labelling of a Target Protein on Ruthenium Photocatalyst Functionalized Affinity Beads. Chem. Commun..

[cit15] Fancy D. A., Kodadek T. (1999). Chemistry for the Analysis of Protein-Protein Interactions: Rapid and Efficient Cross-Linking Triggered by Long Wavelength Light. Proc. Natl. Acad. Sci. U. S. A..

[cit16] Niederer K. A., Gilmartin P. H., Kozlowski M. C. (2020). Oxidative Photocatalytic Homo-and Cross-Coupling of Phenols: Nonenzymatic; Catalytic Method for Coupling Tyrosine. ACS Catal..

[cit17] Danaher P., Warren S., Dennis L., D'Amico L., White A., Disis M. L., Geller M. A., Odunsi K., Beechem J., Fling S. P. (2017). Gene expression markers of tumor infiltrating leukocytes. J. Immunother. Cancer.

[cit18] Juneau A., Hope T. O., Malenfant J., Mesko M., McNeill J., Frenette M. (2022). Methods to Predict Potential Reagents in Iridium-Based Photoredox Catalysis Calibrated with Stern–Volmer Quenching Rate Constants. ACS Catal..

[cit19] Hernandez-Perez A. C., Collins S. K. (2016). Heteroleptic Cu-based sensitizers in photoredox catalysis. Acc. Chem. Res..

[cit20] Glaser F., Wenger O. S. (2020). Recent progress in the development of transition-metal based photoredox catalysts. Coord. Chem. Rev..

[cit21] Lee Y. B., Lim S., Lee Y., Park C. H., Lee H. J. (2023). Green Chemistry for Crosslinking Biopolymers: Recent Advances in Riboflavin-Mediated Photochemistry. Materials.

[cit22] (b) Grosheva D. and Hyster T. K., Light-driven flavin-based biocatalysis, Flavin-Based Catalysis: Principles and Applications, ed. R. Cibulka and M. Fraaije, Wiley-VCH Verlag GmbH & Co. KGaA, 2021, pp. 291–313, 10.1002/9783527830138.ch12

[cit23] Pitre S. P., McTiernan C. D., Vine W., DiPucchio R., Grenier M., Scaiano J. C. (2015). Visible-light actinometry and intermittent illumination as convenient tools to study Ru(bpy)_3_Cl_2_ mediated photoredox transformations. Sci. Rep..

[cit24] DeRosa M. C., Crutchley R. J. (2002). Photosensitized singlet oxygen and its applications. Coord. Chem. Rev..

[cit25] Thomas M. J., Foote C. S. (1978). Chemistry of singlet oxygen—XXVI. Photooxygenation of phenolsy. Photochem. Photobiol..

[cit26] Scully Jr F. E., Hoigné J. (1987). Rate constants for reactions of singlet oxygen with phenols and other compounds in water. Chemosphere.

[cit27] Al-Nu'airat J., Dlugogorski B. Z., Gao X., Zeinali N., Skut J., Westmoreland P. R., Oluwoye I., Altarawneh M. (2019). Reaction of phenol with singlet oxygen. Phys. Chem. Chem. Phys..

[cit28] Chen W., Chen J. J., Lu R., Qian C., Li W. W., Yu H. Q. (2014). Redox reaction characteristics of riboflavin: a fluorescence spectroelectrochemical analysis and density functional
theory calculation. Bioelectrochemistry.

[cit29] Burton G. W., Ingold K. U. (1986). Vitamin E: application of the principles of physical organic chemistry to the exploration of its structure and function. Acc. Chem. Res..

[cit30] Frenette M., Aliaga C., Font-Sanchis E., Scaiano J. C. (2004). Bond dissociation energies for radical dimers derived from highly stabilized carbon-centered radicals. Org. Lett..

[cit31] Michon T., Chenu M., Kellershon N., Desmadril M., Guéguen J. (1997). Horseradish peroxidase oxidation of tyrosine-containing peptides and their subsequent polymerization: a kinetic study. Biochemistry.

[cit32] Oakley J. V., Buksh B. F., Fernández D. F., Oblinsky D. G., Seath C. P., Geri J. B., Scholes G. D., MacMillan D. W. C. (2022). Radius measurement *via* super-resolution microscopy enables the development of a variable radii proximity labelling platform. Proc. Natl. Acad. Sci. U. S. A..

