# Supplementary material for: Targeted proximity-labelling of protein tyrosines via flavin-dependent photoredox catalysis with mechanistic evidence for a radical–radical recombination pathway
Source: Chem Sci. 2023 May 17;14(26):7327–33. doi: 10.1039/d3sc00638g (PMC10321502; doi:10.1039/d3sc00638g)
Supplement: SC-014-D3SC00638G-s001 [file SC-014-D3SC00638G-s001.pdf]

Electronic Supplementary Material (ESI) for Chemical Science.
This journal is © The Royal Society of Chemistry 2023

Targeted Proximity-Labelling of Protein Tyrosines via Flavin-Dependent Photoredox Catalysis with Mechanistic Evidence for a Radical-Radical Recombination Pathway.

Supporting Information

Taylor O. Hope,[a] Tamara Reyes-Robles,[b] Keun Ah Ryu,[b] Steven Mauries,[a] Nicole Removski,[a] Jacinthe Maisonneuve,[a] Rob C. Oslund*,[b][c] and Olugbeminiyi O. Fadeyi*,[b][c] and Mathieu Frenette*[a]

[a] Department of Chemistry, NanoQAM and Centre Québécois des Matériaux Fonctionnels (CQMF), Université du Québec à Montréal, Montréal, Québec, H3C 3P8, Canada. E-mail: frenette.mathieu@uqam.ca

[b] Exploratory Science Center, Merck & Co., Inc., Cambridge, MA, USA

[c] Current Address: InduPro, Cambridge, Massachusetts, USA

*Corresponding authors.
Mathieu Frenette : frenette.mathieu@uqam.ca
Olugbeminiyi O. Fadeyi : niyi@induprolabs.com
Rob C. Oslund : rob@induprolabs.com

Table of Contents

Targeted and Untargeted Labeling of CD45 on the Cell Surface **3**

Targeted Labeling of CD45 on the Cell Surface with HRP or RFT **4**

Peptide-Phenol Coupling Scope **6**

Absorption and Emission Spectra **7**

Electrochemistry Measurements **8**

Calculation of Excited State Redox Potentials **9**

Fluorescence Lifetime **10**

Relative Fluorescence Quantum Yield **11**

Catalyst Photostability **11**

Time-Resolved Stern-Volmer Quenching Studies and Triplet Excited State Lifetime Measurement **12**

Relative Rate of Reaction Between Phenols and Singlet Oxygen **15**

CD45 labeling on Jurkat cells with CD45 using RFT primary/secondary antibody system **22**

CD45 labeling on Jurkat cells with CD45 using HRP primary/secondary antibody system **23**

LC-MS/MS-based Proteomic Analysis of Labeled Cell Experiments **24**

Bioinformatic Analysis of LC-MS/MS Data **24**

General Synthetic Information **24**

Photoredox Mediated C-H Functionalization of Peptides **25**

Table S4. Optimization on the C-H Functionalization of Phenol Cross-Coupling 25

General Procedure for the Phenol C-H Functionalization **28**

(*S*)-2-acetamido-3-(2',6-dihydroxy-3'-methoxy-5'-methyl-[1,1'-biphenyl]-3-yl)-N-methylpropanamide: 28

(S)-2-acetamido-3-(4-hydroxy-3-(2-methoxy-4-methylphenoxy)phenyl)-N-methylpropanamide (12) was isolated alongside compound 11: 28

(*S*)-2-acetamido-N-((*S*)-1-amino-3-(4',6-dihydroxy-3',5'-dimethoxy-[1,1'-biphenyl]-3-yl)-1-oxopropan-2-yl)-3-phenylpropanamide (13): 29

(S)-2-amino-3-(5'-((S)-3-amino-2-(2-(((benzyloxy)carbonyl)amino)acetamido)-3-oxopropyl)-2',6-dihydroxy-5-methoxy-[1,1'-biphenyl]-3-yl)propanoic acid (14): 29

(4*S*,7*S*,13*S*)-13-(((*S*)-1-amino-1-oxo-3-phenylpropan-2-yl)carbamoyl)-4-((*S*)-sec-butyl)-7-((4',6-dihydroxy-3',5'-dimethoxy-[1,1'-biphenyl]-3-yl)methyl)-2,5,8,11-tetraoxo-3,6,9,12-tetraazahexadecan-16-oic acid (15): 30

(*S*)-2-acetamido-3-(4',6-dihydroxy-3',5'-dimethoxy-[1,1'-biphenyl]-3-yl)-*N*-methylpropanamide (16): 30

DFT Calculations **49**

References **128**

Targeted and Untargeted Labeling of CD45 on the Cell Surface

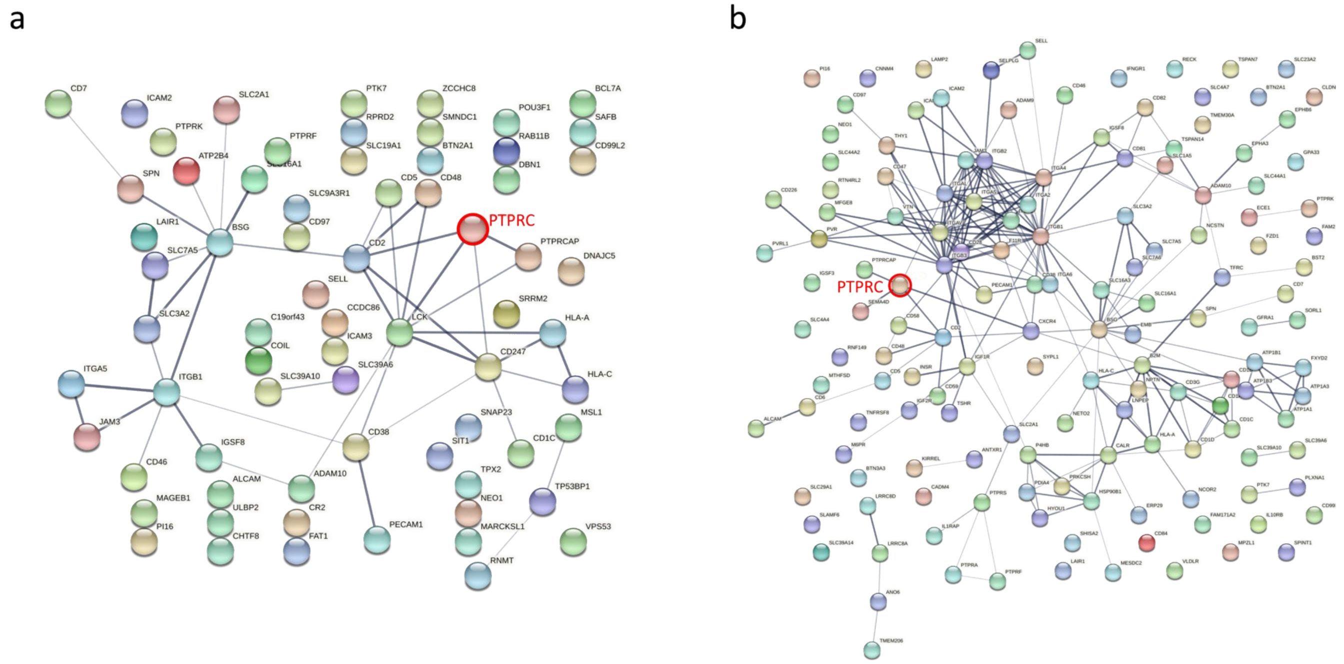

Figure S1. **STRING plot analysis of CD45 (PTPRC)-targeted labeling using RFT-mediated photoproximity labeling.** Analysis using search tool for retrieval of interacting genes (STRING) of significantly enriched proteins from a) RFT-mediated CD45 targeted labeling and b) HRP-mediated CD45 targeted labeling in Figure 1 (p-value < 0.05 and $\log_2$FC > 1.5). CD45 (PTPRC) is highlighted in red for clarity. Thick edges denote protein interactions with supporting experimental evidence. Thin lines represent interactions based on all other sources of evidence as indicated by the STRING database.[1] Known CD45 interactors LCK, CD2, PTPRCAP, SEMA4D, and CXCR4 are identified as part of the enriched protein list across both labeling methods.

Targeted Labeling of CD45 on the Cell Surface with HRP or RFT

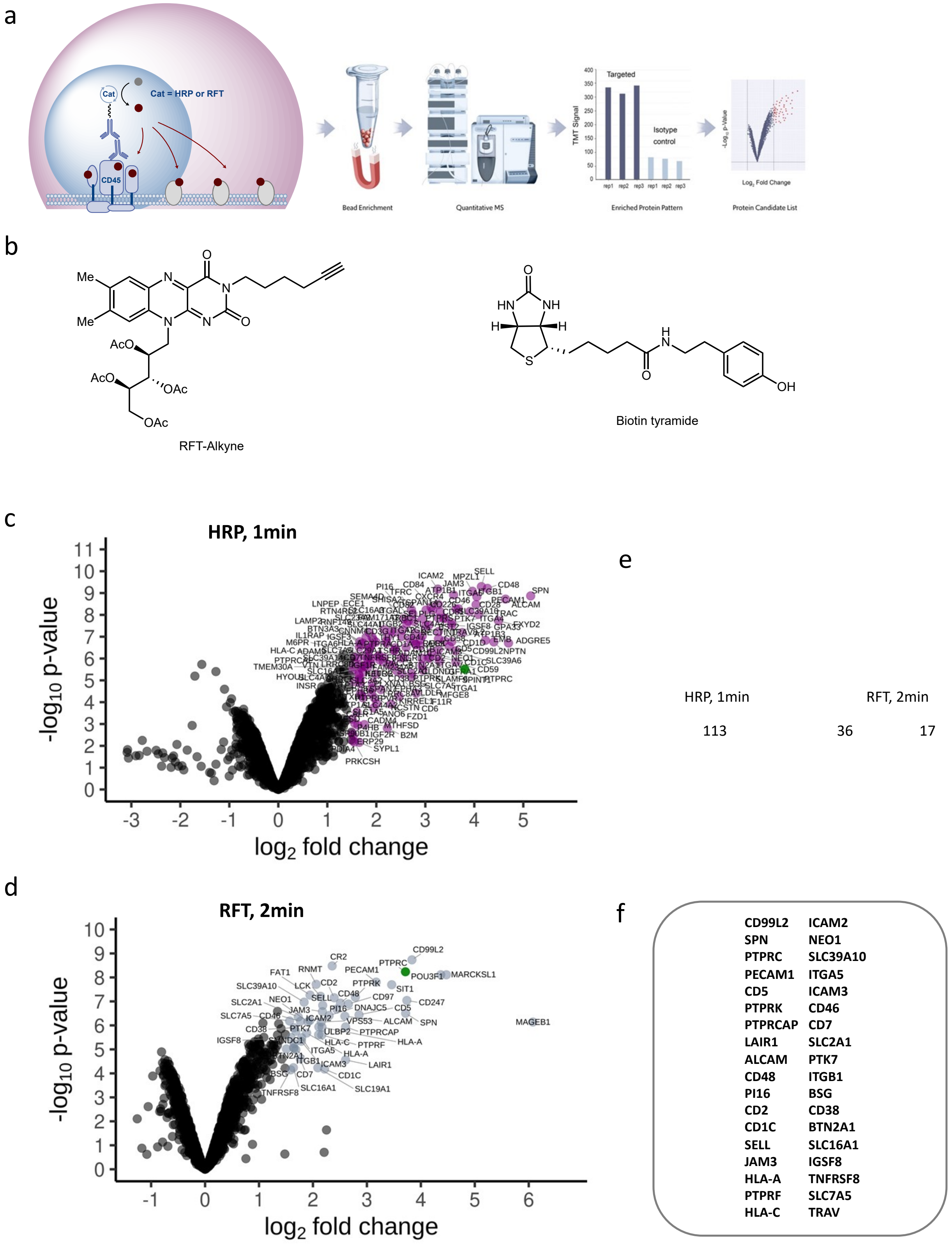

Figure S2. **CD45 (PTPRC)-targeted labeling on Jurkat cells using Peroxidase or RFT-based proximity labeling.** a) Schematic depicting CD45 targeted labeling on Jurkat cells with HRP or RFT followed by downstream protein enrichment and proteomic analysis. b) Structures of the RFT-alkyne and biotin tyramide probe used for targeted and untargeted RFT-labeling experiments. c) Volcano plot of statistical significance vs. fold-enrichment for CD45-targeted vs isotype-targeted biotinylation on Jurkat cells using an anti-CD45 primary antibody/secondary antibody peroxidase conjugate (HRP) or isotype/secondary antibody peroxidase conjugate with 1 minute of labeling in the presence of biotin tyramide and hydrogen peroxide. Significantly enriched proteins (p-value < 0.05 and $\log_2$FC > 1.5) are indicated in purple and CD45 (PTPRC) is labeled in green (n = 3 experiments). d) Volcano plot of statistical significance vs. fold-enrichment for CD45-targeted vs isotype-targeted biotinylation on Jurkat cells using an anti-CD45 primary antibody/secondary antibody RFT conjugate (RFT) or isotype/secondary antibody RFT conjugate with 2 minutes of visible light activation in the presence of biotin tyramide. Significantly enriched proteins (p-value < 0.05 and $\log_2$FC > 1.5) are indicated in light blue and CD45 (PTPRC) is labeled in green (n = 3 experiments). e) Venn diagram of significantly enriched proteins from targeted labeling with Peroxidase (purple circle) or RFT (light blue circle). f) List of proteins that overlap from the HRP and RFT methods in panel e.

Peptide-Phenol Coupling Scope

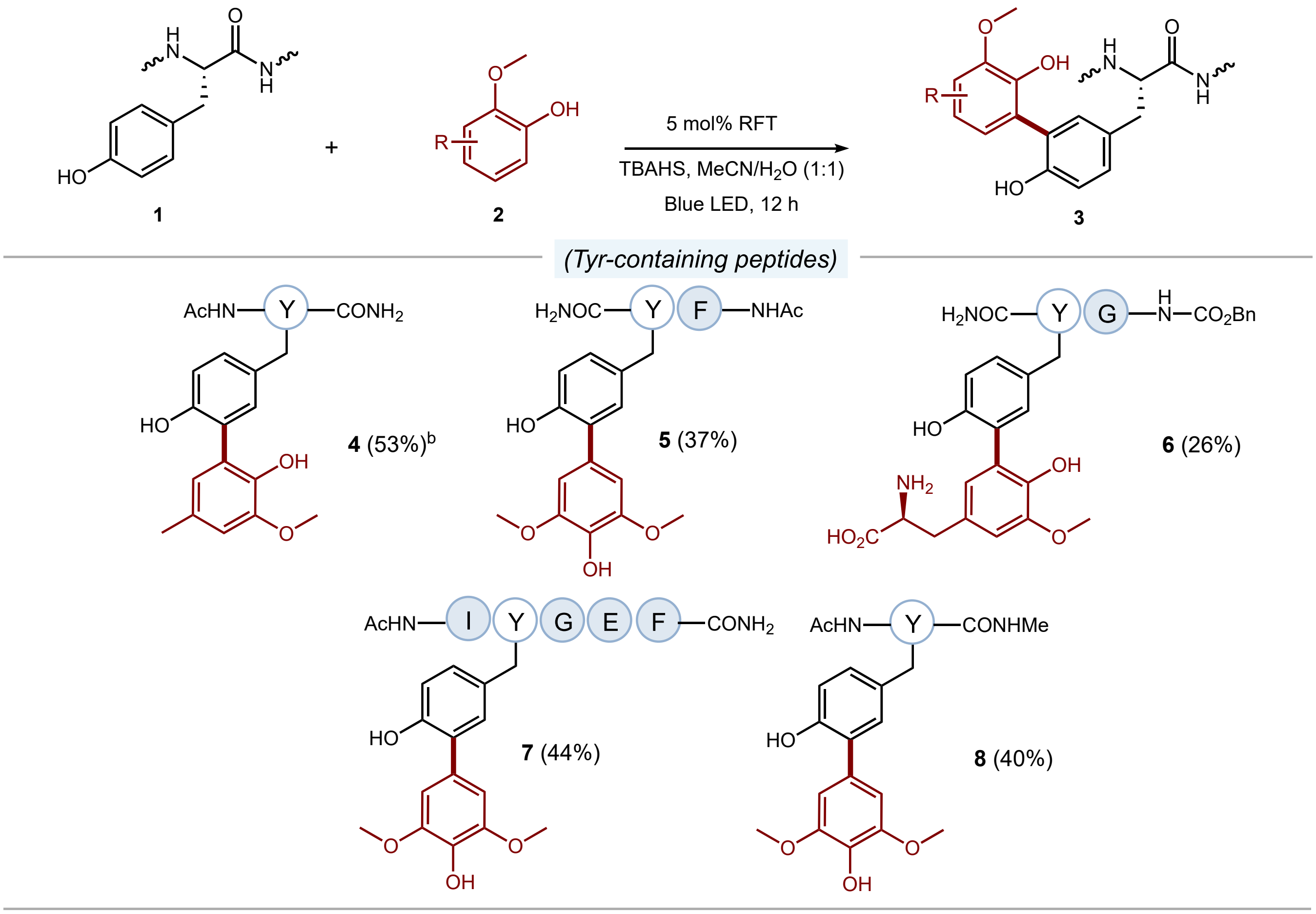

[a] 10 mol% of tetrabutylammonium hydrogen-sulfate (TBAHS).
[b] 22% yield of compound **4** when reaction performed without TBAHS.

Figure S3. RFT-mediated coupling substrate scope.

Absorption and Emission Spectra

Absorption and emission spectra were recorded by first collecting a blank of 1:1 water:acetonitrile in a quartz cuvette (1 x 1 cm). Emission spectra were collected using a step size of 2 nm and an integration time of 0.3 seconds. The slits were set to 0.50 mm 0.50 mm 0.50 mm 0.50 mm.

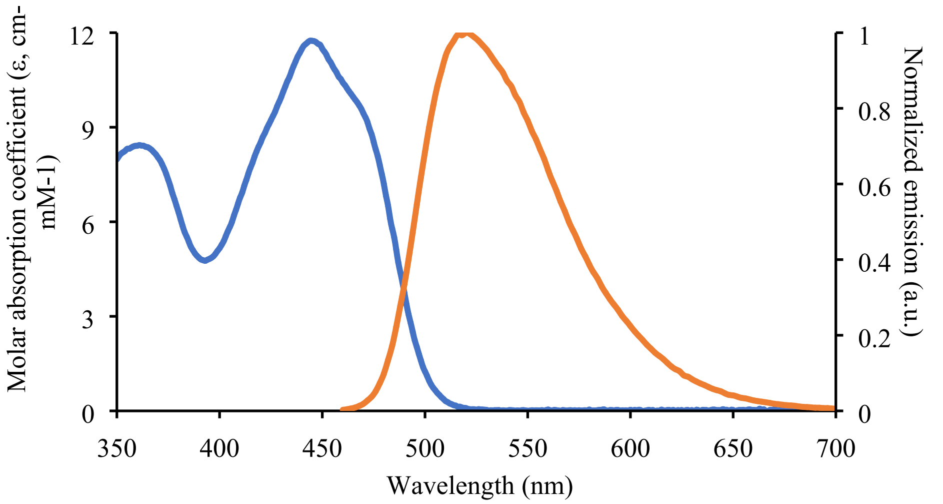

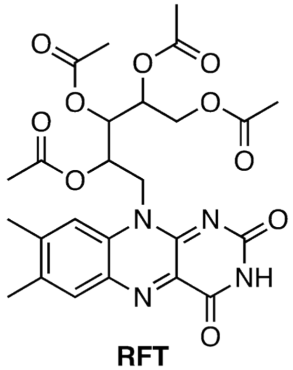

RFT

λ_{ex} = 445 nm

ε = 11638 $M^{-1} s^{-1}$

λ_{00} = 488 nm

(E_{00} = 2.54 eV)

λ_{em} = 520 nm

Φ_{fluor} = 0.31

$\tau_{0\ fluor}$ = 3.2 ns

Figure S4. Normalized absorption (blue) and emission (solvent corrected, orange λ_{ex} = 445 nm) spectrum of **RFT** in 1:1 water:acetonitrile.

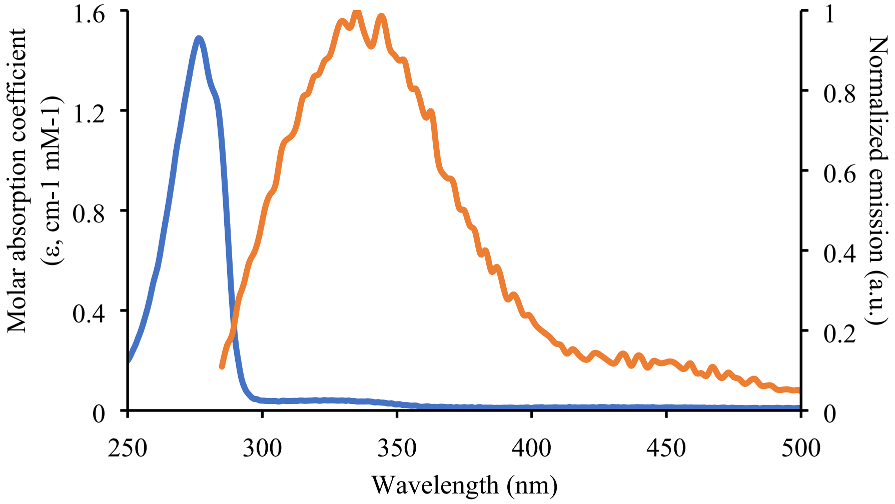

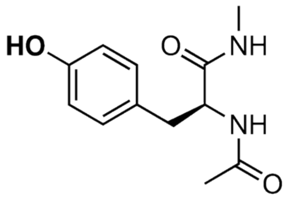

λ_{max} = 276 nm

(ε = 1471 M^{-1} cm^{-1})

λ_{em} = 335 nm

Figure S5. Normalized absorption (blue) and emission (solvent corrected, orange λ_{ex} = 276 nm) spectrum of **Ac-Tyr-NHMe** in 1:1 water:acetonitrile.

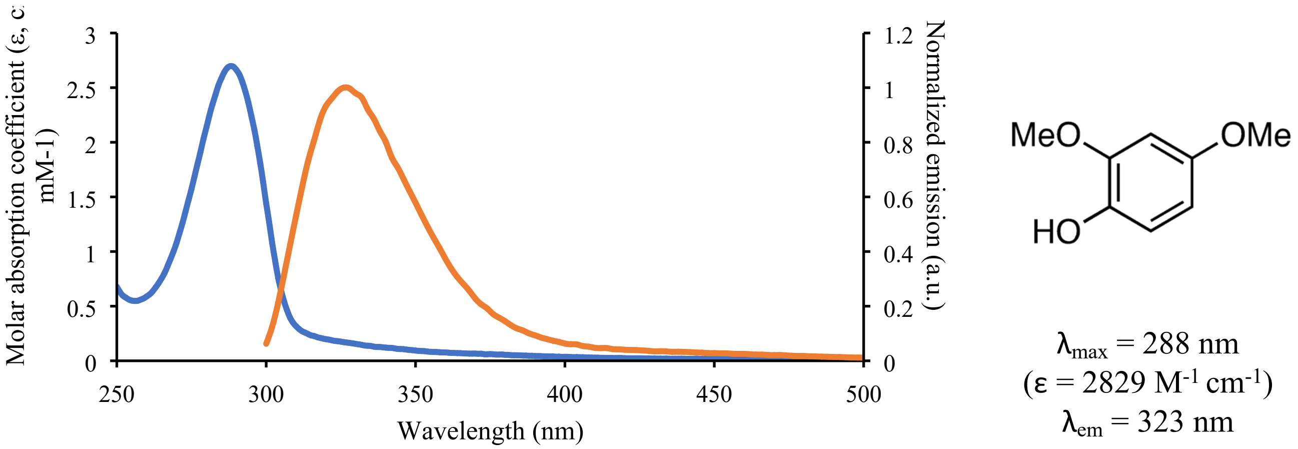

Figure S6. Normalized absorption (blue) and emission (solvent corrected, orange λ_{ex} = 288 nm) spectrum of **2,4-dimethoxyphenol** in 1:1 water:acetonitrile.

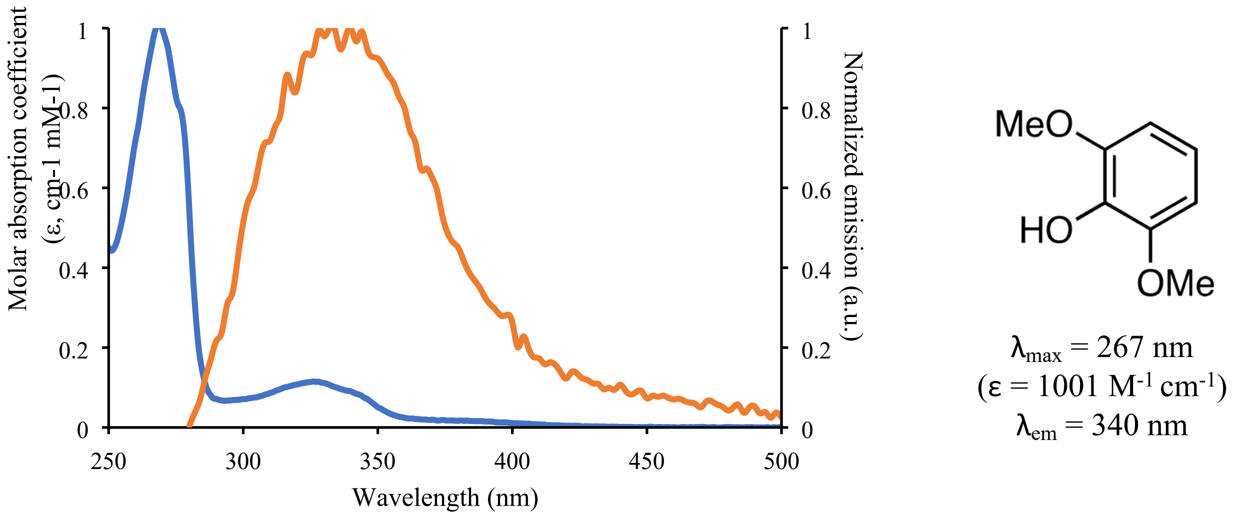

Figure S7. Normalized absorption (blue) and emission (solvent corrected, orange λ_{ex} = 267 nm) spectrum of **2,6-dimethoxyphenol** in 1:1 water:acetonitrile.

Electrochemistry Measurements

We experimentally validated just how strong of an oxidizing agent the excited state triple is by examining the single electron transfer (SET) step between ^{3}[RFT]* and phenol via cyclic voltammetry. A solution of tetraethylammonium BF_4 (0.09 M, as the supporting electrolyte), was prepared in 1:1 water:acetonitrile and degassed with N_2. The cyclic voltammograms were measured using an Ag/AgCl pseudo-reference electrode, a gold working electrode, and a platinum counter electrode. Ferrocene was used as an internal potential reference system and the scanning rate was 50 mV/s. For re-calculation, Fc/Fc^+ was taken to be 0.380 V vs. SCE. RFT was measured to have a reduction potential of -0.185 V vs SCE (**Figure S8**).

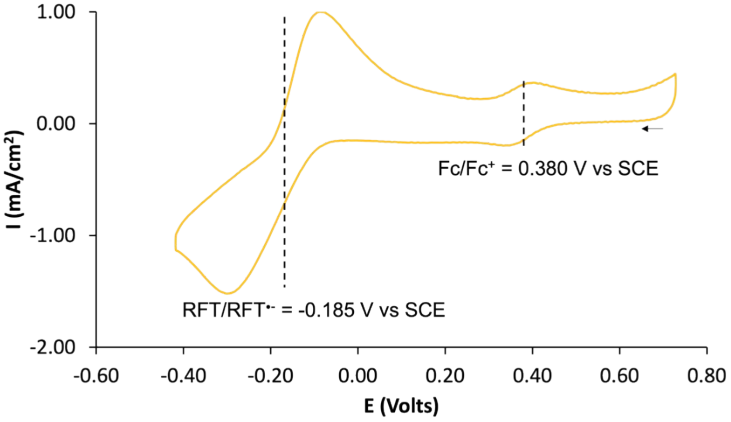

Figure S8. Cyclic voltammetry of **RFT** (0.25 mM).

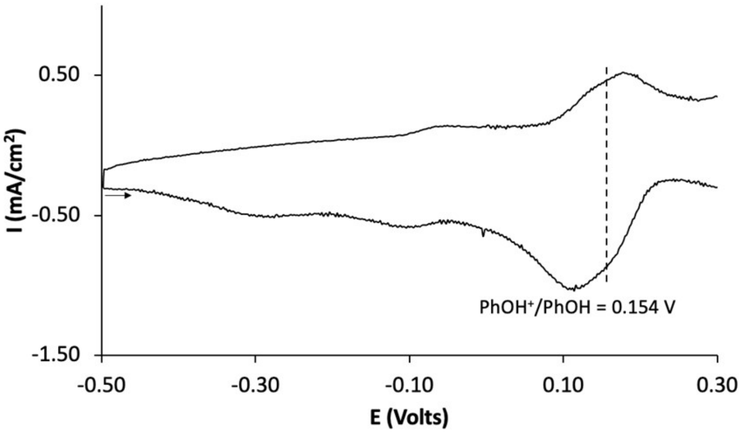

Figure S9. Cyclic voltammetry of 2,6-dimethoxyphenol (0.5 mM).

Calculation of Excited State Redox Potentials

The excited state redox potentials were calculated using the Rehm and Weller equation[2]:

$$E^*_{1/2}{}^{red} = E_{1/2}{}^{red} + E_{00}$$

$$E^*_{1/2}{}^{oxi} = E_{1/2}{}^{oxi} - E_{00}$$

The excited state potential is the sum of the ground state reduction potential of **RFT** ($E_{1/2}{}^{red}$) and the energy difference between the ground state and the excited state (E_{00}). This refers to the transition from the lowest energy vibrational state, which is estimated to the intersection between the absorption and fluorescence spectra after converting wavelength to electron volts Figure S4 and Table S1.[3] Although ^{1}[**RFT**]*, is higher in energy than ^{3}[**RFT**]*, and will produce a stronger oxidant, **RFT** is known to undergo ISC to the triplet. The triplet has a significantly longer lifetime than the singlet which results in the active oxidizing agent being ^{3}[**RFT**]*. In acetonitrile:water (1:1 v/v) ^{3}[**RFT**]* has an oxidizing potential of 2.35 V (*vs* SCE). The oxidation of phenols by ^{3}[**RFT**]*, is generally thermodynamically favorable—for

example, the tyrosine/tyrosyl radical redox potential is 1.08 V (*vs* SCE),[4,5] which makes tyrosine oxidation by ^{3}[**RFT**]* favorable by 1.27 V.

Table S1. Summary of redox potentials and excited state redox potentials for **[RFT]***

$E_{1/2}^{red}$(RFT/RFT$^{\bullet-}$) vs SCE	E_{00} eV (488 nm)	$E^*{}_{1/2}^{red}$(RFT*/RFT$^{\bullet-}$) Vs SCE
-0.19	2.54	2.35

Fluorescence Lifetime

Fluorescence lifetime measurement was performed using time resolved fluorescence on a PTI QuantaMaster 40 (Horiba). In a 1 x 1 cm quartz cuvette a solution of RFT (0.0093 mM in 1:1 water:acetonitrile) was excited at 456 nm and the emission was measured at 520 nm; the irritation wavelength was chosen due to the limited selection of pulsed LED lights available. To the trace a mono-exponential decay was fitted and corrected for the instrument response factor (IRF) using LUDOX® and setting the emission wavelength to the excitation wavelength (456 nm). The fluorescence lifetime of **RFT** in 1:1 water:acetonitrile is 3.2 ns.

The lifetime was measured with a start delay of 55 ns and an end delay of 90 ns. The integration was set to 20 seconds in random collection mode with the slits at 1.00 mm.

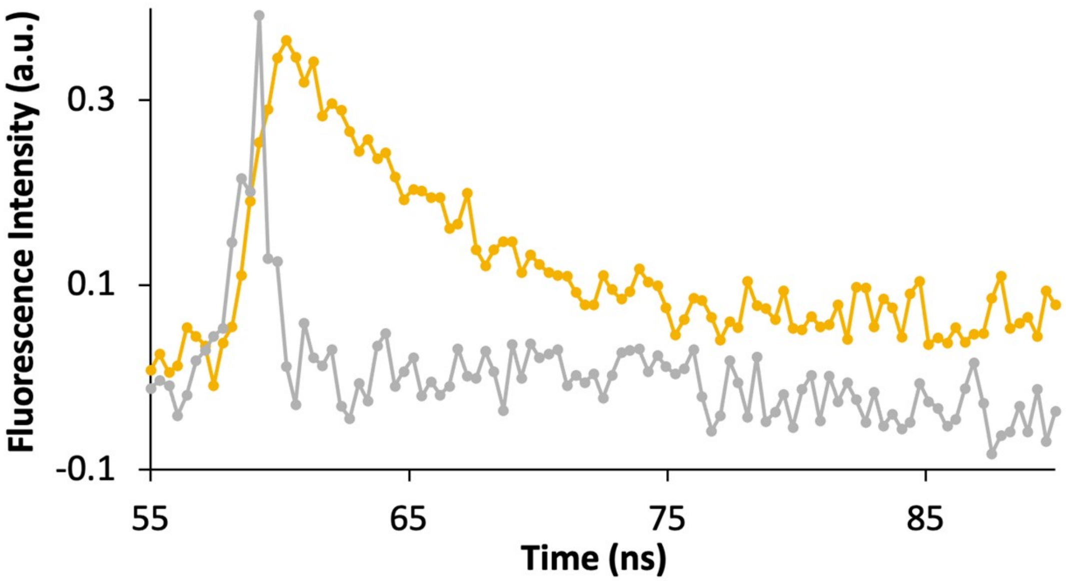

Figure S10. Fluorescence lifetime measurement of **RFT** (orange) in 1:1 water:acetonitrile and the IRF (grey).

Relative Fluorescence Quantum Yield

The relative fluorescence quantum yield of **RFT** was determined by comparing an unknown quantum yield to that of fluorescein which has a known absolute quantum yield of 0.925.[6] The slits were set to 0.5 mm corresponding to a 2 nm resolution.

In a 1 x 1 cm quartz cuvette, emission spectra were recorded at 450 nm, first collecting a blank to correct for any emission arising from the solvent. The samples were then added into the cuvettes in small increments, without exceeding an absorption of 0.1 to minimize non-linear effects. The blank for fluorescein is 0.1M NaOH, and for **RFT** the blank used was the solvent (1:1 water:acetonitrile). The integrated fluorescence was then graphed vs the absorbance at 450 nm (Figure S11) and based on the equation below the fluorescence quantum yield of RFT was found to be 0.31.

The relative quantum yield was then calculated using the equation:

$$\Phi_{RFT} = \Phi_{std} * \left(\frac{m_{RFT}}{m_{std}}\right) * \left(\frac{\eta_{RFT}}{\eta_{std}}\right)^2$$

Where Φ is quantum yield, m is the gradient of the plot of integrated fluorescence intensity against absorbance, and η is the refractive index of the solvent.[7] The refractive index for 0.1 M NaOH is 1.33 (water) and for acetonitrile is 1.34.

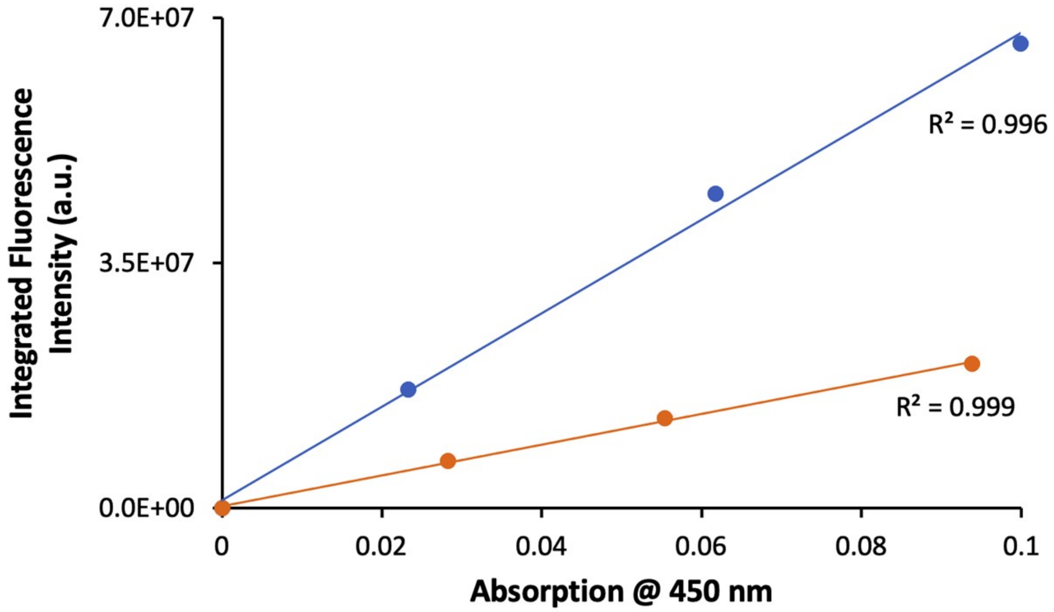

Figure S11. Integrated fluorescence intensity of **RFT** (orange) and fluorescein (blue).

Catalyst Photostability

Photocatalyst stability was measured using the time-based option on the PTI QuantaMaster 40 (Horiba). In a 1 x 1 cm quartz cuvette a solution of **RFT** ($A_{445\ nm}$ = 0.093 in 1:1 water:acetonitrile) with constant stirring via a stir bar was excited at 445 nm and the emission was measured at 520 nm. The scan took place over 7200 seconds, collecting ~1 data point every minute. The slits were set to 0.25 mm 0.25 mm 4.00

mm 4.00 mm.

The scans were also run with only solvent to ensure that the change in photocatalyst stability was due to the change in fluorescence of the photocatalyst, and a final test was done with **LUDOX**® (Sigma-Aldrich) in the cuvette to ensure that the light intensity remained constant over the 2 hour scan where the excitation wavelength was set to 445 nm and emission wavelength was set to 458 nm, and the slits were adjusted to match the initial fluorescence intensity (IRF).

RFT photostability was measured in the presence and absence of various phenol (2,6-dimethoxyphenol and 2,4-dimethoxyphenol, 84.4 mM in 1:1 water:acetonitrile), the results are summarized in the graph below.

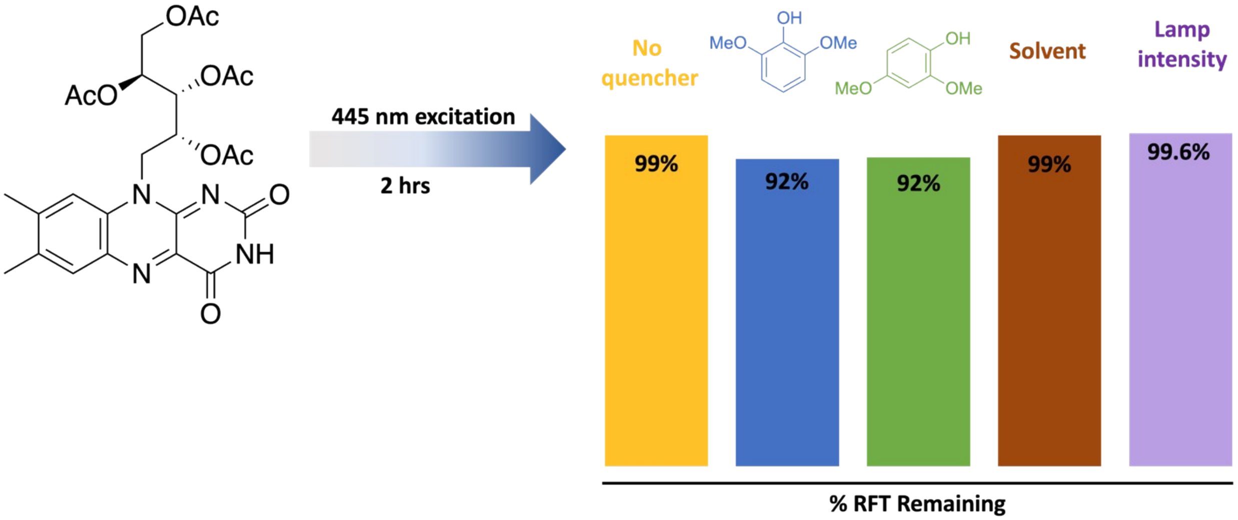

Figure S12. RFT Photocatalyst stability over 2 hours with and without phenols.

Time-Resolved Stern-Volmer Quenching Studies and Triplet Excited State Lifetime Measurement

The excited state lifetime of RFT was measured after pulsed laser irradiation at 355 nm (~20 mJ/pulse, ~5 ns pulse duration) from the third harmonic of a Nd:YAG laser (Continuum). The time-resolved emission following the laser excitation was measured at 700 nm using a fiber-optic based laser-flash photolysis system (miniLFP from Luzchem Research, Ottawa, Canada). Pre-degassed phenol quencher was added neat via syringe to a degassed 1:1 water:acetonitrile solution containing RFT (0.06 mM $A_{355} = 0.5$).

For the BSA solutions, 0.101 mM, 0.204 mM and 0.297 mM BSA (16.6 mg, 33.2 mg, and 49.8 mg. respectively) were added to a degassed solution of RFT (0.07 mM, 2 mg in 50 mL PBS, $A_{355} = 0.3$) to a final volume of 2.5 mL and sealed with a rubber septum under vacuum and N_2.

The transient absorption decay monitored at 700 nm fits to mono-exponential decays and the resulting lifetimes were analyzed using Stern-Volmer quenching kinetics analysis, $\tau_0/\tau = 1 + k_q\tau_0[\text{phenol}]$, where τ_0 and τ are the observed lifetime without and with added phenol. The bimolecular rate constants k_q between RFT and various phenols are summarized in Table S2. The excited state lifetime of RFT in 1:1

water:acetonitrile is 12 μs and in PBS is 16 μs.

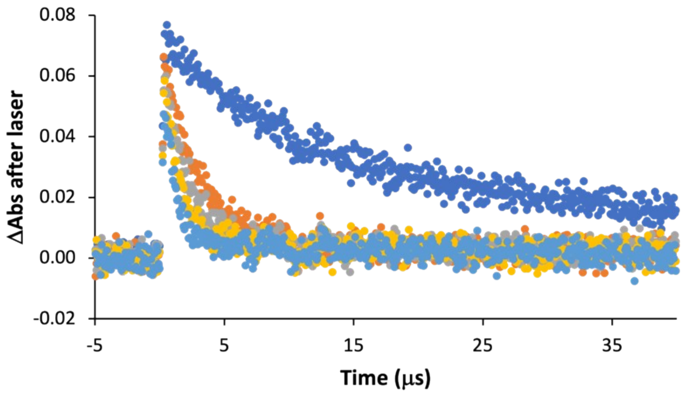

Figure S13. Excited-state lifetime measurements for **^{3}[RFT]*** (λ_{mon} = 700 nm) in the presence of **2,6-dimethoxyphenol** (— 0, — 0.09, — 0.15, — 0.21, — 0.30 mM) in 1:1 water:acetonitrile under argon atmosphere.

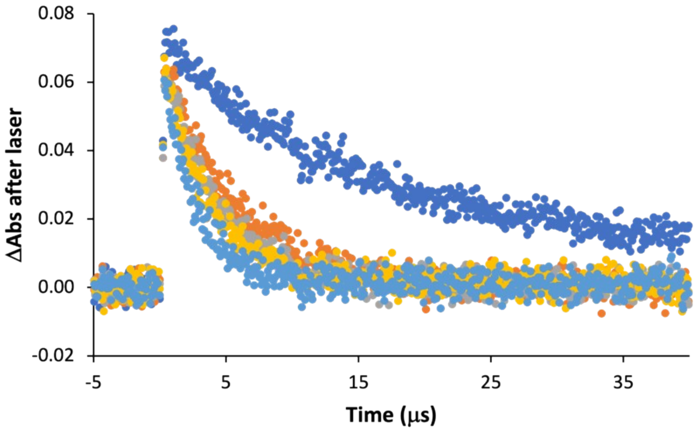

Figure S14. Excited-state lifetime measurements for **^{3}[RFT]*** (λ_{mon} = 700 nm) in the presence of **Ac-Tyr-NHMe** (— 0, — 0.09, — 0.15, — 0.21, — 0.30 mM) in 1:1 water:acetonitrile under argon atmosphere.

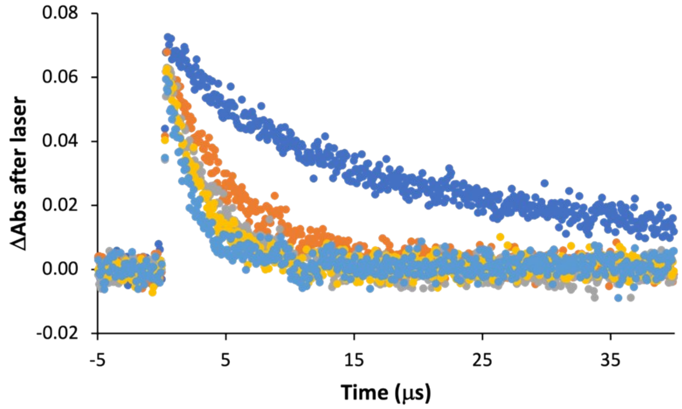

Figure S15. Excited-state lifetime measurements for **^{3}[RFT]*** (λ_{mon} = 700 nm) in the presence of **biotin tyramide** (— 0, — 0.09, — 0.15, — 0.21, — 0.30 mM) in 1:1 water:acetonitrile under argon atmosphere.

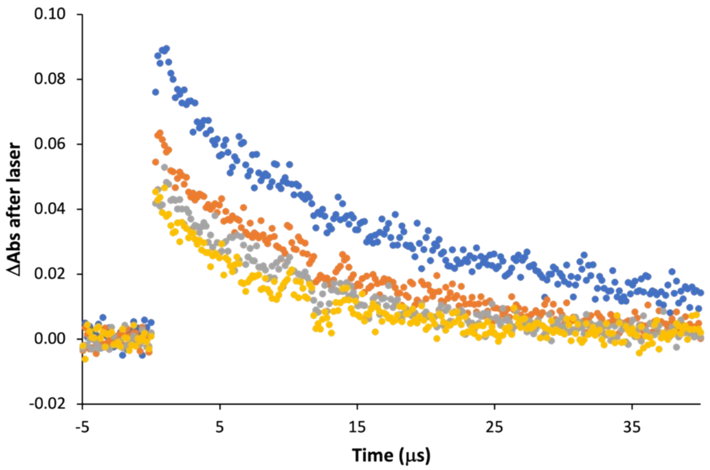

Figure S16. Excited-state lifetime measurements for **^{3}[RFT]*** (λ_{mon} = 700 nm) in the presence of **BSA** (— 0, — 0.101, — 0.204, — 0.297 mM) in PBS under argon atmosphere.

Table S2. Bimolecular rate constants (k_q) between ^{3}[RFT]* and various phenol-containing substrates

**^{3}[RFT]* + phenol**	**k_q (M^{-1} s^{-1})**
2,6-dimethoxyphenol	2.9×10^9
biotin tyramide	1.4×10^9
Ac-Tyr-NHMe	1.1×10^9
Bovine Serum Albumin*	2.4×10^8

*Rate constant measured in PBS

Transient absorption spectra of biotin tyramide (A_{266} = 0.5) measured in deoxygenated phosphate buffer saline (pH = 7.4, with 10% acetonitrile for solubility) after pulsed laser irradiation at 266 nm (~27 mJ/pulse) from the fourth harmonic of a Nd:YAG laser (Continuum). Direct photolysis of the phenol at this wavelength forms a phenoxyl radical that can be followed by its absorption at 430 nm as measured using a fiber-optic based laser-flash photolysis system (miniLFP from Luzchem Research, Ottawa, Canada). The transient absorption decay monitored at 430 nm fits to a second order decays i.e., implies a phenoxyl radical-radical recombination mechanism.

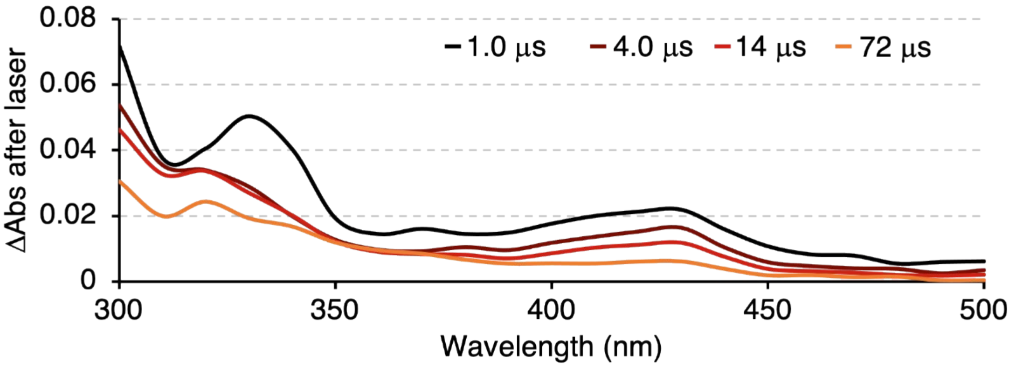

Figure S17. Change in absorption following a pulsed 266 nm laser excitation (27 mJ/pulse) of biotin tyramide (A_{266} = 0.52) in deoxygenated PBS buffer saline (with 10% acetonitrile for solubility)

Relative Rate of Reaction Between Phenols and Singlet Oxygen

Experiments were performed using 16 W blue outdoor LED by Feit Electricity. Quartz cuvettes were held by an in-house built support secured to a platform shaker set at 360 rpm, air-cooled using a fan. The optimization of the reaction setup was tested with a known protocol used to measure chemical actinometry. $Ru(bpy)_3(PF_6)_2$ was used as opposed to $Ru(bpy)_3Cl_2$ and the results were assumed to be unchanged.[8] The experiments were performed in a quartz cuvette (1 x 1 cm) using a Cary-60 to record the absorption spectra. When 4 LEDs were tested, the change in absorption at 372 nm was found to decay too quickly, while only using 1 LED did not give reproducible results for all 4 cuvettes. Using 2 LEDs gave the most reasonable timeframe for the change in absorption at 327 nm, and all 4 cuvettes gave more reproducible results.

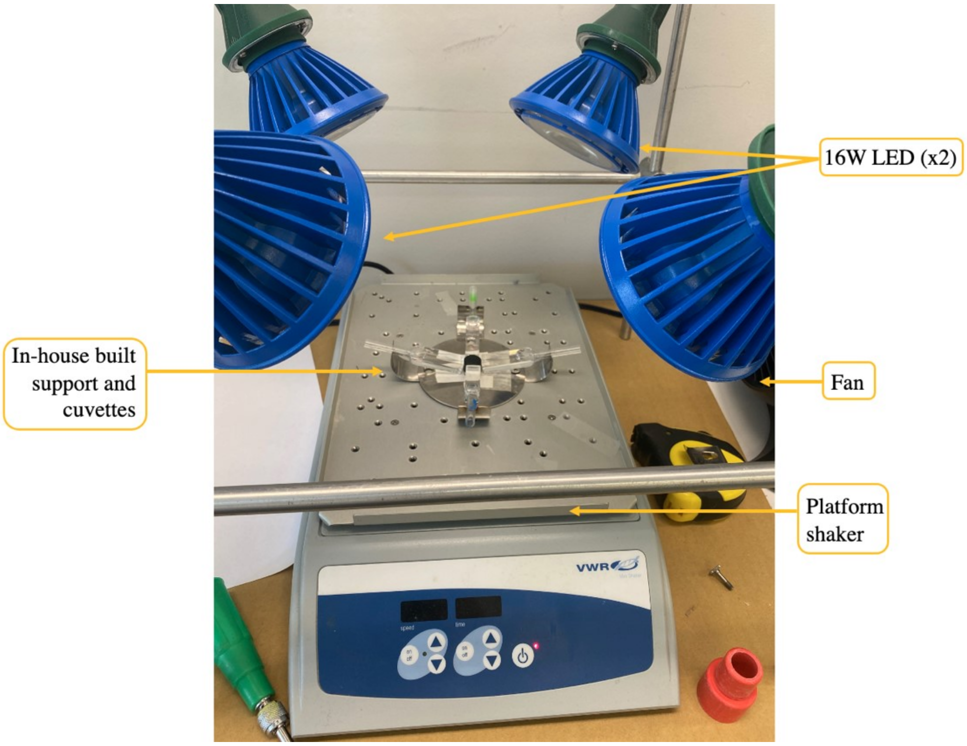

Figure S18. Experimental Setup.

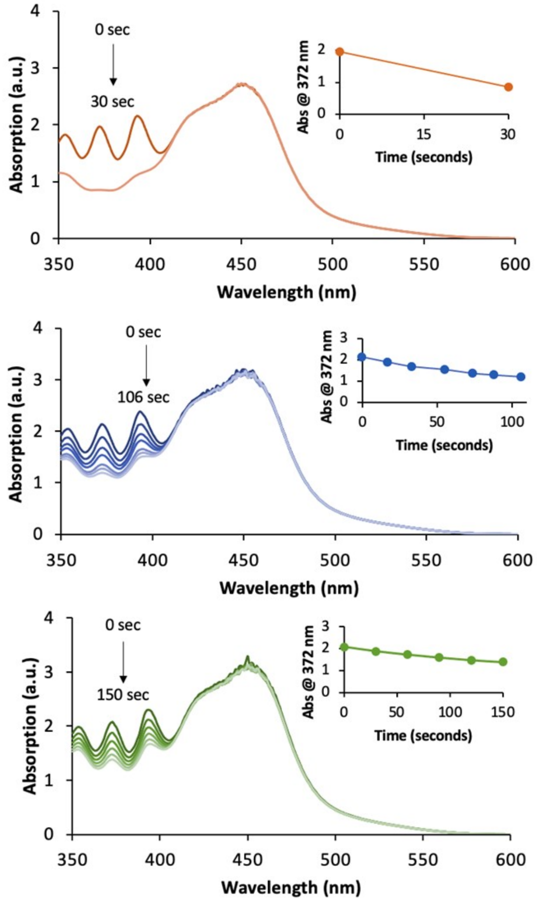

Figure S19. LED intensity optimization using 4 LEDs (orange), 2 LEDs (blue) and 1 LED (green).

With an optimized light setup in hand, the next issue was the solubility of **DPA**, which is poorly soluble in 1:1 Water:acetonitrile. **DPA** was dissolved in acetonitrile and added to a cuvette containing 3 mL 1:1 water:acetonitrile. The change in absorption of **DPA** at 372 nm is linear in the region of interest and this allowed us to proceed to competitive singlet oxygen kinetics.

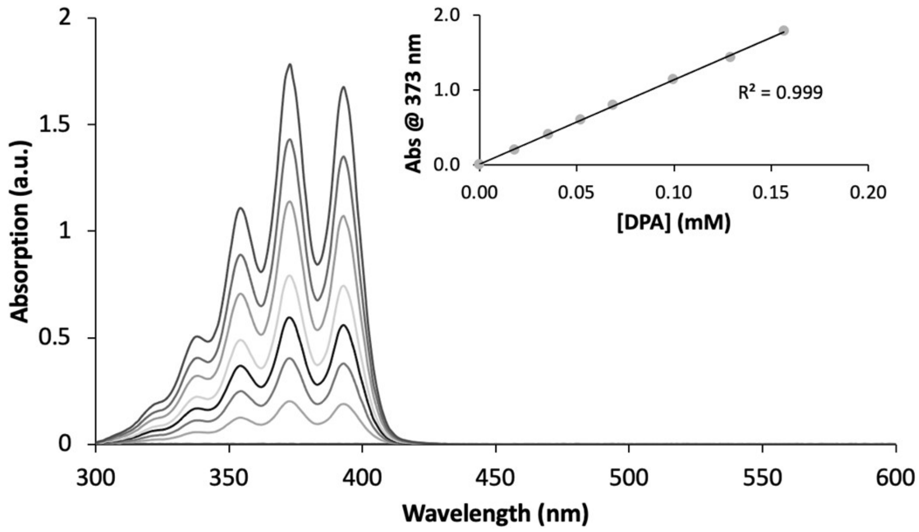

Figure S20. Increasing absorption of **DPA** added to a cuvette of 1:1 water:acetonitrile from a MeCN stock solution to ensure a linear increase at 372 nm. Inset: absorption at 372 nm vs concentration of DPA.

A solution (2.5 mL) of **DPA** (0.1 mM) and **RFT** (A_{454nm} = 0.4, 42 µM) was added to a quartz cuvette under air in 1:1 water:acetonitrile. The cuvettes were irradiated and the change in absorption at 372 nm due to singlet oxygen ($^1O_2$) reaction with **DPA** was recorded.

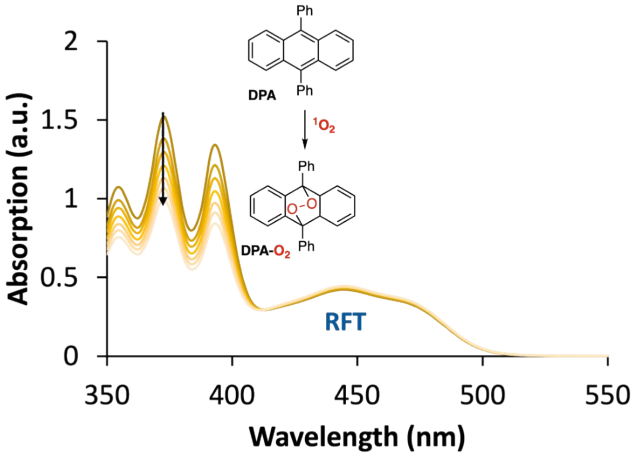

Figure S21. Absorption spectra of **DPA** (initial concentration = 0.1 mM) and **RFT** (initial concentration = 42 µM) during 85 seconds of blue-light irradiation—the absorption decrease is caused by the loss of DPA's π conjugation as it reacts with singlet oxygen, $^1O_2$, via a [4+2] cycloaddition at a known rate constant (k_q = 2.0 x 10^6 M^{-1} s^{-1}).

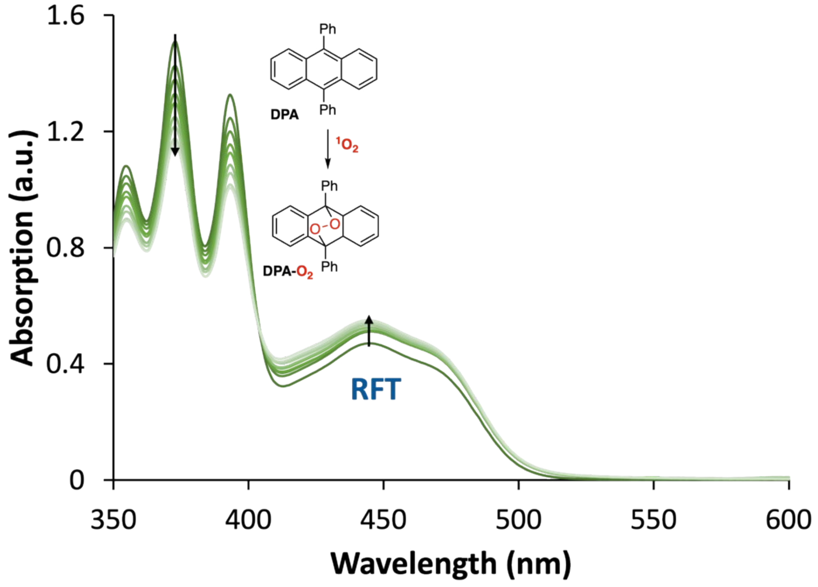

Figure S22. Example of the simultaneous increase in absorption of **RFT** and decrease in absorption of **DPA** during blue-light irradiation caused by the cycloaddition of singlet oxygen and breaking of π-system conjugation. Impure reduced **RFT** oxidizes to the neutral form during irradiation causing the absorption of **RFT** to increase over 160 seconds. [RFT] = 0.042 mM, [DPA] = 0.1 mM and [2,6-dimethoxyphenol] = 1.31 mM (green cuvette).

The absorption decrease seen at short wavelengths in Figure S21 is caused by the loss of **DPA**'s π conjugation as it reacts with singlet oxygen, $^1O_2$, via a [4+2] cycloaddition at a known rate constant (k_q = 2.0 x 10^6 M^{-1} s^{-1}).[8] The rate of this disappearance is slower than that seen in Figure S22 since some of the singlet oxygen will react with 2,6-dimethoxyphenol in this competitive kinetics experiment. Strangely, there was also a slight absorption increase accounted for by the apparent *formation* of RFT during the irradiation. We postulate the increase of RFT is due to the oxidation of reduced RFT impurities in the photocatalyst, e.g., RFT-H_2, during irradiation. The reaction product between $^1O_2$ and phenols—HOO*—could explain the oxidation reaction and absorption increase. To properly quantify the change in DPA concentration during irradiation, it became necessary to deconvolute the contribution of **DPA**, **RFT** and phenol from the absorption spectra (Figure S23).

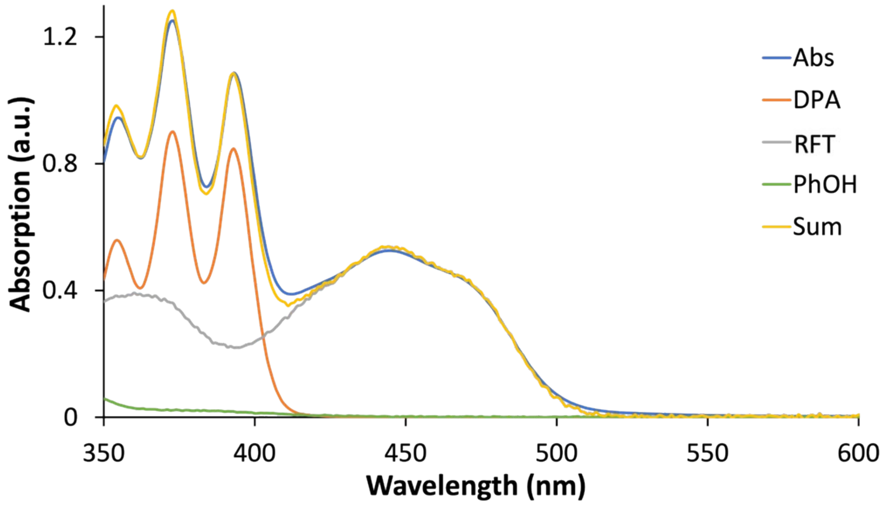

Figure S23. Deconvolution of a UV-visible absorption spectrum (using 80 second irradiation time point as an example) using spectra corresponding to [**RFT**] = 0.042 mM, [**DPA**] = 0.1 mM and [2,6-dimethoxyphenol] = 1.31 mM (green cuvette). Similar deconvolutions were done for each irradiation time point to obtain the change in concentration of **DPA** over time.

To deconvolute the spectra we compared the sum of the absorption of each individual molecule using its molar absorption coefficient to recreate the absorption spectra and solve for the smallest difference in the absorption and sum by changing the concentration of **DPA** and **RFT**. (i.e. solving the (sum-abs)^2 and minimizing the concentration of **DPA** and **RFT** using the “solver” tool in excel).

The rate constant for the reaction between $^1O_2$ and **DPA** in acetonitrile, $k_q = 2.0 \times 10^6$ M^{-1} s^{-1},[8] was estimated to remain unchanged when in 1:1 water:acetonitrile. Fortunately, the excited state lifetime of singlet oxygen ($k_d = 4.5 \times 10^4$ s^{-1}, and τ_0 = 22 μs) is known in 1:1 water:acetonitrile.[9] The following competitive reactions for the reaction with singlet oxygen will take place:

$$^1O_2 \xrightarrow{k_d} {}^3O_2 \qquad k_d = 4.5 \times 10^4\ s^{-1}$$

$$^1O_2 + \textbf{DPA} \xrightarrow{k_q} \text{DPA-}O_2 \qquad k_q = 2.0 \times 10^6\ M^{-1}\ s^{-1}$$

$$^1O_2 + \text{PhOH} \xrightarrow{k_r} \text{PhO}^\bullet \qquad k_r = ?$$

When phenol is added to the cuvette (1.31 mM, 0.66 mM, and 0.33 mM) a new reaction pathway for $^1O_2$ is introduced to form phenoxyl radicals[10] and the change in absorption of **DPA** at 372 nm as a function of irritation time decreases. The rate of reaction between singlet oxygen and phenol is estimated to be:

$$\frac{Slope_{DPA}}{Slope_{DPA\,+\,PhOH}} = \frac{\Phi_0}{\Phi} = 1 + \frac{k_r[PhOH]}{k_d + k_q[DPA]}$$

EQ DERIVATIONS

$$\tau_0 = \frac{1}{k_d + k_q[DPA]}$$

$$\tau_{PhOH} = \frac{1}{k_d + k_q[DPA] + k_r[PhOH]}$$

$$\frac{\tau_0}{\tau_{PhOH}} = \frac{k_d + k_q[DPA] + k_r[PhOH]}{k_d + k_q[DPA]} = 1 + \frac{k_r[PhOH]}{k_d + k_q[DPA]}$$

$$\Phi_0 = fraction\ of\ excited\ states\ that\ react\ with\ DPA\ in\ the\ absence\ of\ PhOH$$
$$\Phi_{PhOH} = fraction\ of\ excited\ states\ that\ react\ with\ DPA\ in\ the\ presence\ of\ PhOH$$

$$\Phi_0 = \frac{k_q[DPA]}{k_d + k_q[DPA]}$$

$$\Phi_{PhOH} = \frac{k_q[DPA]}{k_d + k_q[DPA] + k_r[PhOH]}$$

$$\frac{\Phi_0}{\Phi_{PhOH}} = \frac{k_d + k_q[DPA] + k_r[PhOH]}{k_d + k_q[DPA]} = 1 + \frac{k_r[PhOH]}{k_d + k_q[DPA]}$$

$$\frac{\tau_0}{\tau_{PhOH}} = \frac{\Phi_0}{\Phi_{PhOH}}$$

$$\frac{\Phi_0}{\Phi_{PhOH}} = \frac{-\Delta[DPA]_0/\Delta t}{-\Delta[DPA]_{PhOH}/\Delta t} = \frac{Slope_{DPA}}{Slope_{DPA+PhOH}}$$

$$\frac{Slope_{DPA}}{Slope_{DPA+PhOH}} = 1 + \frac{k_r[PhOH]}{k_d + k_q[DPA]}$$

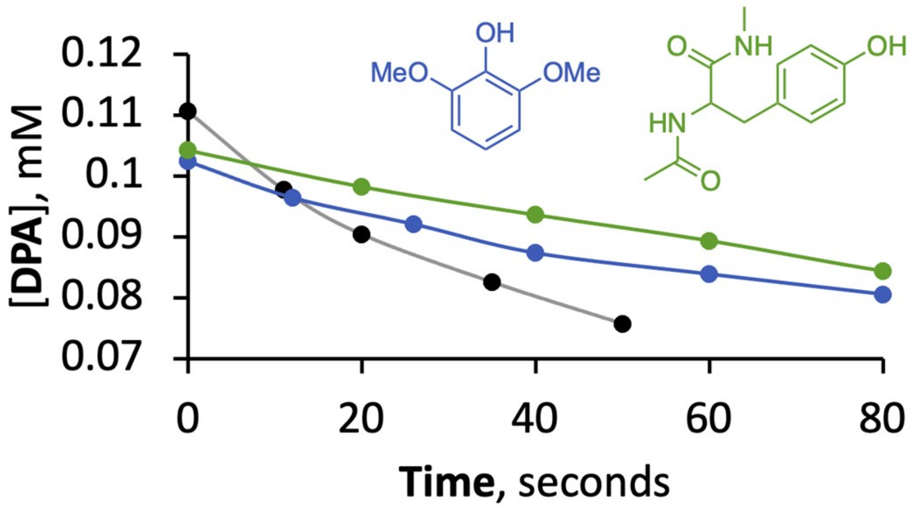

Figure S24. Concentration of **DPA** during irradiation of **RFT** (42 μM) and **DPA** (~ 0.1 mM) in 1:1 water:acetonitrile. Conditions: Gray trace, no phenol added; blue trace, addition of 1.31 mM 2,6-dimethoxylphenol; green trace, addition of 1.3 mM Ac-Tyr-NHMe.

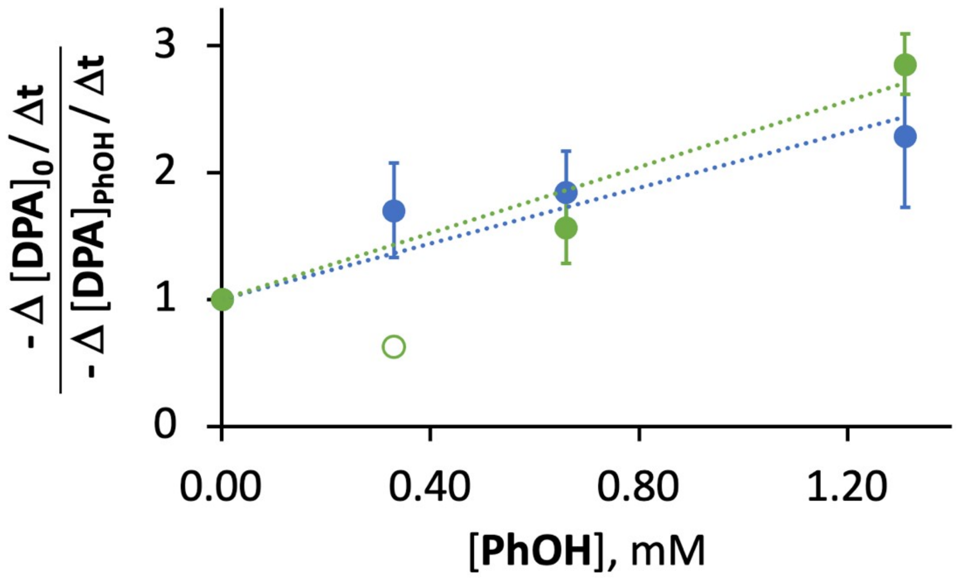

Figure S25. Ratio for the initial rates of consumption of **DPA** without and with phenol during photolysis in the presence of **RFT** as a function of phenol concentration (green = Ac-Tyr-NHMe, blue = 2,6-dimethoxylphenol).

Using the final equation in the kinetics derivation in the previous page, the rate constant for the reaction between $^1\mathbf{O_2}$ and phenols ($\boldsymbol{k_r}$) can be estimated as shown in Table S3.

Table S3. Rate constant $\boldsymbol{k_r}$ between $^1\mathbf{O_2}$ and phenols.

**Phenol**	**k_r ($M^{-1}s^{-1}$)**

2,6-dimethoxyphenol	$(5.0 \pm 0.7) \times 10^7$
Ac-Tyr-NHMe	$(5.9 \pm 0.7) \times 10^7$

CD45 labeling on Jurkat cells with CD45 using RFT primary/secondary antibody system

Jurkat NF-κB GFP cells (System Biosciences, Palo Alto, CA, TR850A-1) were cultured at 37°C and 5% CO_2 in 1x RPMI 1640 (Corning, 10-040-CV) supplemented with 10% FBS (Cytiva, SH30910.03) and 100 IU penicillin/100 µg ml^{-1} streptomycin (Corning, 30-002-CI) in vented cap, tissue culture flasks (Corning, 430639, 430641U, or 430825) as needed.

For mass spectrometry scale, RFT-based labeling of CD45, 20-30 million Jurkat NK-κB GFP cells were centrifuged at 800xg for 5 minutes at 4°C, resuspended in cold complete media at a concentration of 20 million cells/ml and transferred to 1.5ml Eppendorf Protein LoBind microcentrifuge tubes (Sigma-Aldrich, Z666505-100EA) in 1ml aliquots. 10µg of either Mouse IgG1, k isotype control antibody (BD, 556648) or mouse anti-human CD45 antibody (BD, 555480) were added to the corresponding samples and incubated on a rotisserie for 30 min at 4°C.

The cells were pelleted by centrifugation for 5 min at 800xg, 4°C, followed by 2x washes with 1ml cold 1x DPBS (Thermo Fisher, 14190144). 10µg of goat anti-mouse IgG-RFT conjugate (prepared as described previously),[11] were added to all samples and incubated on a rotisserie for 30 min at 4°C.

After the 30 min incubation, the samples were washed 2x with 1ml cold 1x DPBS. Each cell pellet was resuspended in 1ml of cold 1x DPBS containing 250 µM biotin tyramide (Iris Biotech, LS-3500.1000) and irradiated for 2 or 10 minutes in the biophotoreactor (BPR200, Efficiency Aggregators, Fisher, NC1558343) under 455 nm visible light at 100% intensity. After irradiation, the samples were washed 2x in 1ml cold 1x DBPS with pelleting for 5min at 800xg, 4°C in between washes. For untargeted labeling experiments, cells were resuspended in 1ml of cold 1x DPBS containing 50 µM RFT and 250 µM biotin tyramide or 250 µM biotin tyramide only followed by irradiation for 10 minutes in the biophotoreactor as indicated above.

Cells were permeabilized with 1ml of membrane permeabilization buffer (MEM-PER Plus Membrane Fractionation Kit, Thermo Fisher Scientific, 89842) containing 1x protease inhibitors (Sigma-Aldrich, 11873580001) and incubated for 20 min at 4°C. Samples were centrifuged for 15 min at 16,000xg and 4°C. The supernatant was removed, and the pellet was resuspended in 300µl lysis buffer (RIPA buffer, Thermo Fisher Scientific, 89900) containing 1% SDS and 1x protease inhibitors. Sonication was used to lyse the membrane pellet (1x 5s at power level 6 using a Sonic Dismembrator, Fisherbrand, Model 120) and then heated for 5 min at 95°C. The volume per sample was brought up to 1.3ml with RIPA Buffer and sonicated again (2x5s at power level 6). Protein concentration was measured using a bicinchoninic assay (BCA) kit (Pierce, 23227), and the samples were stored at -80°C until streptavidin bead enrichment.

For bead enrichment, the complete cell lysate of each sample was added to Protein LoBind microcentrifuge tubes containing 250µl of streptavidin magnetic beads (Thermo Fisher Scientific, 88817) pre-washed 2x with 1ml RIPA buffer (with bead pelleting with a magnetic rack in between wash steps). The samples were incubated on a rotisserie for end-over-end mixing for 3 hours at room temperature, followed by bead

pelleting on a magnetic rack. The supernatant was removed, and the beads were washed with 3x1ml 1%SDS in 1x DPBS, 3x1ml 1M NaCl in 1x DPBS, 3x1ml 10% EtOH in 1x DPBS, and 1x in 1ml RIPA Buffer (with 5 min washes in between pelleting steps). The beads were then pelleted on a magnetic rack, the buffer removed, and the dried beads resuspended in 30µl of 4x Laemmli sample buffer supplemented with 20mM DTT and 25mM biotin, followed by a 10 min incubation at 95°C. The samples were placed back on a magnetic rack to collect and transfer the elutions to new Protein LoBind microcentrifuge tubes. The eluted samples were sealed with parafilm and stored at -80°C pending proteomic analysis (performed at IQ Proteomics, Cambridge, MA).

CD45 labeling on Jurkat cells with CD45 using HRP primary/secondary antibody system

Jurkat NF-κB GFP cells (System Biosciences, Palo Alto, CA, TR850A-1) were cultured at 37°C and 5% CO_2 in 1x RPMI 1640 (Corning, 10-040-CV) supplemented with 10% FBS (Cytiva, SH30910.03) and 100 IU penicillin/100 µg ml^{-1} streptomycin (Corning, 30-002-CI) in vented cap, tissue culture flasks (Corning, 430639, 430641U, or 430825) as needed.

For mass spectrometry scale, HRP-based labeling of CD45, 20-30 million Jurkat NK-κB GFP cells were centrifuged at 800xg for 5 minutes at 4°C, resuspended in cold complete media at a concentration of 20 million cells/ml and transferred to 1.5ml Eppendorf Protein LoBind microcentrifuge tubes (Sigma-Aldrich, Z666505-100EA) in 1ml aliquots. 10µg of either Mouse IgG1, k isotype control antibody (BD, 556648) or mouse anti-human CD45 antibody (BD, 555480) were added to the corresponding samples and incubated on a rotisserie for 30 min at 4°C.

The cells were pelleted by centrifugation for 5 min at 800xg, 4°C, followed by 2x washes with 1ml cold 1x DPBS (Thermo Fisher, 14190144). 10µg of goat anti-mouse IgG-HRP (Millipore, AP124P) were added to all samples and incubated on a rotisserie for 30 min at 4°C.

After the 30 min incubation, the samples were washed 2x with 1ml cold 1x DPBS. Each cell pellet was resuspended in 1ml of cold 1x DPBS and transferred to 4ml of reaction buffer (250 µM biotin tyramide (Iris Biotech, LS-3500.1000) + 1 mM H_2O_2 (Sigma-Aldrich)) in 15ml conical tubes and incubated for 1 min on ice. The labeling reaction was immediately quenched with 5ml of quenching buffer (10mM sodium ascorbic acid + 10mM sodium azide + 5mM Trolox in 1x DPBS) and centrifuged for 5 min at 800xg, 4°C. The cell pellets were washed with 10ml of quenching buffer and centrifuged again as above. Samples were then resuspended in 1ml of cold 1x DPBS and transferred to new Protein LoBind microcentrifuge tubes, followed by 2x washes in 1ml cold 1x DBPS with pelleting for 5min at 800xg, 4°C in between washes.

Cells were permeabilized with 1ml of membrane permeabilization buffer (MEM-PER Plus Membrane Fractionation Kit, Thermo Fisher Scientific, 89842) containing 1x protease inhibitors (Sigma-Aldrich, 11873580001) and incubated for 20 min at 4°C. Samples were centrifuged for 15 min at 16,000xg and 4°C. The supernatant was removed, and the pellet was resuspended in 300µl lysis buffer (RIPA buffer, Thermo Fisher Scientific, 89900) containing 1% SDS and 1x protease inhibitors. Sonication was used to lyse the membrane pellet (1x 5s at power level 6 using a Sonic Dismembrator, Fisherbrand, Model 120) and then heated for 5 min at 95°C. The volume per sample was brought up to 1.3ml with RIPA Buffer and sonicated again (2x5s at power level 6). Protein concentration was measured using a bicinchoninic assay (BCA) kit (Pierce, 23227), and the samples were stored at -80°C until streptavidin bead enrichment.

For bead enrichment, the complete cell lysate of each sample was added to Protein LoBind microcentrifuge tubes containing 250µl of streptavidin magnetic beads (Thermo Fisher Scientific, 88817) pre-washed 2x with 1ml RIPA buffer (with bead pelleting with a magnetic rack in between wash steps). The samples were incubated on a rotisserie for end-over-end mixing for 3 hours at room temperature, followed by bead pelleting on a magnetic rack. The supernatant was removed, and the beads were washed with 3x1ml 1%SDS in 1x DPBS, 3x1ml 1M NaCl in 1x DPBS, 3x1ml 10% EtOH in 1x DPBS, and 1x in 1ml RIPA Buffer (with 5 min washes in between pelleting steps). The beads were then pelleted on a magnetic rack, the buffer removed, and the dried beads resuspended in 30µl of 4x Laemmli sample buffer supplemented with 20mM DTT and 25mM biotin, followed by a 10 min incubation at 95°C. The samples were placed back on a magnetic rack to collect and transfer the elutions to new Protein LoBind microcentrifuge tubes. The eluted samples were sealed with parafilm and stored at -80°C pending proteomic analysis (performed at IQ Proteomics, Cambridge, MA).

LC-MS/MS-based Proteomic Analysis of Labeled Cell Experiments
All mass spectra were acquired on an Orbitrap Fusion Lumos coupled to an EASY nanoLC-1000 (or nanoLC-1200) (Thermo Fisher) liquid chromatography system as described previously.[12]

Bioinformatic Analysis of LC-MS/MS Data
All bioinformatic analysis of LC-MS/MS data was performed in the R statistical computing environment as described previously.[12]

Volcano Plot Generation
Volcano plots were generated in R with the ggplot library as described previously.[12] Note that the log_2FC was set to 1.5 for all CD45 targeted labeling experiments.

STRING Plot Analysis
Proteins reaching statistical significance and the log_2FC cutoff defined in the experiment were analyzed with StringDB,[1] for associations between proteins to generate a protein interaction map. The following settings were used for the analysis: Network type: Physical subnetwork; Meaning of network edges: confidence; Active Interaction Sources: Textmining, Experiments, Databases boxes checked; Mean required interaction score: 0.400; Network display option: disable structure previews inside network bubbles. All other features were set to default. Edge thickness indicates strength of data evidence from the StringDB.

General Synthetic Information

Unless specified, all commercially reagents were used as received. Acetonitrile was purchased from EMD Chemicals Inc. (DriSolv, 25 ppm BHT) and used as received. Methanol, dichloromethane, ethyl acetate and heptane were purchased from Fisher Scientific and used as received. The flavin photocatalysts (alloxazine and lumichrome were purchased from Aldrich (**A28651** and **103217),** lumiflavin was purchased from Santa Cruz Biotechnology and riboflavin was purchased from Fisher) and used as received. Other photocatalysts (Tris(2,2′-bipyridyl)dichlororuthenium(II) hexahydrate, Tris[2-phenylpyridinato-C^2,*N*]iridium(III) and Eosin Y) were purchased from Aldrich and used as received.

All NMR spectra were collected on either a Bruker 400 Avance III with a 5 mm BBFO probe (400 MHz for ^1H; 101 MHz for ^{13}C) or a Bruker 500 Avance III HD with a 5 mm BBO Nitrogen cryoprobe (500 MHz for ^1H; 126 MHz for ^{13}C). The proton signal for non-deuterated solvent (d 7.27 for $CHCl_3$, δ 2.50 for DMSO) was used as an internal reference for 1H NMR spectra. For ^{13}C NMR spectra, chemical shifts are

reported relative to the δ 77.00 resonance of $CDCl_3$ or δ 39.52 resonance of DMSO-*d6*. Deuterated solvents ($CDCl_3$ and DMSO-*d6*) were purchased from Cambridge Isotope Laboratories Inc. and used as received.

HPLC analyses were performed on an Agilent 1260 Infinity II LC system using a 100 mm Agilent Zorbax 300SB-C18 3.5 µm analytical column. Peptide purification performed using semi-preparative HPLC on an Agilent 1260 Infinity II LC system using a Vydac C-18 218TP510 semi-preparative column. Solvent removal was performed using a Benchtop Pro evaporator.

Low-Resolution Mass Spectrometry analyses were conducted on an Agilent 1290 Infinity II LC system (1290 Infinity II multisampler, 1290 Infinity II Binary Pump) with Agilent 6130 Single Quadruple MS and Supelco Ascentis Express C18 column (2.1 mm x 50 mm, 2.7 µm); Column Temperature 50 °C; 0.1% formic acid in water (v/v) as the mobile phase A; 0.1% formic acid in acetonitrile (v/v) as the mobile phase B; 1 mL/min as the flow; ESI+/-, 100-1000 m/z scan, 0.34 sec scan time as the MS method.

High-Resolution Mass Spectrometry analyses were conducted on an Agilent 6545 QTOF mass spectrometer (Agilent Technologies, Santa Clara, CA) in positive or negative electrospray mode. The system was calibrated to greater than 1 ppm accuracy across the mass range prior to analyses according to manufacturer's specifications. The samples were separated using UHPLC on an Agilent 1290 (Agilent Technologies, Santa Clara, CA) system prior to mass spectrometric analysis. The resulting spectra were automatically lockmass corrected and the target mass ions and any confirming adducts (Na+, NH4+) were extracted and combined as a chromatogram. The mass accuracy was calculated for all observed isotopes against the theoretical mass ions derived from the chemical formula using MassHunter software (Agilent Technologies, Santa Clara, CA).

Analytical thin layer chromatography (TLC) was performed on 60 F_{254} glass plates precoated with a 0.25-mm thickness of silica gel purchased from EMD chemical Inc. and TLC plates were visualized with UV light. Column chromatography was performed on TELEDYNE ISCO devices; *CombiFlash® Rf+* version: 2.0.4.

Photoredox Mediated C-H Functionalization of Peptides

Reaction optimization for C-H functionalization of peptide was achieved by using capped tyrosine and 2-methoxy-4-methylphenol, as depicted in Table S4 below. A summary of optimization results includes the use of riboflavin tetraacetate photocatalyst, as well as some blank/test reactions to test the limits of the transformation and its proposed mechanism.

Table S4. Optimization on the C-H Functionalization of Phenol Cross-Coupling

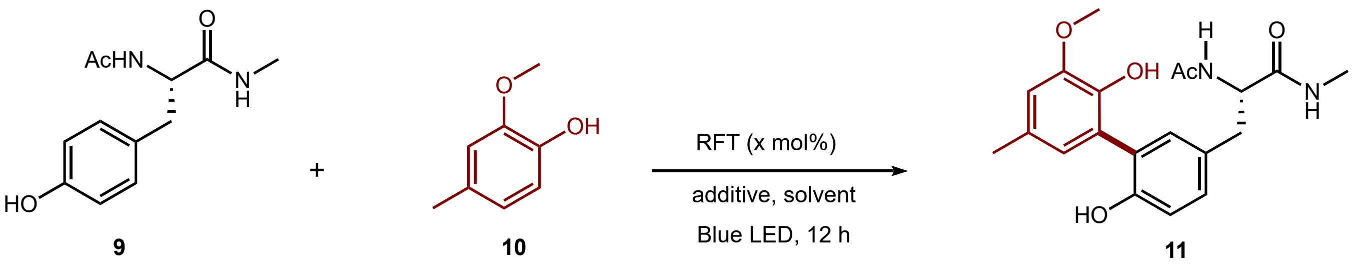

Entry[a]	Solvent	RFT (mol%)	additive	9 (equiv.)	12a (equiv.)	Conv. (%)[b]
1	DMSO	10	-	1	1	10%
2	MeCN	10	-	1	1	15%
3	DMF	10	-	1	1	<5%
4	H_2O	10	-	1	1	22%
5	MeOH	10	-	1	1	20%
6	MeOH/ H_2O (4:1)	10	-	1	1	32%
7	MeCN/ H_2O (4:1)	10	-	1	1	26%
8	MeCN/ H_2O (2:1)	10	-	1	1	37%
9	MeCN/ H_2O (1:1)	10	-	1	1	42%
10	pH 7 PBS buffer	10	-	1	1	43%
11[c]	MeCN/ H_2O (1:1)	10	-	1	1	57%
12[c]	pH 7 PBS buffer	10	-	1	1	51%
13[f]	MeCN/ H_2O (1:1)	10	$(NH_4)_2S_2O_8$ (10 mol%)	1	1	<5%
14[f]	MeCN/ H_2O (1:1)	10	HCO_2Na (10 mol%)	1	1	10%
15	MeCN/ H_2O (1:1)	10	TBAHS (10 mol%)	1	1	64%
16[c]	MeCN/ H_2O (1:1)	10	TBAHS (10 mol%)	1	1	68%
17[c]	MeCN/ H_2O (1:1)	10	TBAHS (10 mol%)	1	5	44%
18[c]	MeCN/ H_2O (1:1)	10	TBAHS (10 mol%)	1	2	73%
19[c]	MeCN/ H_2O (1:1)	10	TBAHS (10 mol%)	1	1.5	73%
20[c,f]	MeCN/ H_2O (1:1)	15	TBAHS (10 mol%)	1	1	47%
21[c,f]	MeCN/ H_2O (1:1)	30	TBAHS (10 mol%)	1	1	43%
22[c]	MeCN/ H_2O (1:1)	5	TBAHS (10 mol%)	1	1.5	80%
23[c]	MeCN/ H_2O (1:1)	5	TBAHS (10 mol%)	3	1	55%

24[c]	MeCN/ H_2O (1:1)	5	TBAHS (20 mol%)	1	1.5	30%
25[c]	MeCN/ H_2O (1:1)	5	TBAHS (50 mol%)	1	1.5	<10%
26[c,d]	MeCN/ H_2O (1:1)	5	TBAHS (10 mol%)	1	1.5	0%
25[c]	MeCN/ H_2O (1:1)	0	TBAHS (10 mol%)	1	1.5	0%
26[e]	MeCN/ H_2O (1:1)	5	TBAHS (10 mol%)	1	1.5	10%

[a]All reactions were performed on a 0.5 mmol scale, [b]Conversion calculated from UPLC areas analysis, [c]Pre-oxygenated solvent (bubbled oxygen in combined solvent for 5 min) was used, [d]Experiment was conducted in the dark, [e]Reaction was degassed by freeze-thaw method and purged with N_2. [f]Several side products identified by LC-MS. Tetrabutylammonium hydrogen-sulfate (TBAHS)

General Procedure for the Phenol C-H Functionalization

Riboflavin tetratacetate (RFT) (5 mol%), tyrosine containing peptide or phenol containing small molecule (1.0 equiv), coupling partner phenol (1.5 equiv) and tetrabutylammonium hydrogen sulfate (TBAHS) were added to a vial containing a stir-bar. The vial was sealed with a screw cap with hole with a PTFE/silicone septum and pre-oxygenated 1:1 MeCN/water (bubbled oxygen in combined solvent for 5 min) (0.5 M). The reaction was stirred at room temperature for 12 h under blue LED illumination. The reaction was evaporated to dryness and directly purified by HPLC using buffers A (water + 0.1% TFA) and B (9:1 acetonitrile:water + 0.1% TFA).

(*S*)-2-acetamido-3-(2',6-dihydroxy-3'-methoxy-5'-methyl-[1,1'-biphenyl]-3-yl)-N-methylpropanamide:

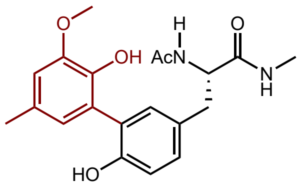

11, 53% yield

^{1}H NMR (400 MHz, DMSO-*d*$_6$) δ 9.00 (s, 1H), 8.16 (s, 1H), 7.95 (dd, *J* = 7.4, 6.4 Hz, 2H), 6.97 (d, *J* = 6.4 Hz, 2H), 6.75 (d, *J* = 7.4 Hz, 2H), 6.51 (s, 1H), 4.38 – 4.26 (m, 1H), 3.79 (s, 3H), 2.85 (dd, *J* = 13.8, 5.0 Hz, 1H), 2.63 (dd, *J* = 13.7, 9.4 Hz, 1H), 2.55 (d, *J* = 4.5 Hz, 3H), 2.23 (s, 2H), 1.78 (s, 3H).
^{13}C NMR (101 MHz, DMSO) δ 171.87, 169.19, 152.78, 147.62, 141.20, 132.07, 128.72, 128.33, 127.38, 126.21, 125.59, 123.50, 115.50, 111.48, 55.83, 54.60, 37.09, 25.59, 22.61, 20.75.
HRMS (ESI), m/z: calculated for $C_{20}H_{24}N_2O_5$ [M+H]$^+$: 373.1758, found: 373.1770.

(S)-2-acetamido-3-(4-hydroxy-3-(2-methoxy-4-methylphenoxy)phenyl)-N-methylpropanamide (12) was isolated alongside compound 11:

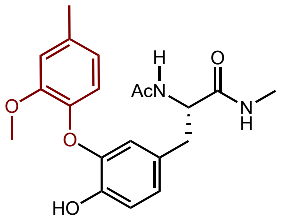

12, 5% yield

^{1}H NMR (400 MHz, DMSO-*d*$_6$) δ 8.50 (s, 1H), 8.08 (d, *J* = 8.4 Hz, 1H), 7.88 (d, *J* = 4.7 Hz, 1H), 7.12 (d, *J* = 8.6 Hz, 2H), 6.73 (d, *J* = 8.6 Hz, 2H), 6.63 (d, *J* = 1.7 Hz, 1H), 6.37 – 6.30 (m, 1H), 4.35 (td, *J* = 9.3, 5.0 Hz, 1H), 3.78 (s, 3H), 2.89 (dd, *J* = 13.8, 5.0 Hz, 1H), 2.66 (dd, *J* = 13.8, 9.5 Hz, 1H), 2.55 (d, *J* = 4.6 Hz, 3H), 2.18 (s, 3H), 1.76 (s, 3H).
^{13}C NMR (101 MHz, DMSO) δ 171.62, 169.06, 156.39, 148.98, 142.87, 136.18, 131.58, 130.07, 127.73, 116.00, 113.97, 109.10, 55.90, 54.25, 36.96, 25.52, 22.53, 20.67.
HRMS (ESI), m/z: calculated for $C_{20}H_{24}N_2O_5$ [M+H]$^+$: 373.1758, found: 373.1771.

(*S*)-2-acetamido-N-((*S*)-1-amino-3-(4',6-dihydroxy-3',5'-dimethoxy-[1,1'-biphenyl]-3-yl)-1-oxopropan-2-yl)-3-phenylpropanamide (13):

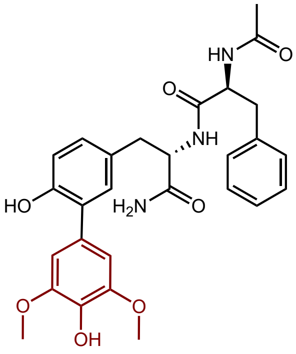

13, 37% yield

^{1}H NMR (400 MHz, DMSO-d_6) δ 8.18 (s, 1H), 8.05 (d, *J* = 8.3 Hz, 1H), 7.97 (d, *J* = 8.3 Hz, 1H), 7.28 (s, 1H), 7.26 – 7.21 (m, 2H), 7.21 – 7.15 (m, 5H), 7.09 (s, 1H), 6.81 (d, *J* = 8.6 Hz, 2H), 6.31 (s, 2H), 4.41 (m, 3H), 3.67 (s, 6H), 2.95 (m, 3H), 2.77 (dd, *J* = 13.8, 9.0 Hz, 1H), 2.67 (dd, *J* = 13.8, 10.0 Hz, 1H), 1.71 (s, 3H).
^{13}C NMR (101 MHz, DMSO) δ 172.68, 171.07, 169.16, 156.64, 148.54, 147.72, 138.00, 132.01, 130.52, 129.08, 128.00, 126.19, 116.57, 97.95, 55.98, 54.04, 37.26, 36.71, 22.42.
HRMS (ESI), m/z: calculated for $C_{28}H_{31}N_3O_7$ $[M+H]^+$: 522.2235, found: 522.2247.

(S)-2-amino-3-(5'-((S)-3-amino-2-(2-(((benzyloxy)carbonyl)amino)acetamido)-3-oxopropyl)-2',6-dihydroxy-5-methoxy-[1,1'-biphenyl]-3-yl)propanoic acid (14):

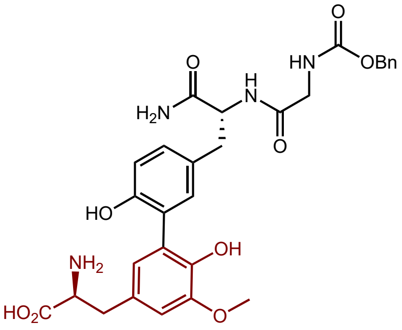

14, 26% yield

^{1}H NMR (400 MHz, DMSO-d_6) δ 8.92 (s, 1H), 8.42 (s, 1H), 8.20 (s, 2H), 7.97 (d, *J* = 8.3 Hz, 1H), 7.36 (m, 6H), 7.09 (s, 1H), 7.01 (d, *J* = 8.6 Hz, 2H), 6.85 (d, *J* = 1.8 Hz, 1H), 6.77 (d, *J* = 8.0 Hz, 1H), 6.65 (s, 1H), 5.01 (s, 2H), 4.39 – 4.32 (m, 1H), 4.19 (s, 1H), 3.82 (s, 3H), 3.68 (dd, *J* = 16.7, 6.2 Hz, 1H), 3.56 (dd, *J* = 16.7, 6.2 Hz, 1H), 3.04 (ddt, *J* = 20.8, 14.2, 6.2 Hz, 2H), 2.93 (m, 1H), 2.71 (dd, *J* = 14.1, 8.9 Hz, 1H).
^{13}C NMR (101 MHz, DMSO) δ 173.07, 170.63, 168.84, 156.53, 152.82, 147.83, 142.97, 137.00, 132.03, 128.99, 128.37, 128.07, 127.83, 127.74, 126.10, 124.99, 124.54, 124.32, 115.52, 111.85, 65.50, 55.75, 54.13, 53.37, 43.43, 36.85, 35.50.
HRMS (ESI), m/z: calculated for $C_{29}H_{32}N_4O_9$ $[M+H]^+$: 581.2242, found: 581.2246.

(4*S*,7*S*,13*S*)-13-(((*S*)-1-amino-1-oxo-3-phenylpropan-2-yl)carbamoyl)-4-((*S*)-sec-butyl)-7-((4',6-dihydroxy-3',5'-dimethoxy-[1,1'-biphenyl]-3-yl)methyl)-2,5,8,11-tetraoxo-3,6,9,12-tetraazahexadecan-16-oic acid (15):

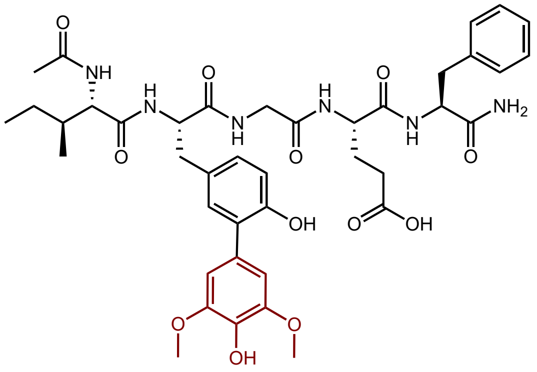

15, 44% yield

^{1}H NMR (400 MHz, DMSO-d_6) δ 9.18 (s, 1H), 8.17 (t, *J* = 5.5 Hz, 1H), 8.02 (d, *J* = 7.8 Hz, 1H), 7.94 (dd, *J* = 7.8, 3.3 Hz, 2H), 7.78 (d, *J* = 8.5 Hz, 1H), 7.33 (s, 1H), 7.23 (d, *J* = 6.4 Hz, 4H), 7.19 – 7.13 (m, 2H), 7.09 (s, 1H), 6.96 (dd, *J* = 8.2, 2.0 Hz, 1H), 6.80 (s, 2H), 6.76 (d, *J* = 8.2 Hz, 1H), 4.52 – 4.45 (m, 1H), 4.40 (td, *J* = 8.9, 5.0 Hz, 1H), 4.27 – 4.16 (m, 1H), 4.09 (t, *J* = 8.0 Hz, 1H), 3.77 (s, 6H), 3.71 (dd, *J* = 9.3, 5.6 Hz, 2H), 3.00 (m, 2H), 2.78 (m, 2H), 2.15 (t, *J* = 8.0 Hz, 2H), 1.81 (s, 4H), 1.73 – 1.56 (m, 2H), 1.33 – 1.20 (m, 1H), 1.04 – 0.91 (m, 1H), 0.71 (dd, *J* = 12.4, 7.0 Hz, 6H).
^{13}C NMR (101 MHz, DMSO) δ 173.99, 172.77, 171.73, 171.30, 170.71, 169.48, 168.83, 152.61, 147.47, 137.89, 134.61, 130.91, 129.15, 128.79, 128.30, 128.06, 127.54, 126.26, 115.71, 107.07, 57.20, 56.07, 54.37, 53.86, 52.06, 42.14, 37.36, 36.33, 29.96, 27.30, 24.21, 22.49, 15.24, 10.93.
HRMS (ESI), m/z: calculated for $C_{41}H_{52}N_6O_{12}$ $[M+H]^+$: 821.3716, found: 821.3725.

(*S*)-2-acetamido-3-(4',6-dihydroxy-3',5'-dimethoxy-[1,1'-biphenyl]-3-yl)-*N*-methylpropanamide (16):

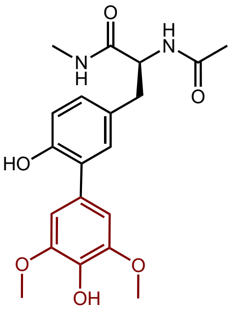

16, 40% yield

^{1}H NMR (400 MHz, DMSO-d_6) δ 9.18 (s, 1H), 8.10 (d, *J* = 8.5 Hz, 1H), 7.93 – 7.86 (m, 1H), 7.13 (d, *J* = 2.1 Hz, 1H), 6.92 (dd, *J* = 8.2, 2.1 Hz, 1H), 6.79 (s, 2H), 6.77 (d, *J* = 8.2 Hz, 1H), 4.36 (td, *J* = 9.2, 4.9 Hz, 1H), 3.77 (s, 6H), 2.87 (dd, *J* = 13.7, 4.8 Hz, 1H), 2.64 (dd, *J* = 13.7, 9.7 Hz, 1H), 2.57 (d, *J* = 4.6 Hz, 3H), 1.78 (s, 3H).
^{13}C NMR (101 MHz, DMSO) δ 171.81, 168.96, 152.49, 134.60, 130.84, 128.72, 128.26, 127.42, 115.72, 106.95, 56.02, 54.33, 37.06, 25.50, 22.53.
HRMS (ESI), m/z: calculated for $C_{20}H_{24}N_2O_6$ $[M+H]^+$: 389.1707, found: 389.1717.

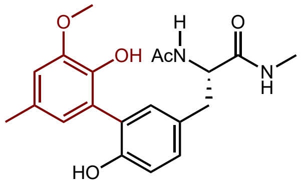

11

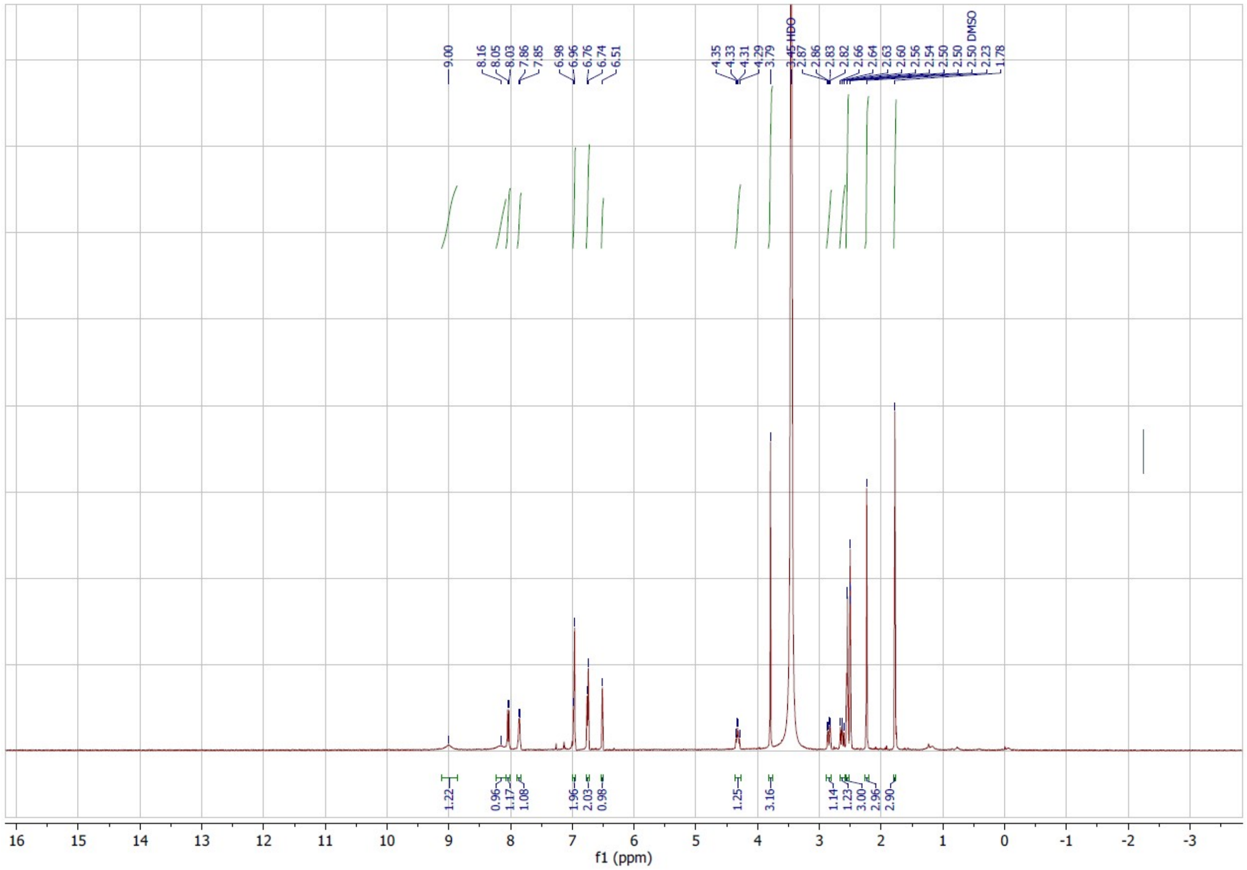
9.00
8.16
8.05
8.03
7.86
7.85
6.98
6.96
6.76
6.74
6.51
4.35
4.33
4.31
4.29
3.79
3.45 HDO
2.87
2.86
2.83
2.82
2.66
2.64
2.63
2.60
2.56
2.54
2.50
2.50
2.50 DMSO
2.23
1.78
1.22
0.96
1.17
1.08
1.96
2.03
0.98
1.25
3.16
1.14
1.23
3.00
2.96
2.90
f1 (ppm)

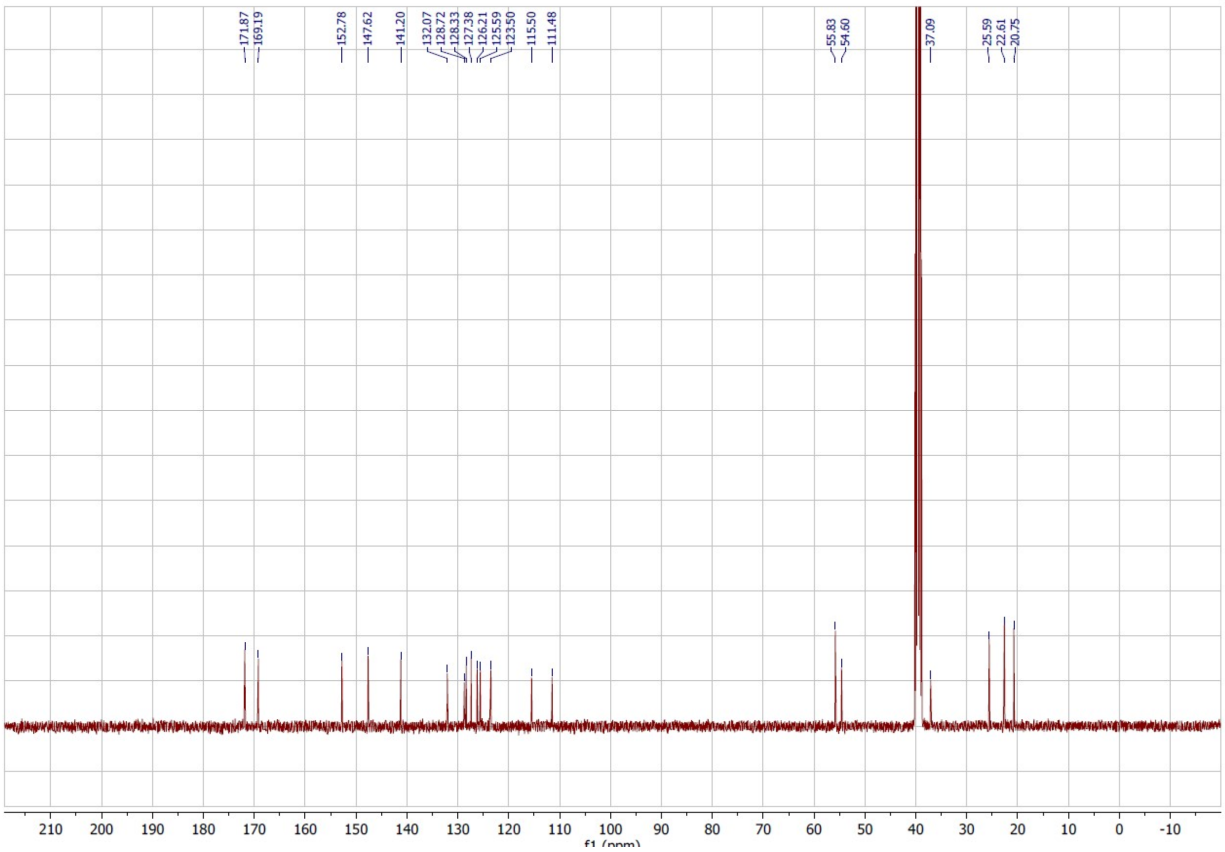

171.87
169.19
152.78
147.62
141.20
132.07
128.72
128.33
127.38
126.21
125.59
123.50
115.50
111.48
55.83
54.60
37.09
25.59
22.61
20.75
210 200 190 180 170 160 150 140 130 120 110 100 90 80 70 60 50 40 30 20 10 0 -10

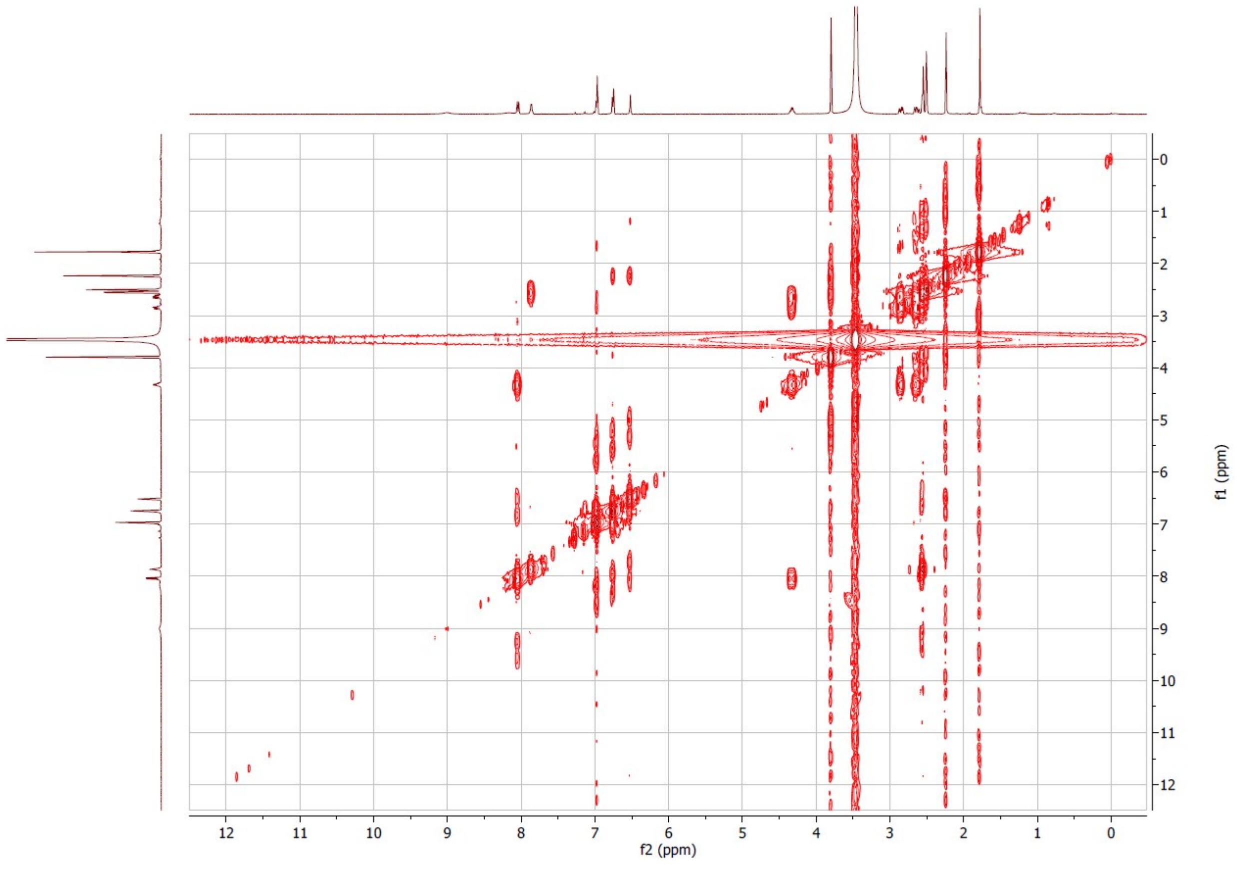

12 11 10 9 8 7 6 5 4 3 2 1 0
f2 (ppm)
0 1 2 3 4 5 6 7 8 9 10 11 12
f1 (ppm)

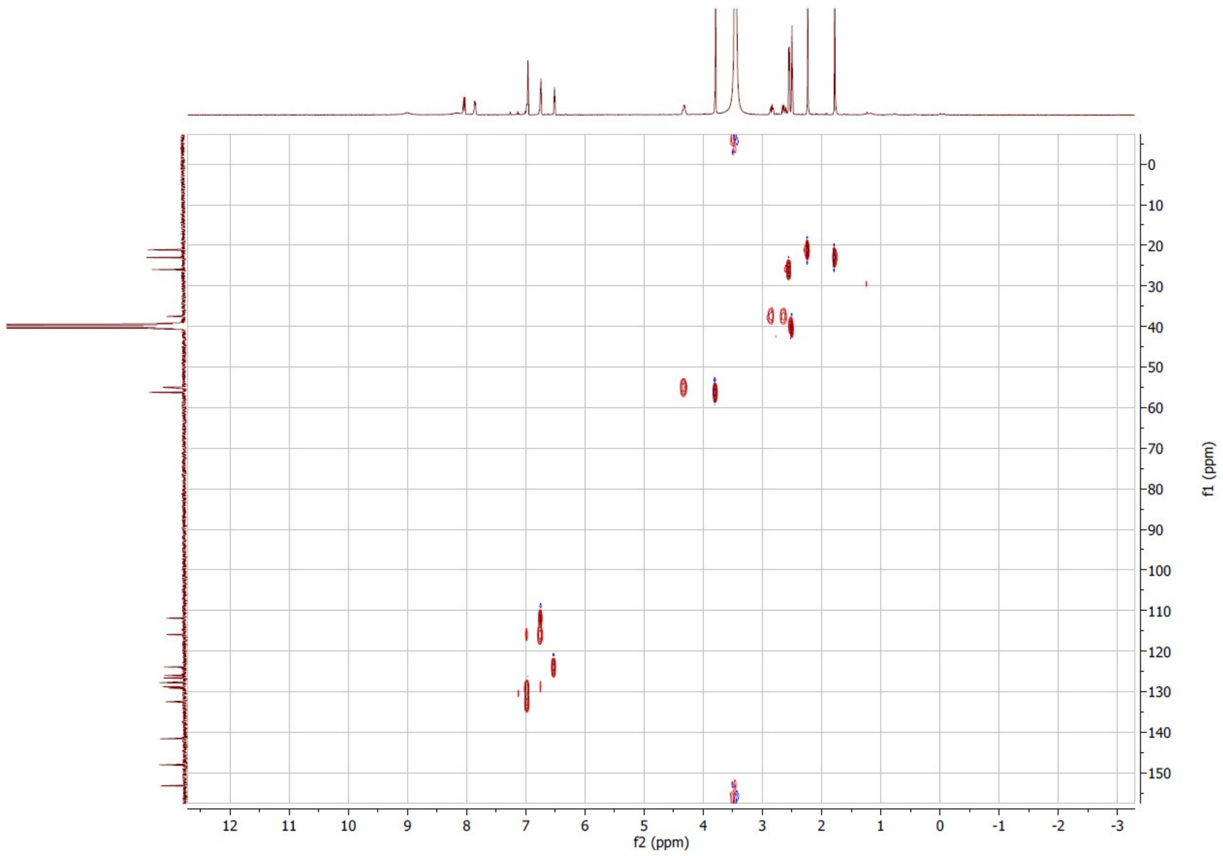
f2 (ppm)
f1 (ppm)

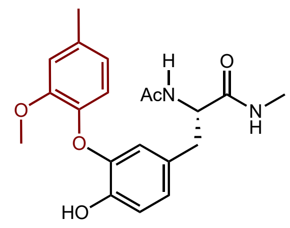
AcN
H
O
N
H
O
O
HO

12

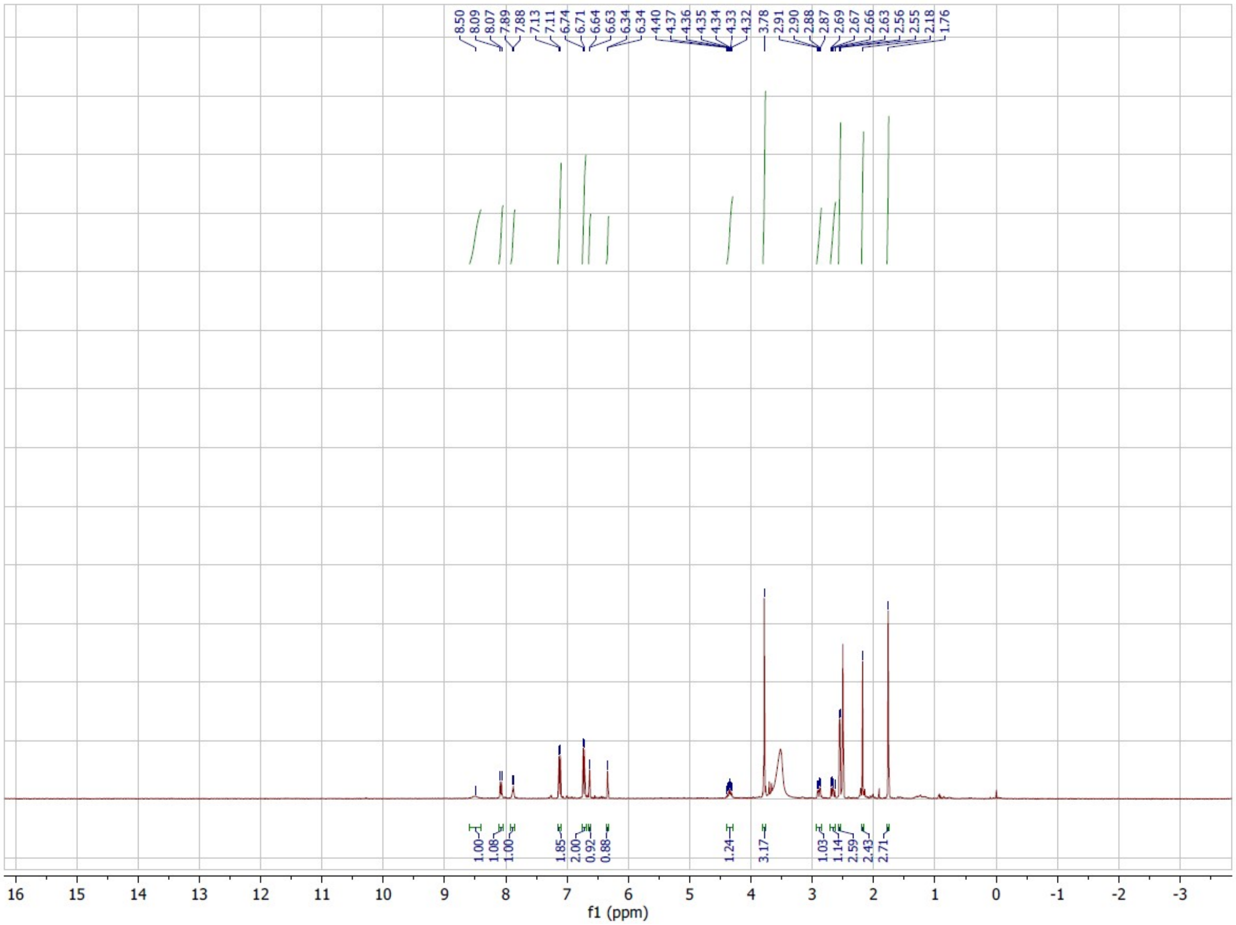
8.50
8.09
8.07
7.89
7.88
7.13
7.11
6.74
6.71
6.64
6.63
6.34
6.34
4.40
4.37
4.36
4.35
4.34
4.33
4.32
3.78
2.91
2.90
2.88
2.87
2.69
2.67
2.66
2.63
2.56
2.55
2.18
1.76
1.00
1.08
1.00
1.85
2.00
0.92
0.88
1.24
3.17
1.03
1.14
2.59
2.43
2.71
16 15 14 13 12 11 10 9 8 7 6 5 4 3 2 1 0 -1 -2 -3
f1 (ppm)

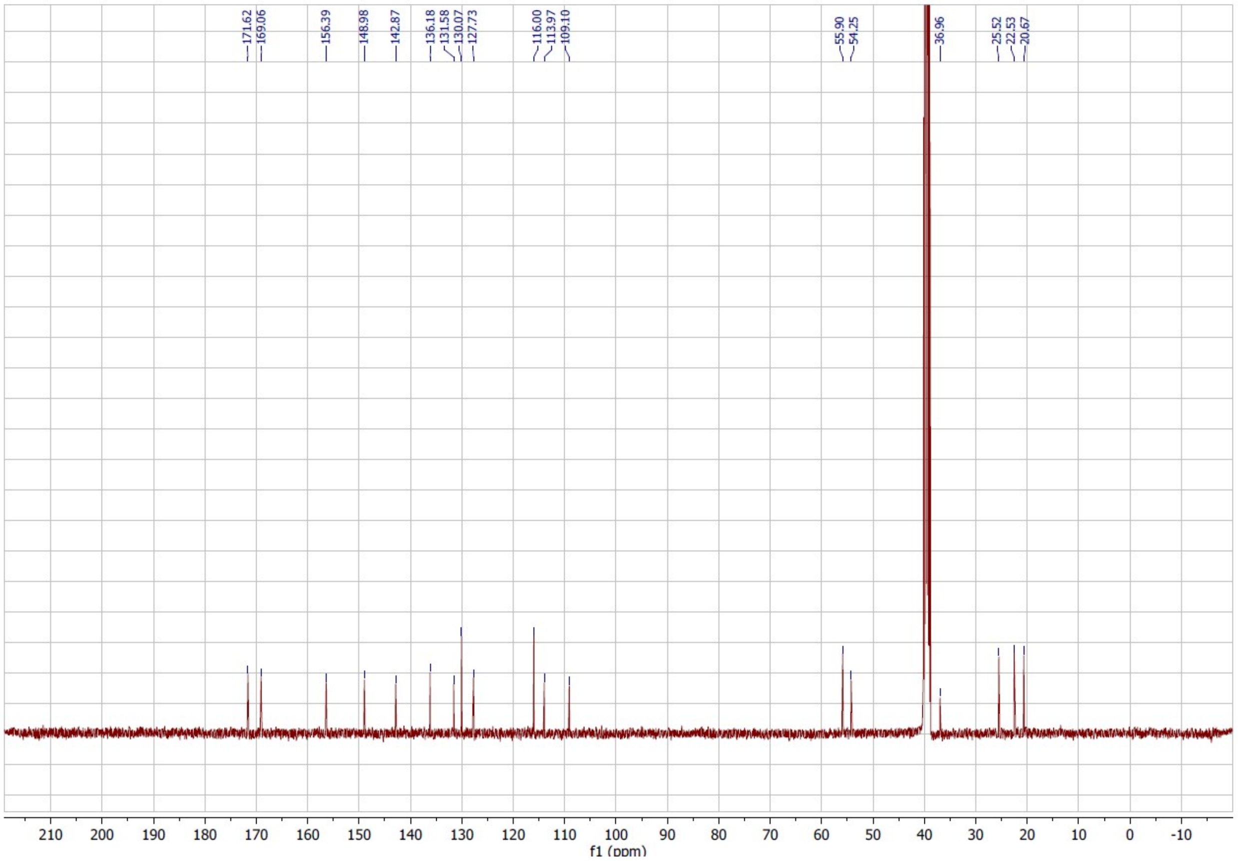
171.62
169.06
156.39
148.98
142.87
136.18
131.58
130.07
127.73
116.00
113.97
109.10
55.90
54.25
36.96
25.52
22.53
20.67
f1 (ppm)

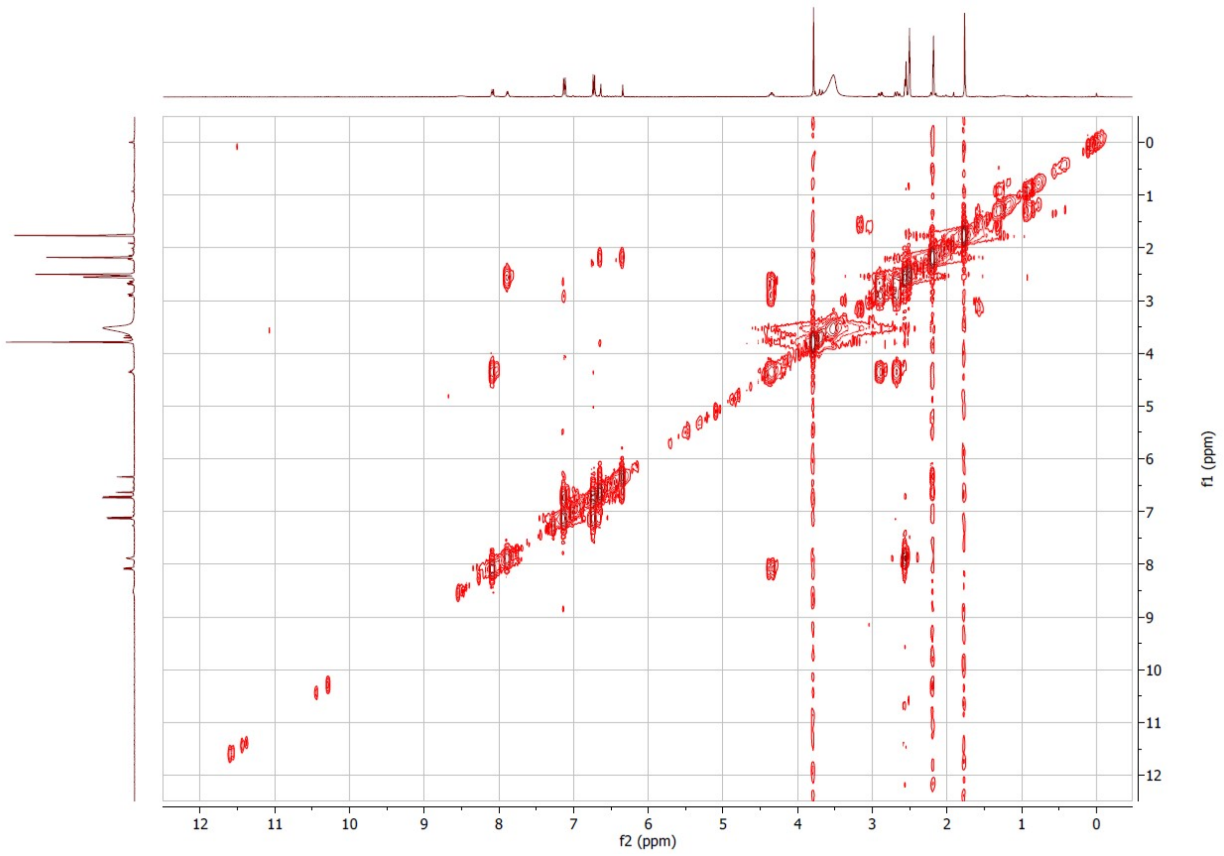
f2 (ppm)
f1 (ppm)

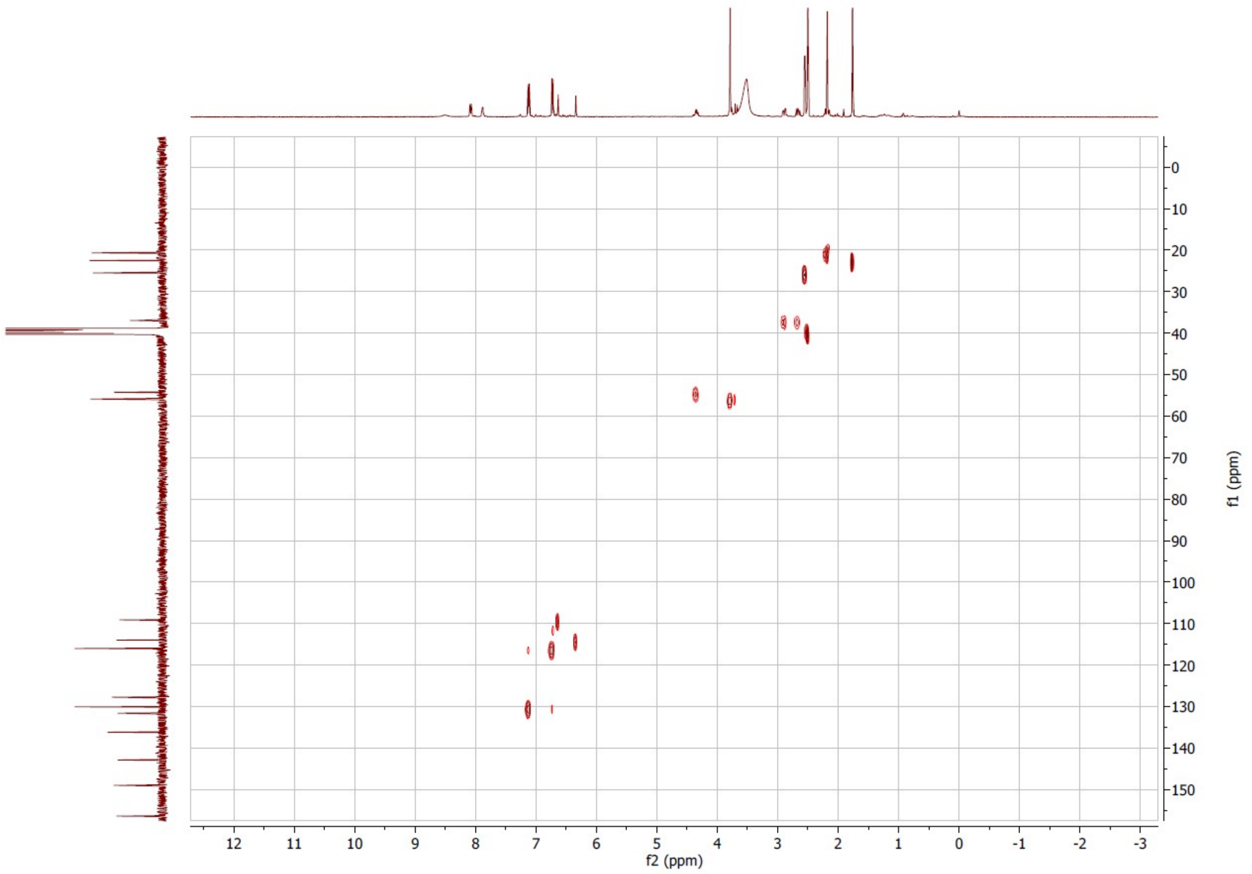
f2 (ppm)
f1 (ppm)

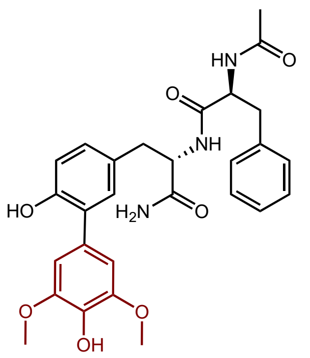

13

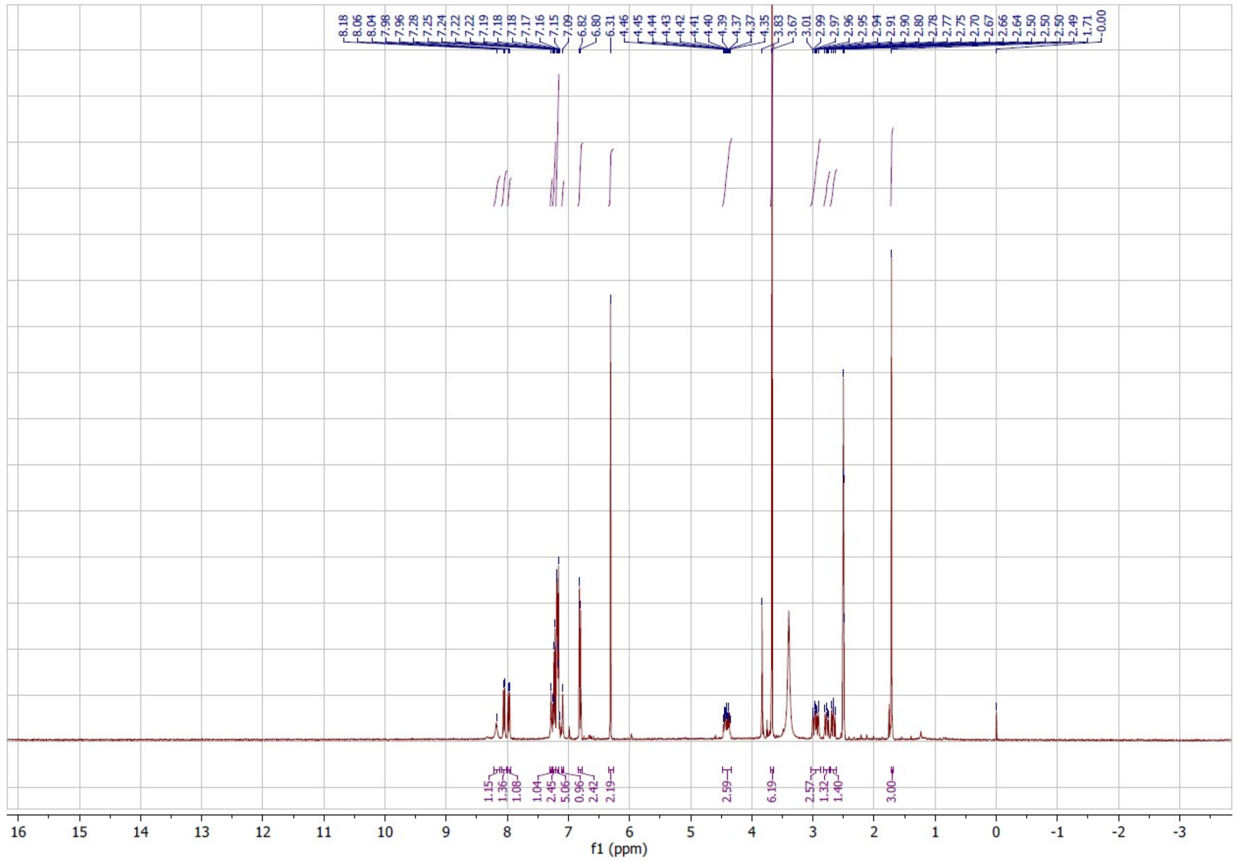

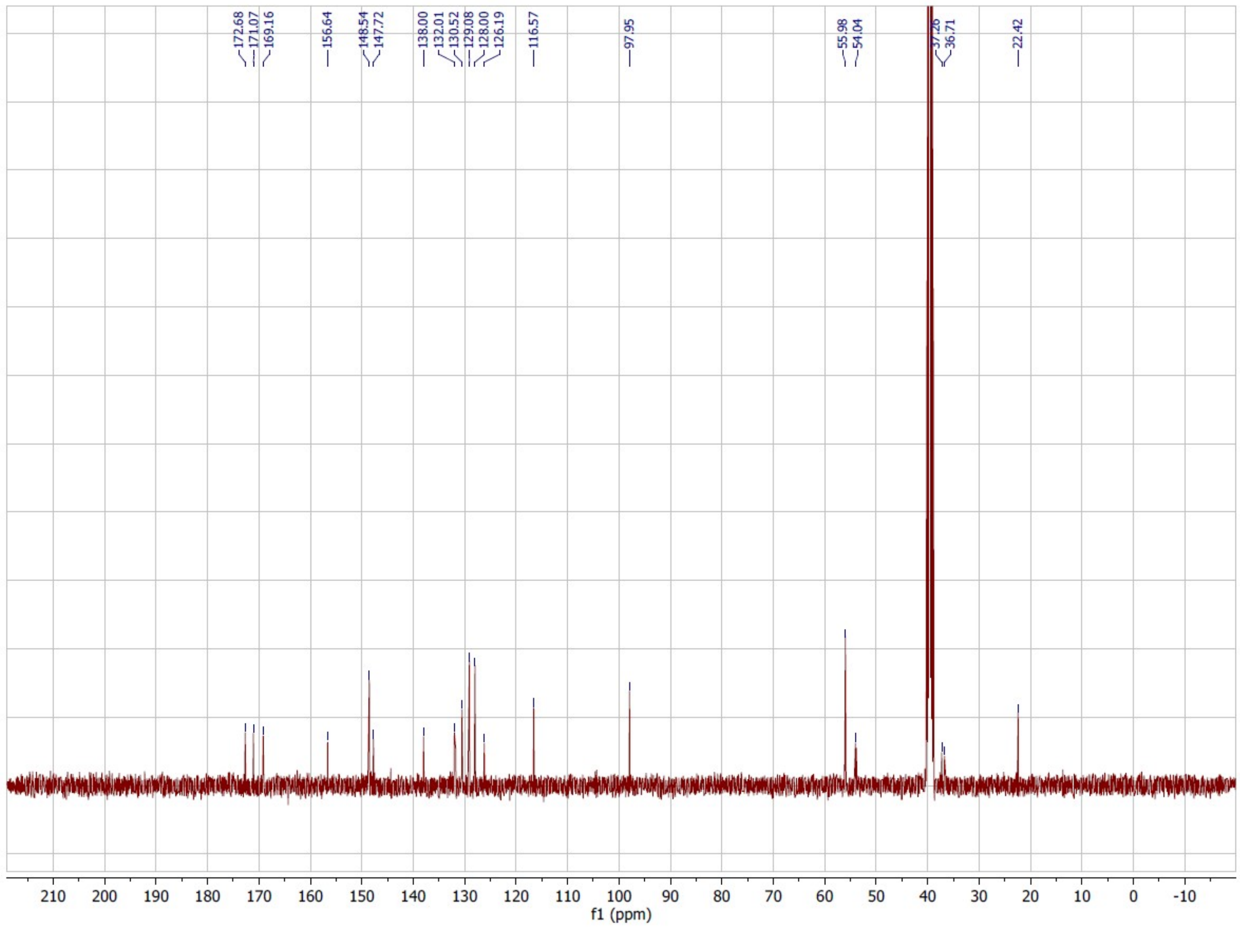
172.68
171.07
169.16
156.64
148.54
147.72
138.00
132.01
130.52
129.08
128.00
126.19
116.57
97.95
55.98
54.04
37.26
36.71
22.42
210 200 190 180 170 160 150 140 130 120 110 100 90 80 70 60 50 40 30 20 10 0 -10
f1 (ppm)

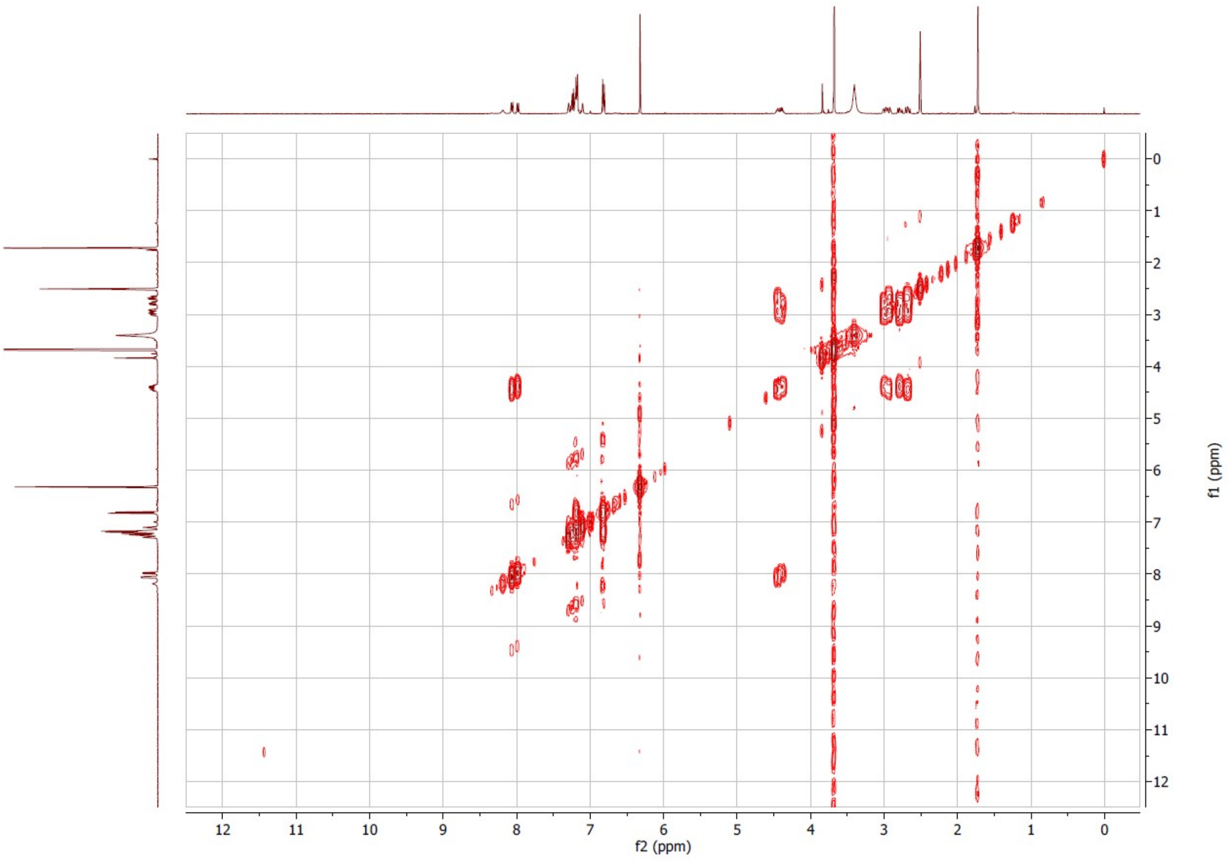
f2 (ppm)
f1 (ppm)

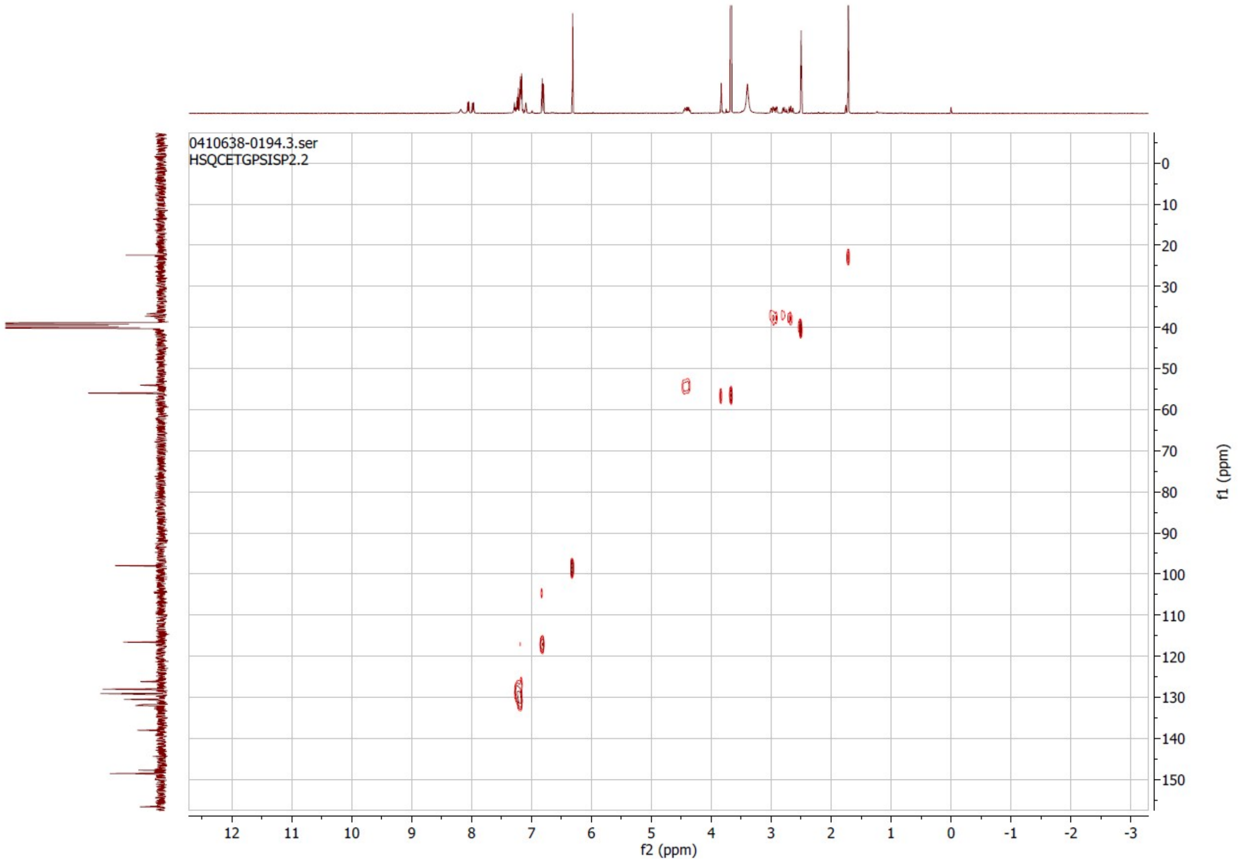
0410638-0194.3.ser
HSQCETGPSISP2.2
f2 (ppm)
f1 (ppm)

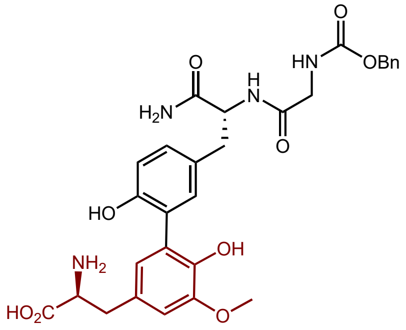

14

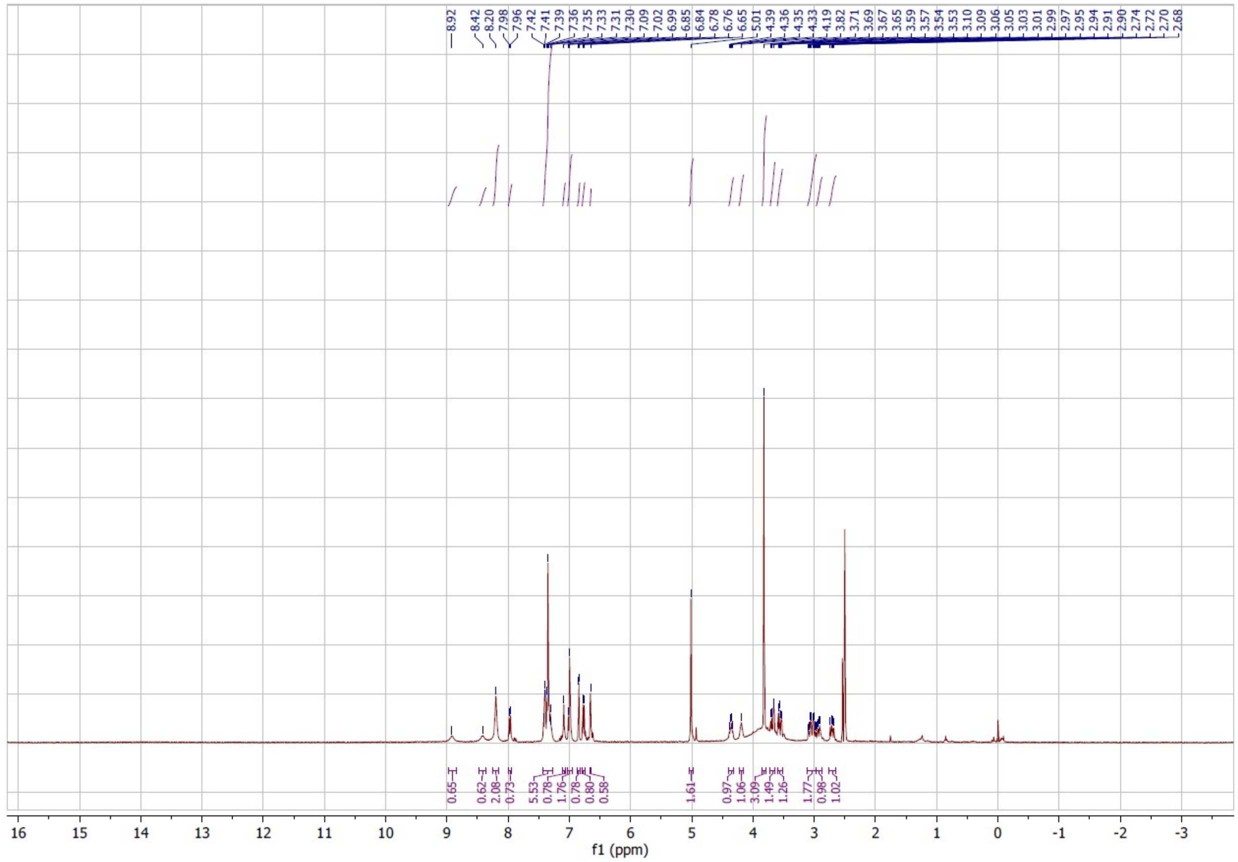

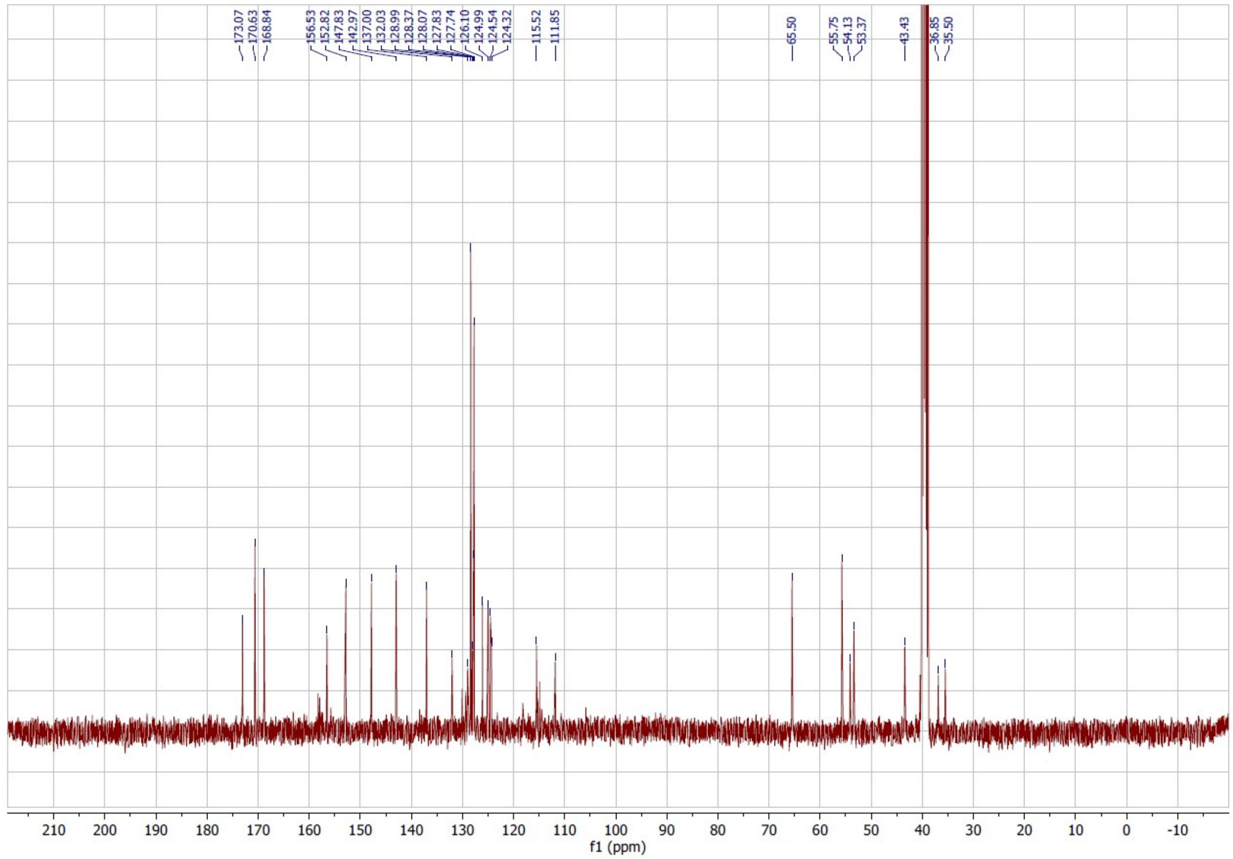
173.07
170.63
168.84
156.53
152.82
147.83
142.97
137.00
132.03
128.99
128.37
128.07
127.83
127.74
126.10
124.99
124.54
124.32
115.52
111.85
65.50
55.75
54.13
53.37
43.43
36.85
35.50
f1 (ppm)

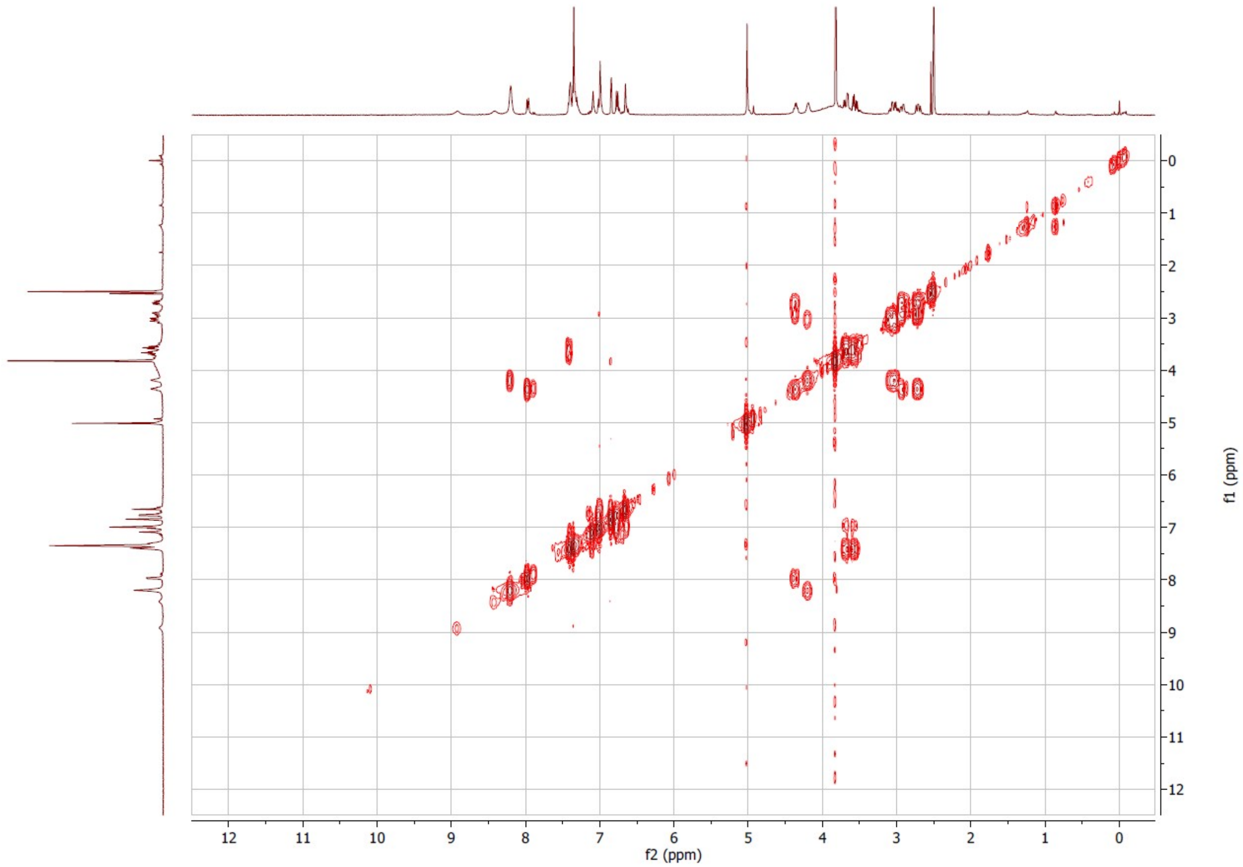
f2 (ppm)
f1 (ppm)

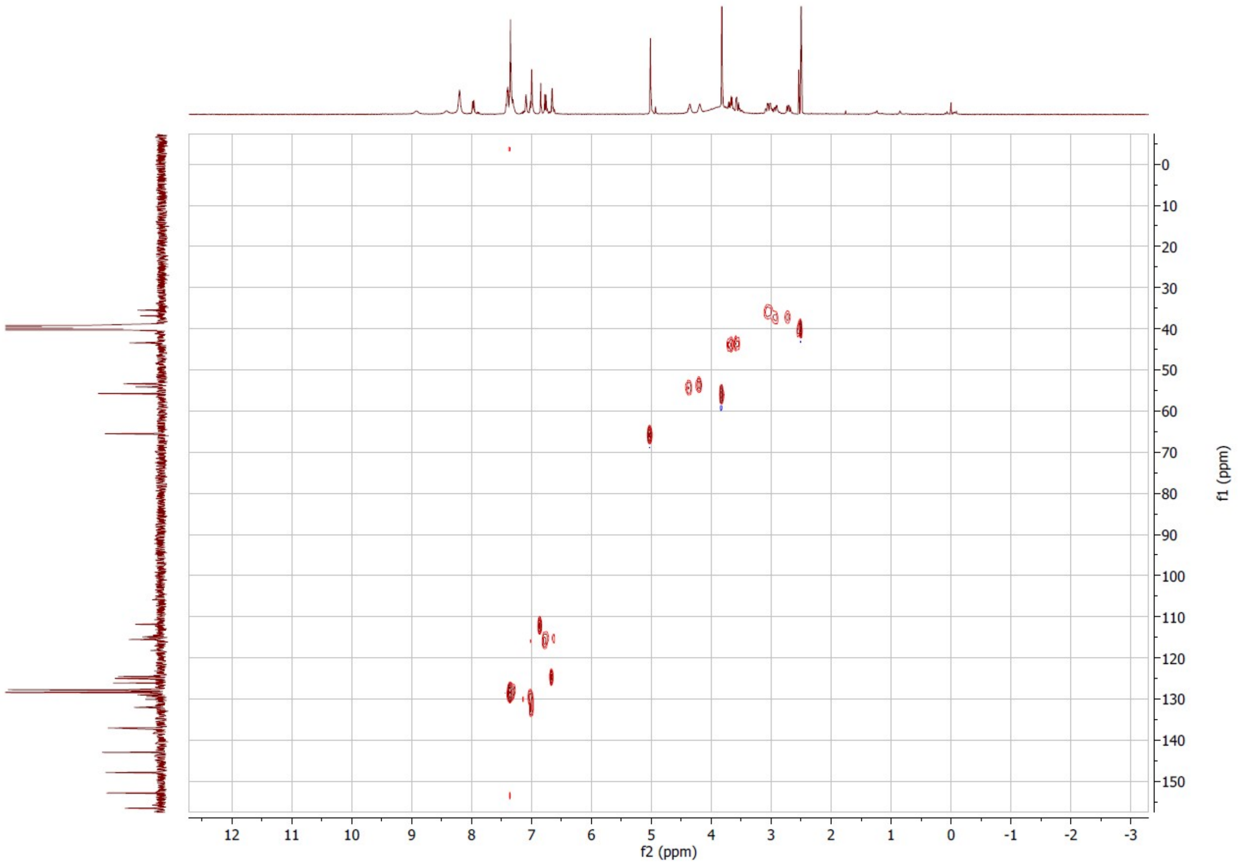
f2 (ppm)
f1 (ppm)

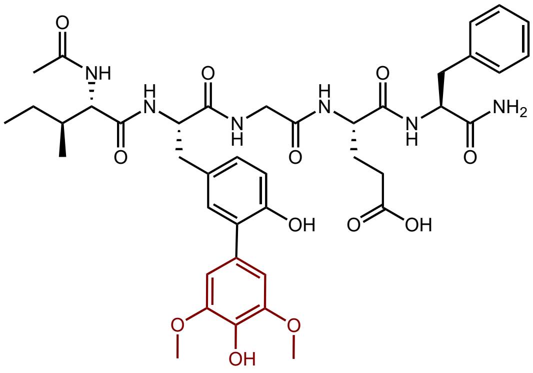

15

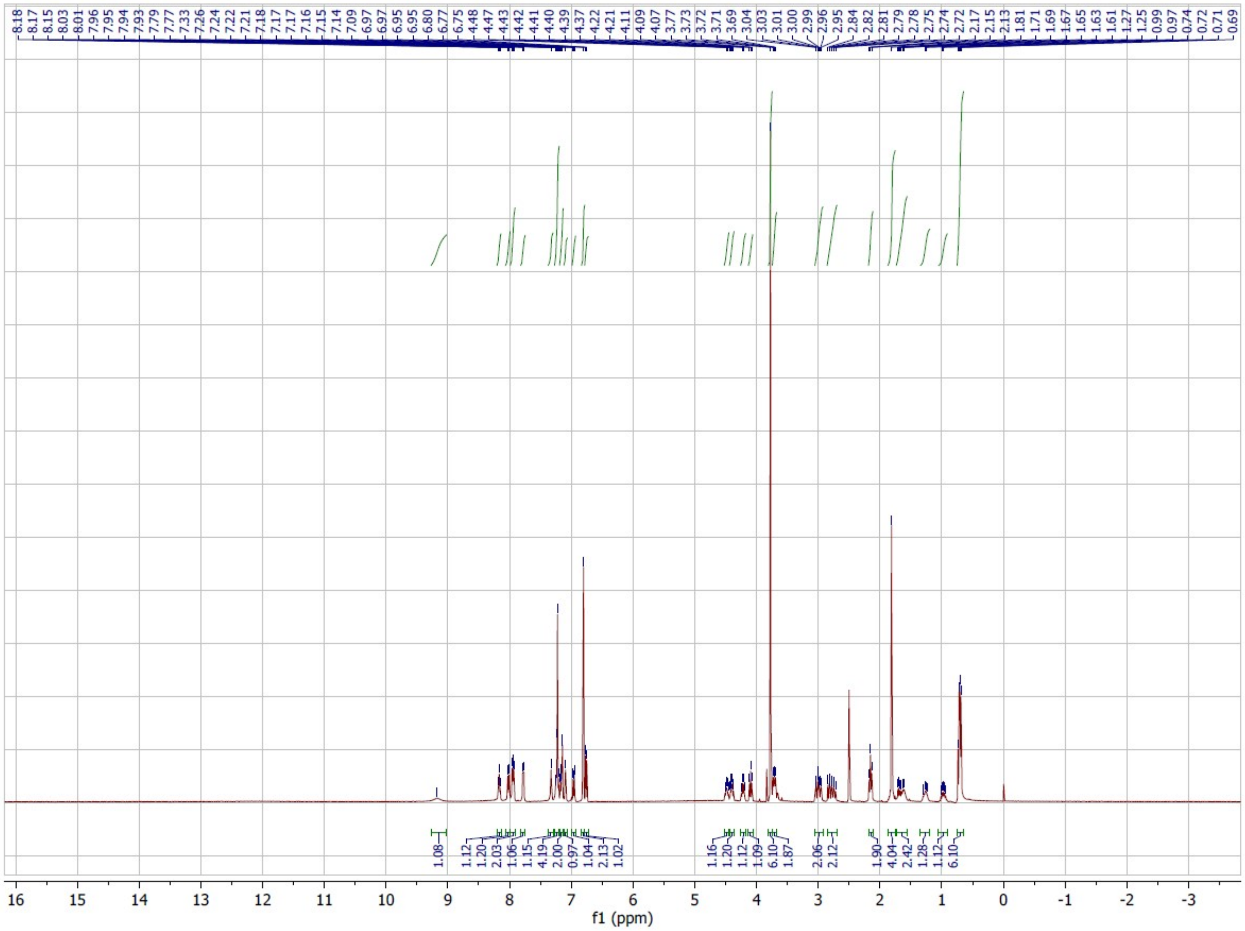

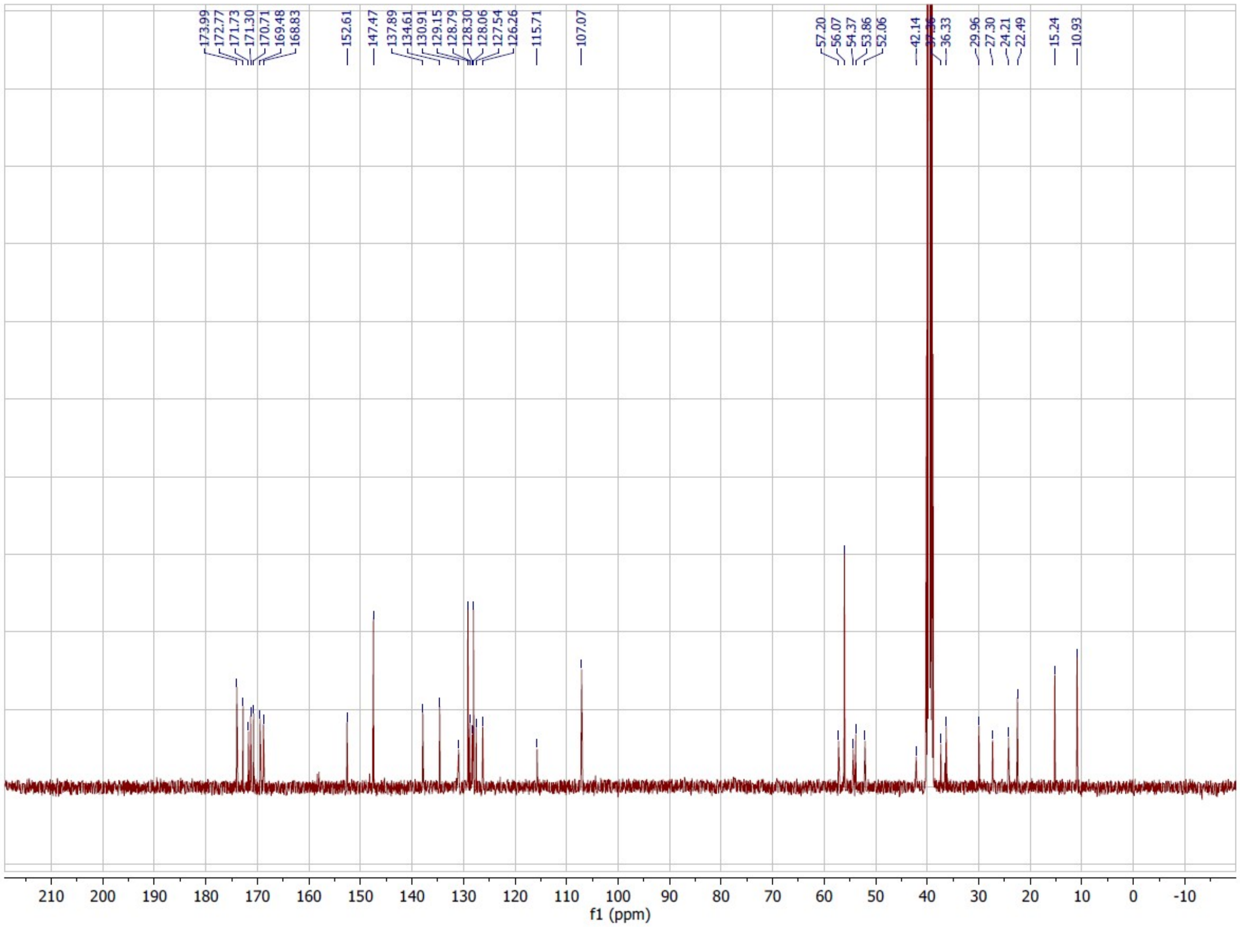

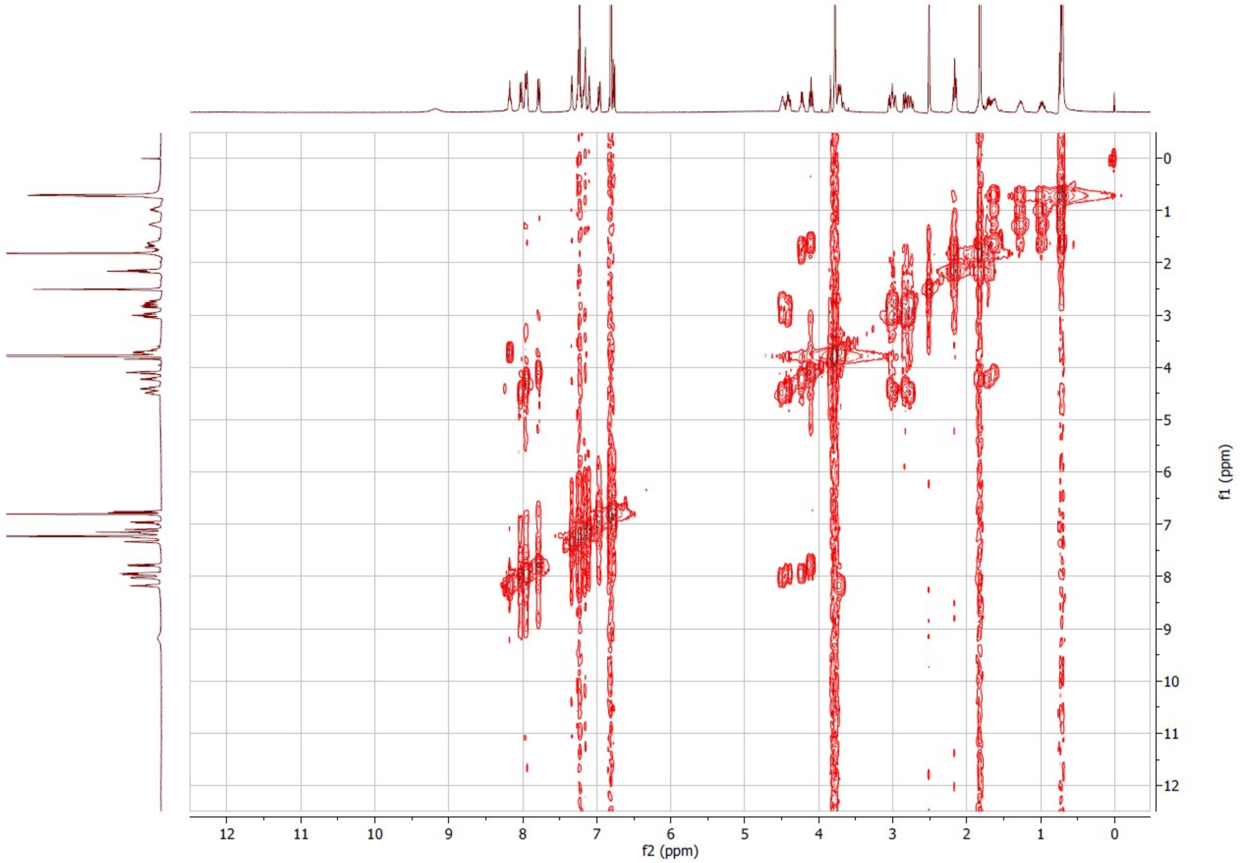
f2 (ppm)
f1 (ppm)

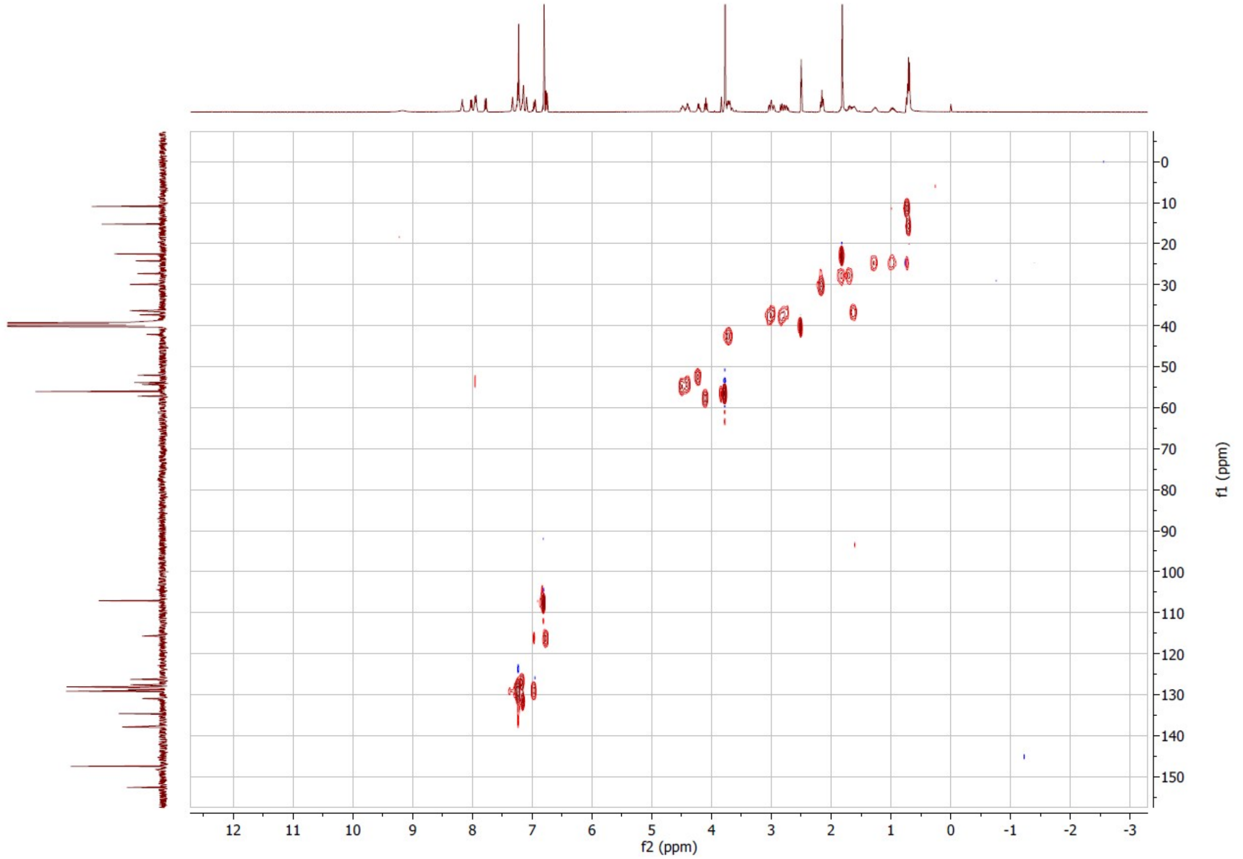
f2 (ppm)
f1 (ppm)

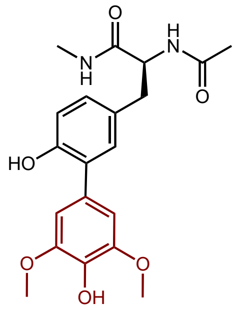

16

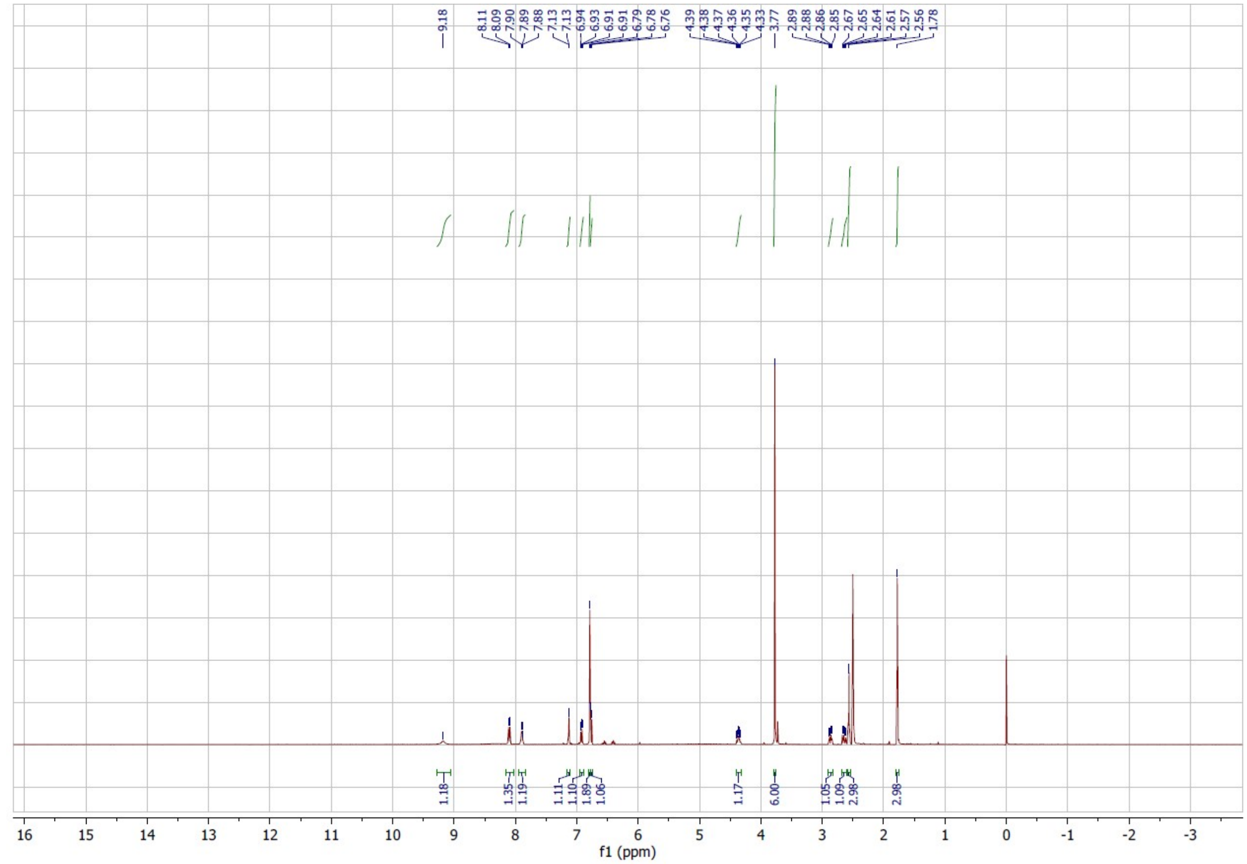

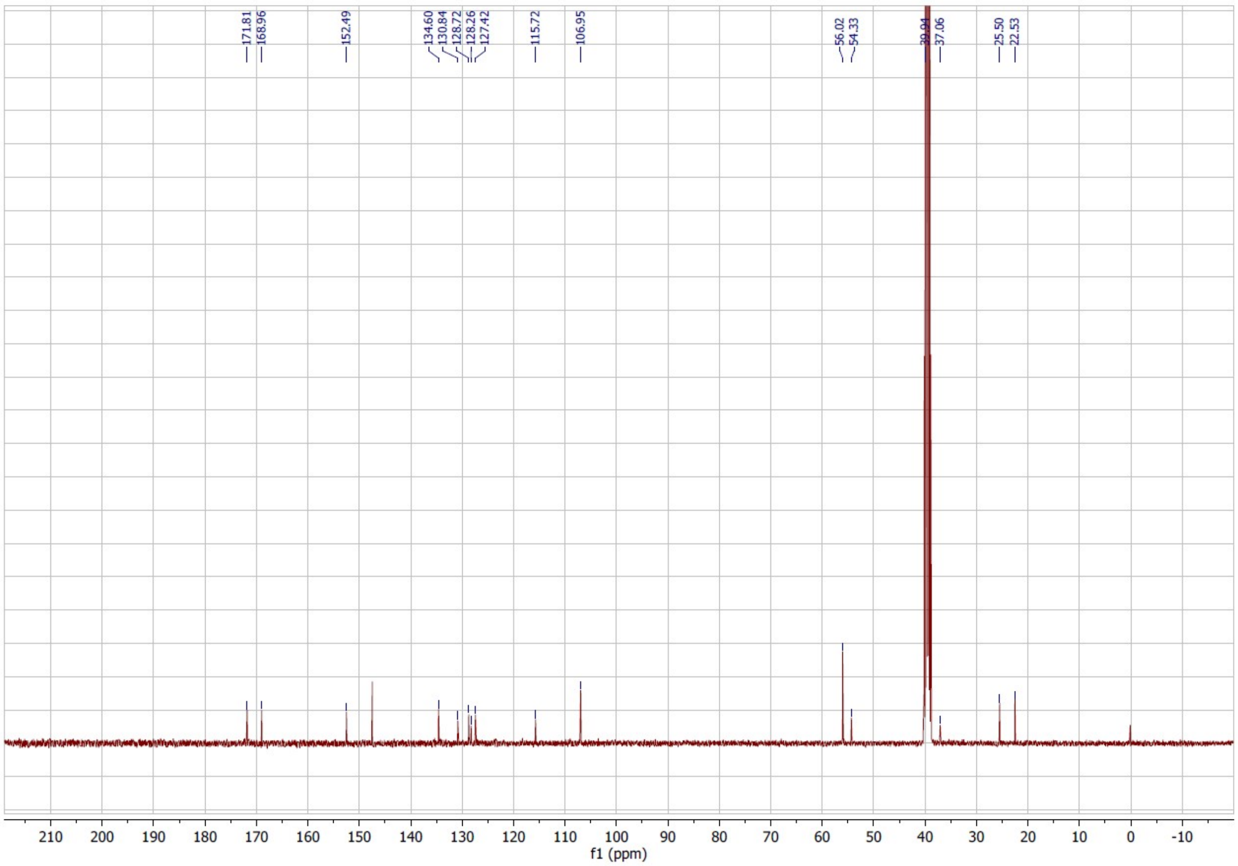

171.81
168.96
152.49
134.60
130.84
128.72
128.26
127.42
115.72
106.95
56.02
54.33
39.94
37.06
25.50
22.53
210 200 190 180 170 160 150 140 130 120 110 100 90 80 70 60 50 40 30 20 10 0 -10
f1 (ppm)

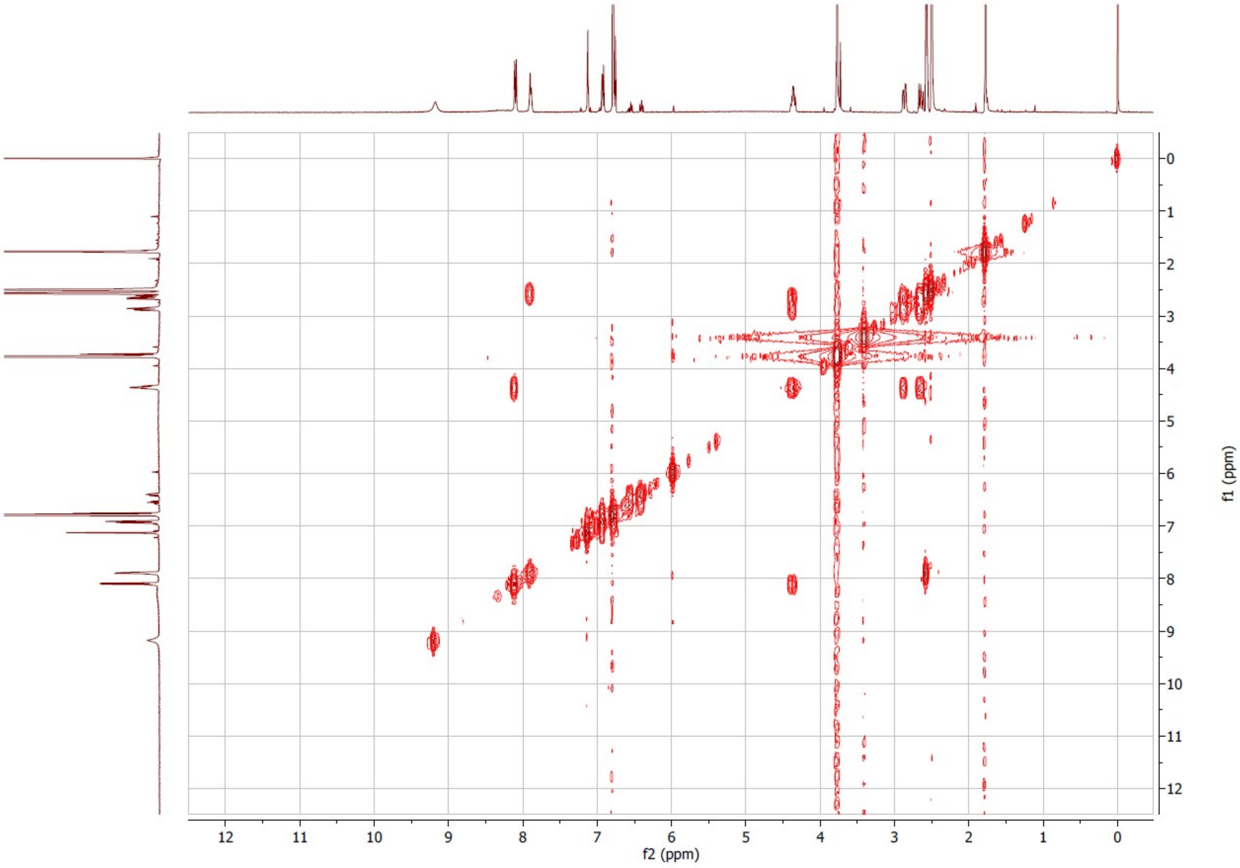

12 11 10 9 8 7 6 5 4 3 2 1 0
f2 (ppm)
0 1 2 3 4 5 6 7 8 9 10 11 12
f1 (ppm)

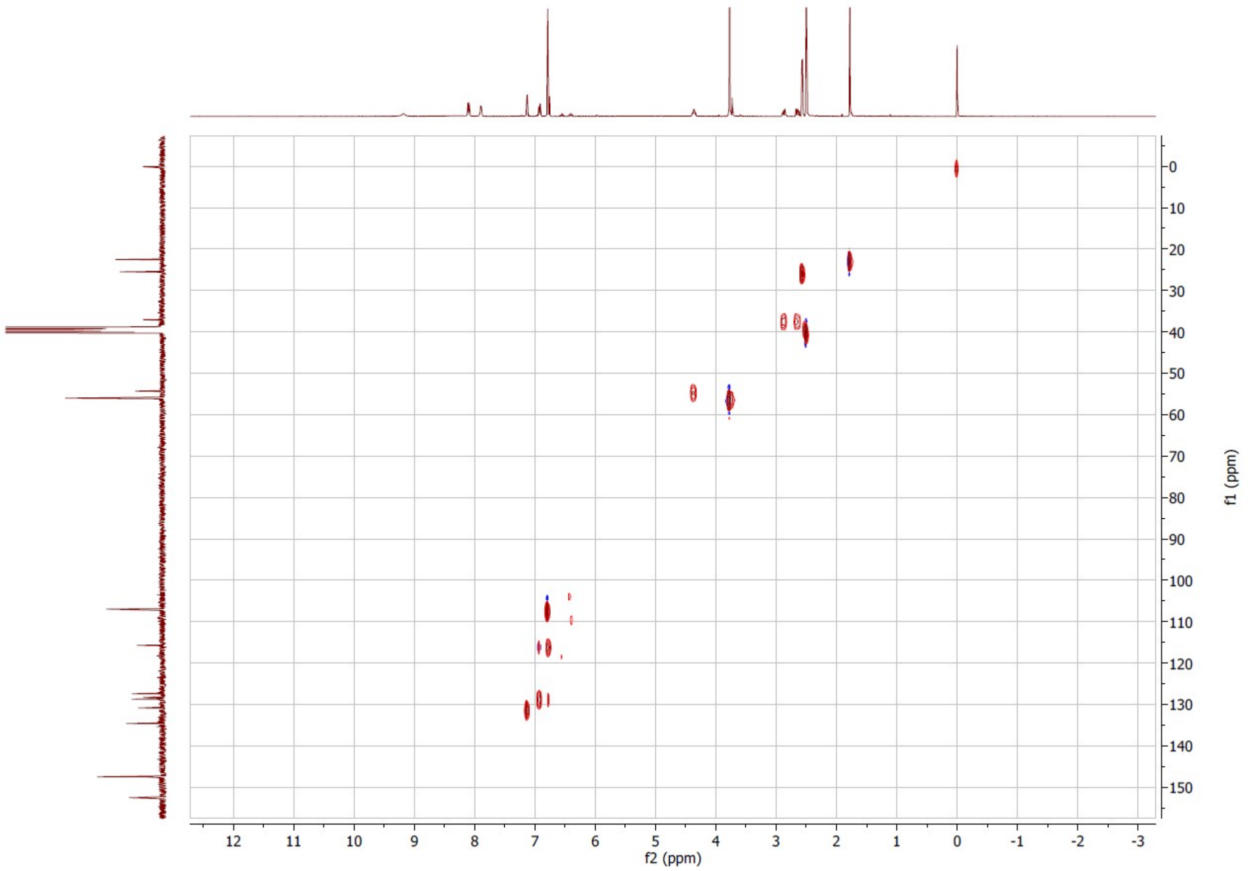

f1 (ppm)
f2 (ppm)

DFT Calculations

Computational methods

Density Functional Theory (DFT) calculations were performed using the Gaussian 09,[13] and Gaussian 16,[14] software at the B3LYP level of theory which uses Becke's 3-parameter exchange[15] and Lee, Yang and Parr's correlation function.[16] All geometry optimization and frequency calculations used the conductor-like polarizable continuum model (CPCM) to simulate the water solvent.[17] Grime's dispersion correction method with Becke-Johnson damping (D3BJ) were applied.[18-19] Geometric optimizations were verified for imaginary frequencies to ensure the optimized structures were local minima for ground states (no imaginary frequency) or saddle points for transition states (one imaginary frequency). Reaction free energies (ΔG) were calculated using the zero-point energy corrected Gibbs free energy at 298.15 K (Sum of Thermal and Free Energies in Gaussian Output), and enthalpies (ΔH) were calculated using the zero-point energy corrected Enthalpy at 298.15K (Sum of electronic and thermal Enthalpies in Gaussian Output). Both geometry and frequency calculations were conducted at the B3LYP-D3BJ/6-311+G(2d,2p) level of theory in solvent, i.e., CPCM(water). The thermochemical results for key steps are resumed in the sections below.

Calculated energies key reactions using B3LYP-D3BJ/6-311+G(2d,2p)//CPCM(water)

Thermodynamic data obtained from DFT calculations. (R = tetraacetate side chain)

Table S5. Oxidation of ^{3}**[RFT]*** with phenol

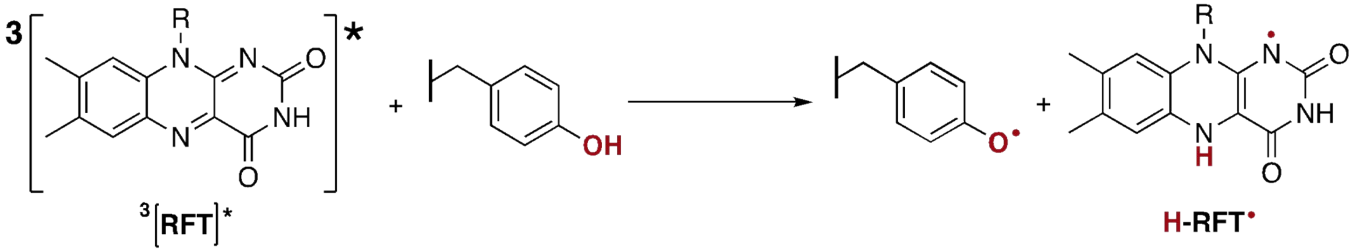

Phenol	ΔG (kcal/mol)	ΔH (kcal/mol)
Biotin tyramide	-20.8	-20.3
Ac-Tyr-NHMe	-20.5	-19.8
2,6-dimethoxyphenol	-26.2	-26.1

Table S6. H-atom abstraction between **H-RFT•** and phenol

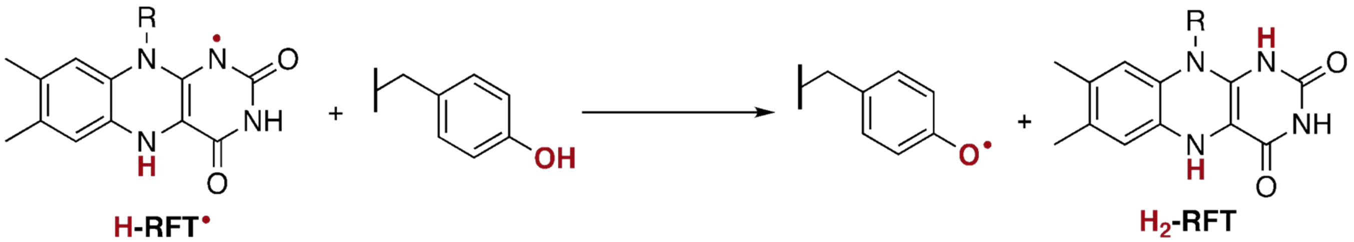

Phenol	ΔG (kcal/mol)	ΔH (kcal/mol)
Biotin tyramide	24.8	24.4
Ac-Tyr-NHMe	25.2	24.9
2,6-dimethoxyphenol	19.4	18.6

Table S7. H-atom abstraction between $^1O_2$ and phenol

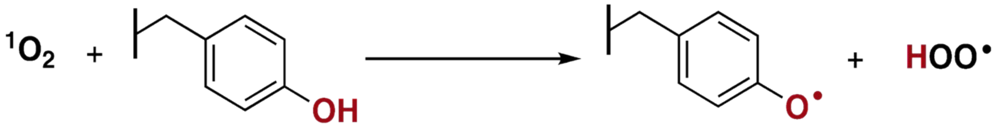

Phenol	ΔG (kcal/mol)	ΔH (kcal/mol)
Biotin tyramide	-12.7	-10.2
Ac-Tyr-NHMe	-12.3	-9.4
2,6-dimethoxyphenol	-18.0	-15.7

Table S8. Photoredox interactions of RFT species with $^3O_2$

$$[RFT]_{reactant} + {}^3O_2 \longrightarrow [RFT]_{product} + [H_xO_2]$$

Entry	$[RFT]_{reactant}$	$[RFT]_{product}$	$[H_xO_2]$	ΔG (kcal/mol)	ΔH (kcal/mol)
**1**	^{3}[RFT]•	RFT	$^1O_2$	-3.18	-5.24
**2**	H_2-RFT	RFT	H_2O_2	-23.42	-22.18
**3**	H-RFT•	RFT	HOO•	+4.99	+5.11

Table S9. Calculated energies for phenoxyl radical-radical recombination pathway using B3LYP/6-311+G(2d,2p)//CPCM(water)

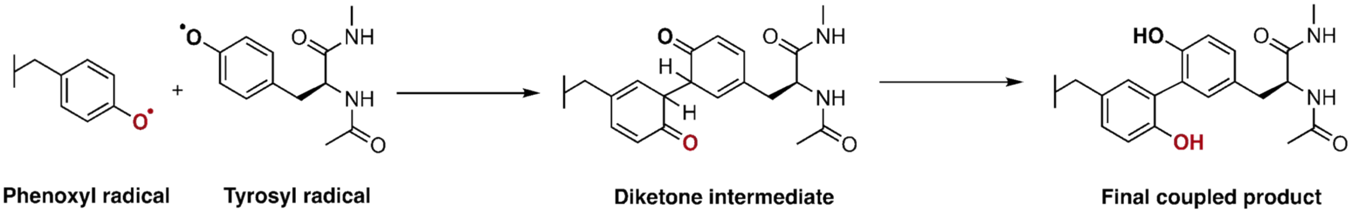

		Diketone intermediate		Final Coupled Product	
**Entry**	**Phenoxyl radical**	**ΔG (kcal/mol)**	**ΔH (kcal/mol)**	**ΔG (kcal/mol)**	**ΔH (kcal/mol)**
**1***	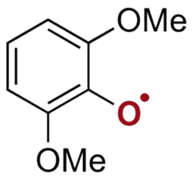	+2.6	-10.3	-35.3	-36.0
**2**	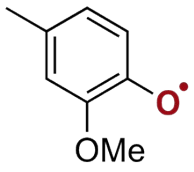	+9.4	-4.3	-37.8	-38.5
**3**	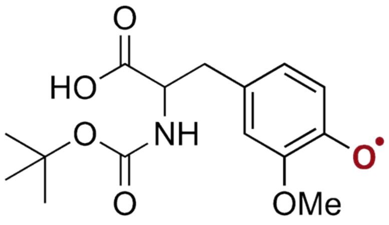	+6.5	-6.1	-38.9	-39.0
**4**	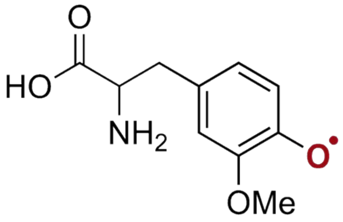	+8.3	-5.5	-39.3	-40.0

**5**	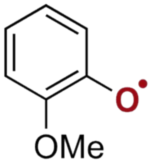	+7.6	-5.3	-37.8	-38.5
**6***	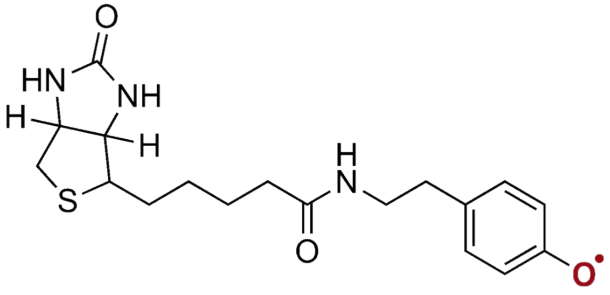	-0.8	-15.7	-35.5	-35.8

***D3BJ dispersion correction used in this calculation.**

Table S10. Calculated energies for the addition of a phenoxyl radical onto a neutral tyrosine using B3LYP-D3BJ/6-311+G(2d,2p)//CPCM(water)

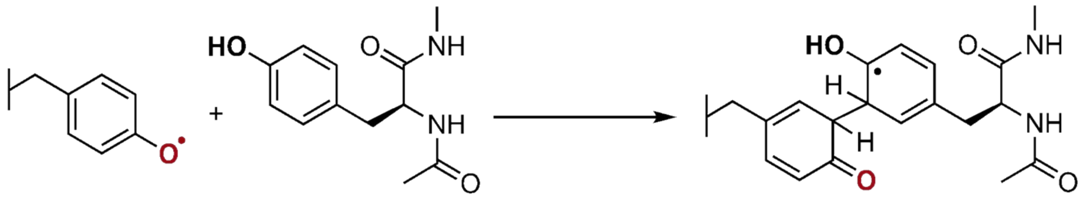

Entry	Phenoxyl radical (#)	ΔG (kcal/mol)	ΔH (kcal/mol)
**1***		+39.6*	+28.0*
**2***	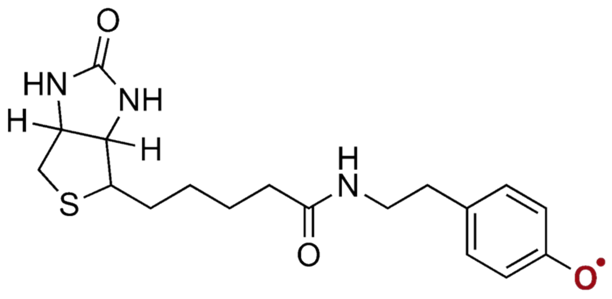	+ 34.3*	+ 21.5*

***The addition product spontaneously dissociates during geometry optimization. To obtain these values, the newly formed bond was frozen at the lowest energy geometry, pre-dissociation. No negative frequencies were obtained, but we cannot be assured that these values were a local minimum in energy.**

Table S11. Comparing DFT functionals: B3LYP-D3BJ vs wB97XD to calculate energies for phenoxyl radical addition onto neutral tyrosine.

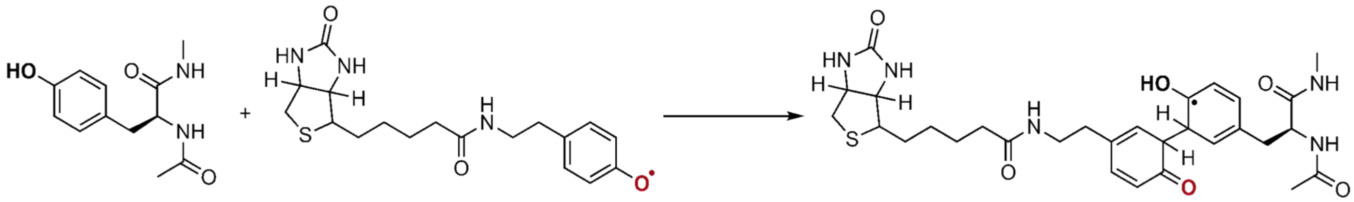

Functional	ΔG (kcal/mol)	ΔH (kcal/mol)
B3LYP-D3BJ	+ 34.3	+ 21.5
wB97XD	+ 32.0	+ 18.1

The geometries were optimized at wB97XD/6-311+G(2d,2p)//CPCM(water). The basis set used for both functionals was 6-311+G(2d,2p). The Gibbs free energy for this proposed step is not significantly different from the value obtained using B3LYP-D3BJ/6-311+G(2d,2p). Both functional's show that the energy for this step is prohibitively unfavorable.

Estimated rate constants for addition of a phenoxyl radical to neutral tyrosine vs radical recombination

With predicted thermodynamic values in hands, relative rates constants of both mechanisms proposed (as shown below) are calculated with the Eyring equation:

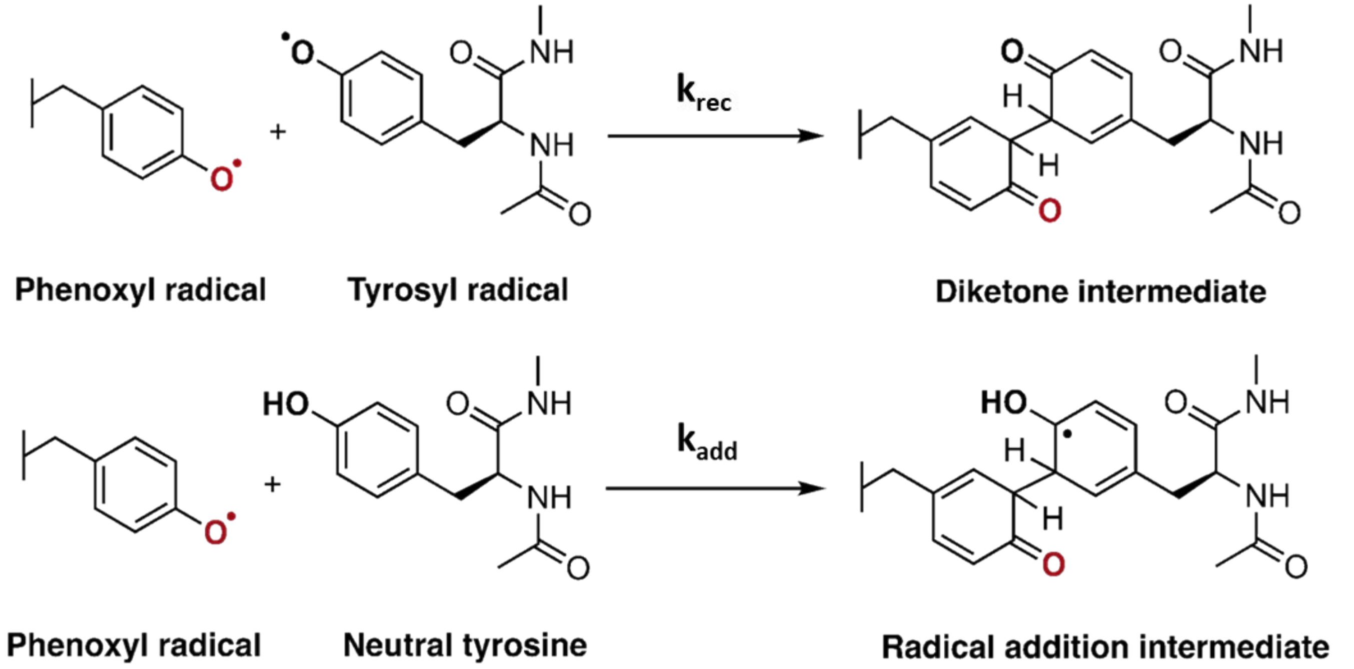

$$k = \frac{\kappa k_B T}{h} e^{-\frac{\Delta G^{\ddagger}}{RT}}$$

where k = rate constant, κ = transmission coefficient (taken to be unity), k_B = 1.380649 x 10^{-23} J·K^{-1} (Boltzmann constant), T = 310 K (temperature), h = 6.626 x 10^{-34} J.s (Planck's constant), $\Delta G^{\ddagger}$ = free energy of activation (J/mol), R = 8.314 J.K^{-1}.mol^{-1} (gas constant).

$\Delta G^{\ddagger}$ values were not calculated directly, but a lower limit for their value can be estimated using the ΔG of each the reaction (see Table S9 and S10). Radical reactions typically have low reaction barriers and this assumption will underestimate the preference for radical recombination as the main reaction pathway.

Biotin tyramide phenoxyl radical *addition* to tyrosine:

$\Delta G^{\ddagger} \simeq \Delta G$ = 34.3 kcal/mol obtained in Table S10.

$k_{add} = \frac{\kappa k_B T}{h} e^{-\frac{\Delta G^{\ddagger}}{RT}} \simeq 4.24 \times 10^{-12}$ M^{-1}s^{-1} for the phenoxyl radical addition onto neutral tyrosine

Biotin tyramide phenoxyl radical *recombination* with a tyrosyl radical:

$\Delta G^{\ddagger} \simeq \Delta G$ = -0.8 kcal/mol obtained in Table S9.

$k_{rec} = \frac{\kappa k_B T}{h} e^{-\frac{\Delta G^{\ddagger}}{RT}} \simeq 2.37 \times 10^{+13}$ M^{-1}s^{-1} for the phenoxyl radical recombination with a tyrosyl radical.

This calculated rate constant is above the diffusion rate constant; we will use 10^9 M^{-1}s^{-1} for this calculation as a standard diffusion limited rate constant.

The ratio of k_{rec} / k_{add}, that is, the kinetic preference for recombination versus addition, is therefore > 2 x 10^{+20}. These estimates show a strong preference for radical recombination. The addition pathway is prohibitively slow and cannot explain protein tagging at physiological temperatures.

Calculated energies for the phenoxyl radical and probe recombination pathway using B3LYP-D3BJ/6-311+G(2d,2p)//CPCM(water) from Sato and Nakamura

The addition of a phenoxyl radical onto a neutral aromatic system appears in the literature in several instances see references 20 and 21 for examples. We postulate this step is almost never favorable as the intermediate formed following phenoxyl radical addition will be prohibitively endergonic. Sato and Nakamura's "phenoxyl radical trap" could possibly occur via the addition of a phenoxyl radical onto the oxidized trap (Table S12), but this reaction is also significantly costly in ΔG. As expected, the recombination of two radicals will be more favorable (Table S13) and the tautomerization to regenerate aromaticity is expected to yield a stable covalent bond.

Table S12. Sato and Nakamura's "phenoxyl radical trap"*

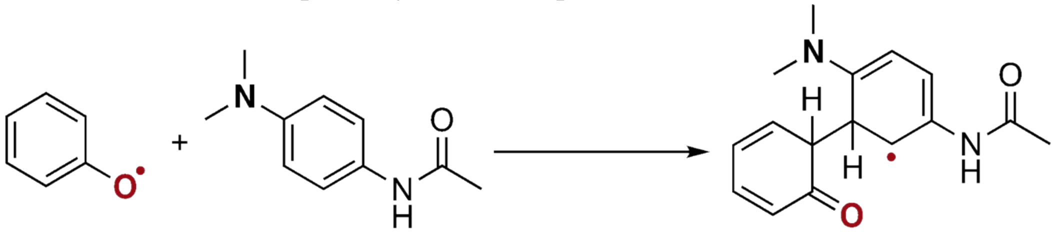

ΔG (kcal/mol)	ΔH (kcal/mol)
+31.8	+19.1

***The addition product spontaneously dissociates during geometry optimization, indicating its unstable nature. To obtain these values, the newly formed bond was frozen at the lowest energy geometry, pre-dissociation. No negative frequencies were obtained, but we cannot be assured that these values were a local minimum in energy**

Table S13. Addition of a phenoxyl radical onto the oxidized "phenoxyl radical trap"

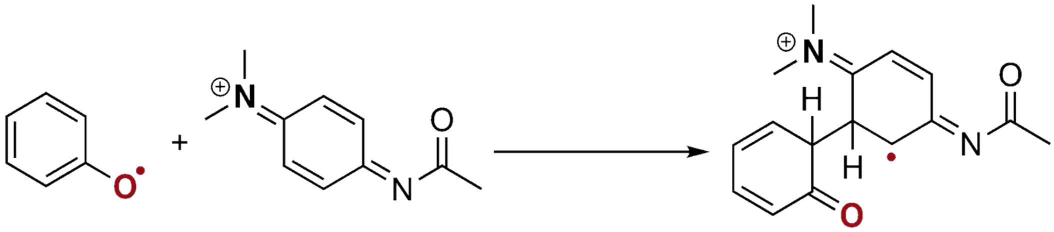

ΔG (kcal/mol)	ΔH (kcal/mol)
+22.6	+9.1

Table S14. Radical recombination between phenoxyl radical onto the "phenoxyl radical trap"

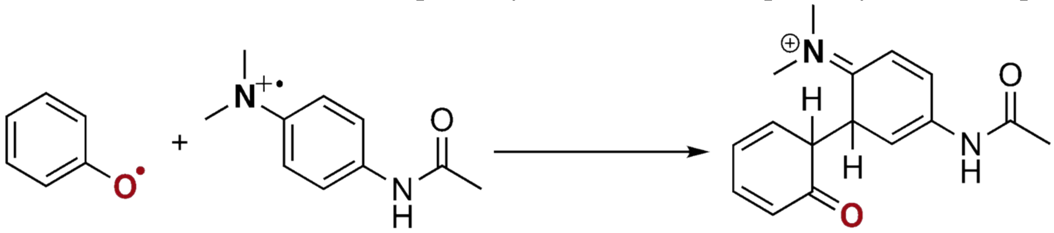

ΔG (kcal/mol)	ΔH (kcal/mol)
+9.9	-4.3

Calculated energies for proposed coupling of a peptide and probe using B3LYP-D3BJ/6-311+G(2d,2p)//CPCM(water) from MacMillan Site Selective Flavin Photoredox

In a recent article,[22] Li *et al.* proposed a radical addition onto a neutral tyrosine which we also find to be energetically unfavorable (first entry below); the recombination of these two persistent radicals is more favorable and the final product, following a tautomerization to regenerate aromaticity, yields a strong covalent bond (second entry).

Table S15. Calculated energies for the addition of a dialdehyde phenoxazin radical onto a neutral tyrosine using B3LYP-D3BJ/6-311+G(2d,2p)//CPCM(water)

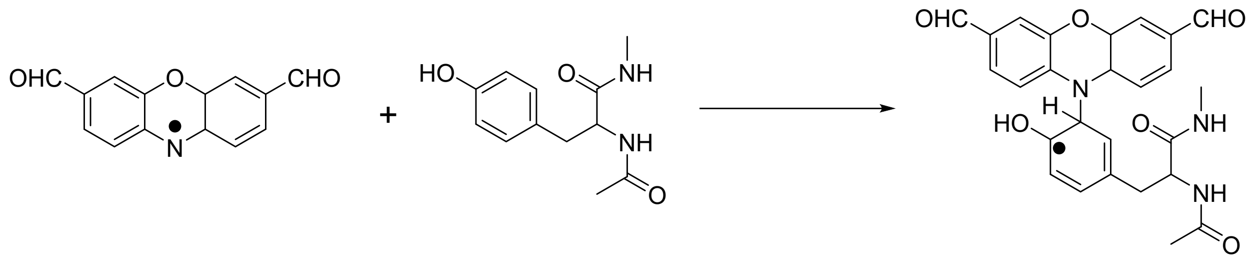

ΔG (kcal/mol)	ΔH (kcal/mol)
+32.0	+18.2

Table S16. Calculated energies for the radical-radical recombination dialdehyde phenoxazine radical and tyrosyl radical using B3LYP-D3BJ/6-311+G(2d,2p)//CPCM(water)

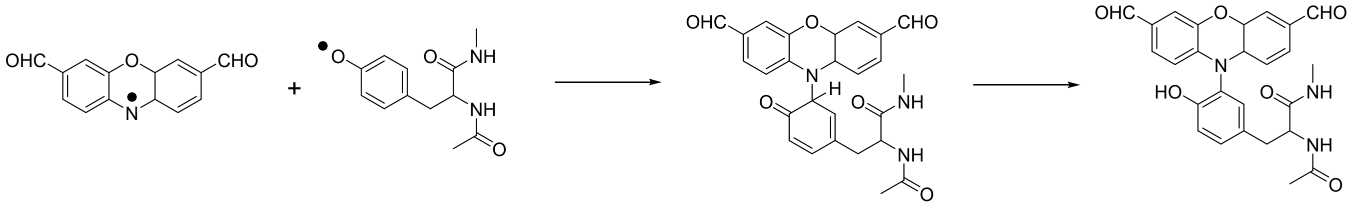

	Ketone intermediate	Final product
ΔG (kcal/mol)	-4.6	-23.5
ΔH (kcal/mol)	-18.6	-24.5

DFT energies and coordinates for selected compounds as obtained from B3LYP-D3BJ/6-311+G (2d,2p) //CPCM(water)

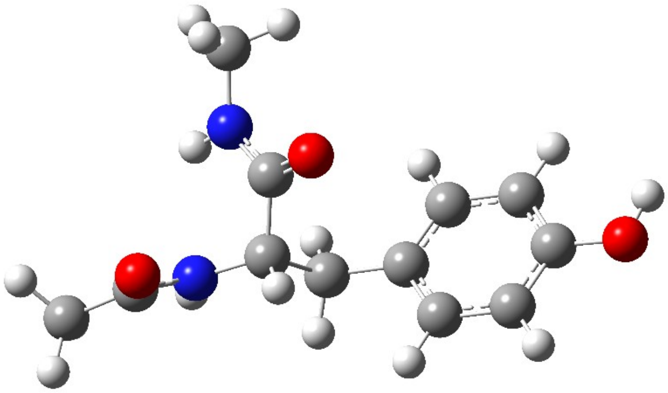

peptide-phenol_B3LYP-D3BJ_6-311_plus_2d2p_H2O_maxdisk8GB

Zero-point correction= 0.271054 (Hartree/Particle)
Thermal correction to Energy= 0.288969
Thermal correction to Enthalpy= 0.289913
Thermal correction to Gibbs Free Energy= 0.222786
Sum of electronic and zero-point Energies= -802.200121
Sum of electronic and thermal Energies= -802.182206
Sum of electronic and thermal Enthalpies= -802.181262
Sum of electronic and thermal Free Energies= -802.248389

C	1.363055	-0.714342	-0.654371
C	2.041663	0.425701	-1.079235
C	3.347296	0.682355	-0.675209
C	3.994791	-0.212963	0.171288
C	3.33534	-1.361604	0.603344
C	2.033233	-1.602551	0.188927
H	1.545752	1.13076	-1.732839
H	1.530468	-2.498491	0.528848
O	5.279728	-0.018908	0.606156
H	3.859042	1.572603	-1.016734
H	3.848673	-2.054471	1.254659
C	-0.06164	-0.969498	-1.067806
C	-1.102264	-0.578272	0.002376
H	-0.203903	-2.029746	-1.281697
H	-0.28375	-0.421402	-1.984284
H	-0.927973	-1.154186	0.90927
C	-3.53022	-0.856123	0.273947
C	-4.827883	-1.270571	-0.375874
H	-5.528454	-0.438763	-0.317608
C	-0.94759	0.891531	0.41597
N	-1.550534	1.791701	-0.376641
H	-2.166453	1.454825	-1.096595
O	-0.258839	1.203217	1.386212
N	-2.427737	-0.87996	-0.514438

H	-2.509473	-1.232924	-1.453117
O	-3.470716	-0.50214	1.450427
H	5.625635	0.804311	0.244941
H	-4.711038	-1.56561	-1.415846
H	-5.250533	-2.101566	0.186689
C	-1.478876	3.220978	-0.125178
H	-0.441138	3.546002	-0.080873
H	-1.979633	3.741282	-0.935824
H	-1.962137	3.479981	0.817119

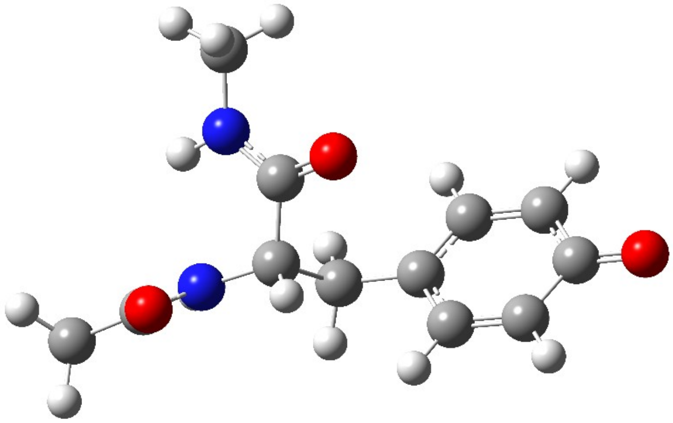

peptide-phenol_rad_B3LYP-D3BJ_6-311_plus_2d2p_H2O_maxdisk8GB
Zero-point correction= 0.258301 (Hartree/Particle)
Thermal correction to Energy= 0.275985
Thermal correction to Enthalpy= 0.276930
Thermal correction to Gibbs Free Energy= 0.208947
Sum of electronic and zero-point Energies= -801.569587
Sum of electronic and thermal Energies= -801.551903
Sum of electronic and thermal Enthalpies= -801.550959
Sum of electronic and thermal Free Energies= -801.618941

C	-1.420883	-0.656001	0.657379
C	-2.150027	0.45968	1.117765
C	-3.435909	0.682265	0.705811
C	-4.086242	-0.22305	-0.216609
C	-3.31797	-1.363799	-0.66903
C	-2.035887	-1.559585	-0.238396
H	-1.675724	1.147297	1.804451
H	-1.476525	-2.419413	-0.581698
O	-5.2657	-0.029532	-0.605622
H	-4.000725	1.535819	1.052255
H	-3.796302	-2.050714	-1.352367
C	-0.00936	-0.885295	1.10547
C	1.04594	-0.511139	0.039554
H	0.130183	-1.942715	1.335019
H	0.189963	-0.318454	2.014613
H	0.869679	-1.08794	-0.865961
C	3.439856	-0.955037	-0.262841

C	4.730395	-1.386956	0.388395
H	5.484216	-0.621608	0.2103
C	0.895885	0.957986	-0.378127
N	1.659238	1.846935	0.271532
H	2.357677	1.501175	0.907137
O	0.067493	1.270603	-1.232568
N	2.362976	-0.835781	0.556183
H	2.454295	-1.103529	1.521738
O	3.360298	-0.710956	-1.464717
H	4.635796	-1.552652	1.458748
H	5.070063	-2.305847	-0.08718
C	1.601052	3.271916	-0.009306
H	0.583126	3.639172	0.105745
H	2.248292	3.789741	0.691542
H	1.932657	3.484763	-1.025487

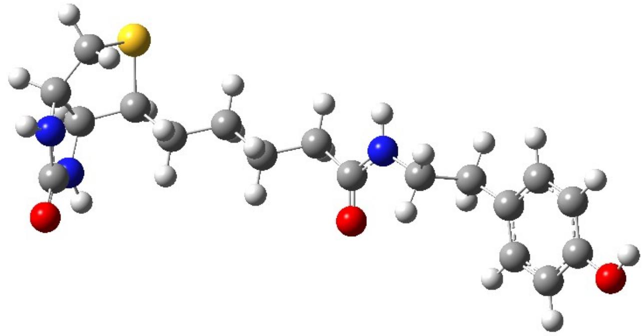

tag-phenol_B3LYP-D3BJ_6-311_plus_2d2p_H2O
Zero-point correction= 0.420187 (Hartree/Particle)
Thermal correction to Energy= 0.444977
Thermal correction to Enthalpy= 0.445921
Thermal correction to Gibbs Free Energy= 0.358789
Sum of electronic and zero-point Energies= -1489.228593
Sum of electronic and thermal Energies= -1489.203804
Sum of electronic and thermal Enthalpies= -1489.202859
Sum of electronic and thermal Free Energies= -1489.289991

C	-7.681837	1.305065	-0.549773
C	-6.464867	0.69348	-0.821068
C	-8.494374	0.803088	0.46334
C	-6.031997	-0.422146	-0.100587
H	-5.841948	1.089956	-1.612224
C	-8.083333	-0.306989	1.196245
C	-6.861577	-0.906991	0.910558
H	-8.715017	-0.70095	1.981853
H	-6.554127	-1.771128	1.484898
O	-9.688675	1.43666	0.693177
H	-10.158251	0.995736	1.409311
C	-4.687688	-1.045531	-0.371557
C	-3.578246	-0.384181	0.462253

H	-4.43105	-0.946165	-1.426191
H	-4.712676	-2.111227	-0.14079
H	-3.536978	0.679695	0.24016
H	-3.792359	-0.497981	1.523536
C	-1.462416	-0.524767	-0.79761
C	-0.139808	-1.249201	-0.945206
C	1.046823	-0.325606	-0.646537
H	-0.077457	-1.60117	-1.976425
H	-0.1017	-2.125775	-0.297704
C	2.390369	-1.021535	-0.851398
H	0.982438	0.553671	-1.289138
H	0.974826	0.03111	0.384121
C	3.573413	-0.105674	-0.549632
H	2.444618	-1.906979	-0.215374
H	2.462047	-1.37489	-1.884197
H	3.509579	0.772716	-1.195634
H	3.505654	0.26265	0.475424
N	-2.264465	-0.950115	0.202325
H	-1.977402	-1.753344	0.734534
O	-1.769851	0.415645	-1.535085
C	5.842287	-0.974029	1.6824
C	6.508217	0.200213	0.949722
C	4.929899	-0.769168	-0.77915
C	6.159347	0.146247	-0.56801
C	5.786789	2.318673	0.239086
S	5.276442	-2.150537	0.38883
H	6.990576	-0.269134	-1.130479
H	7.588389	0.16076	1.08518
N	5.978534	1.553485	-0.882213
H	5.608554	1.869616	-1.762151
N	5.988268	1.509658	1.334104
H	6.280864	1.946693	2.192063
O	5.488149	3.5088	0.263385
H	4.960539	-1.200796	-1.778323
H	6.527131	-1.498336	2.341072
H	4.991969	-0.626216	2.262246
H	-8.010644	2.164344	-1.116835

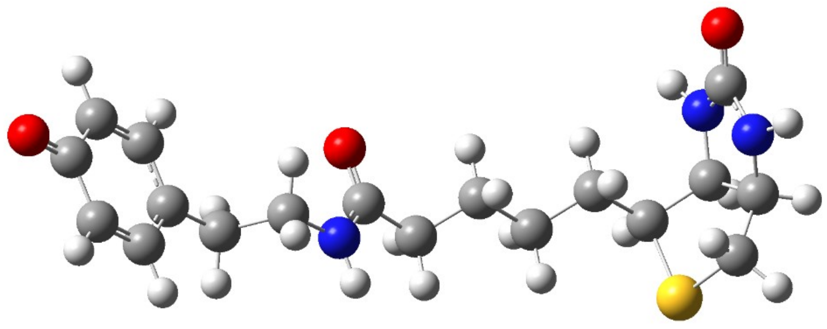

tag-phenol_rad_B3LYP-D3BJ_6-311_plus_2d2p_H2O_maxdisk8GB

Zero-point correction= 0.407432 (Hartree/Particle)
Thermal correction to Energy= 0.431962
Thermal correction to Enthalpy= 0.432906
Thermal correction to Gibbs Free Energy= 0.345168
Sum of electronic and zero-point Energies= -1488.598847
Sum of electronic and thermal Energies= -1488.574316
Sum of electronic and thermal Enthalpies= -1488.573372
Sum of electronic and thermal Free Energies= -1488.661111

C	-7.635021	1.411492	-0.499541
C	-6.447118	0.791762	-0.775476
C	-8.53893	0.873174	0.494242
C	-6.065592	-0.391867	-0.104191
H	-5.782146	1.204357	-1.522288
C	-8.125534	-0.337972	1.169759
C	-6.93084	-0.934853	0.871478
H	-8.794061	-0.750121	1.912067
H	-6.636476	-1.841119	1.38353
O	-9.634497	1.431386	0.757599
C	-4.738814	-1.023455	-0.382798
C	-3.634174	-0.417547	0.513168
H	-4.456348	-0.864578	-1.422606
H	-4.784558	-2.097012	-0.202267
H	-3.583739	0.656866	0.354479
H	-3.86537	-0.596397	1.560911
C	-1.53437	-0.527064	-0.764685
C	-0.212582	-1.244226	-0.943373
C	0.974572	-0.325333	-0.631641
H	-0.159462	-1.569036	-1.983912
H	-0.166741	-2.137017	-0.319126
C	2.31732	-1.015344	-0.860067
H	0.904933	0.567799	-1.254224
H	0.908609	0.008266	0.407069
C	3.501545	-0.103368	-0.551255
H	2.377671	-1.911986	-0.24049
H	2.381305	-1.349821	-1.899606
H	3.435058	0.783392	-1.185412
H	3.438237	0.251546	0.478804
N	-2.328507	-0.981468	0.231461
H	-2.043076	-1.802588	0.73653
O	-1.853503	0.432285	-1.470234
C	5.78206	-0.99302	1.659575
C	6.444554	0.187889	0.934516
C	4.856723	-0.764593	-0.794938
C	6.087543	0.14849	-0.581752
C	5.720207	2.313281	0.247967
S	5.209022	-2.157111	0.357945

H	6.915601	-0.26177	-1.152584
H	7.525413	0.146694	1.063787
N	5.905497	1.558678	-0.88156
H	5.531321	1.883385	-1.75659
N	5.927316	1.493822	1.334128
H	6.224736	1.922474	2.194671
O	5.42217	3.503226	0.285176
H	4.88177	-1.186518	-1.7984
H	6.470136	-1.523693	2.309718
H	4.934873	-0.650452	2.247079
H	-7.934844	2.314043	-1.012704

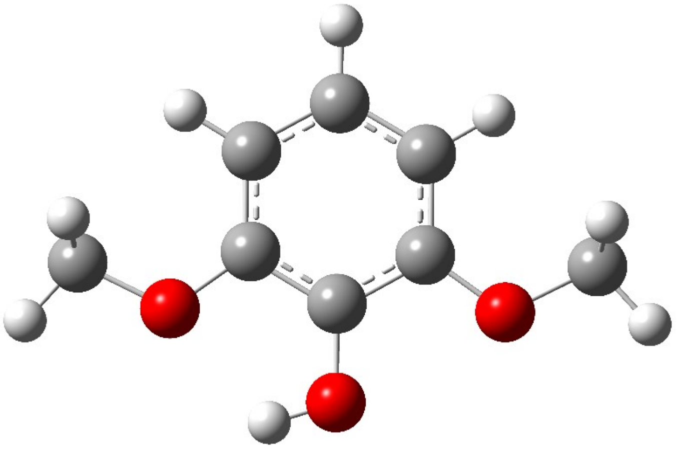

2-6-dimethoxyphenol_B3LYP-D3BJ_6-311_plus_2d2p_H2O
Zero-point correction= 0.169625 (Hartree/Particle)
Thermal correction to Energy= 0.180328
Thermal correction to Enthalpy= 0.181272
Thermal correction to Gibbs Free Energy= 0.133475
Sum of electronic and zero-point Energies= -536.562109
Sum of electronic and thermal Energies= -536.551406
Sum of electronic and thermal Enthalpies= -536.550462
Sum of electronic and thermal Free Energies= -536.598260

C	0.038911	2.091986	-0.000018
C	-1.185559	1.432225	-0.000022
C	-1.197458	0.040228	-0.000017
C	-0.001561	-0.682477	-0.000008
C	1.222403	-0.009096	-0.000005
C	1.237717	1.387243	-0.000011
H	-2.1061	1.992933	-0.000029
H	2.172995	1.92236	-0.000009
O	-0.01767	-2.049901	-0.000001
O	-2.314848	-0.74846	-0.000026
C	-3.590093	-0.105163	0.000051
H	-4.325228	-0.902812	0.000077
H	-3.713517	0.509453	0.891829
H	-3.713612	0.50948	-0.891695
H	0.059724	3.1721	-0.000022

O	2.331003	-0.802473	0.000002
C	3.604749	-0.159127	0.000023
H	4.339342	-0.957769	0.000036
H	3.733921	0.456229	-0.891154
H	3.73389	0.456232	0.891202
H	-0.943932	-2.326448	0.000006

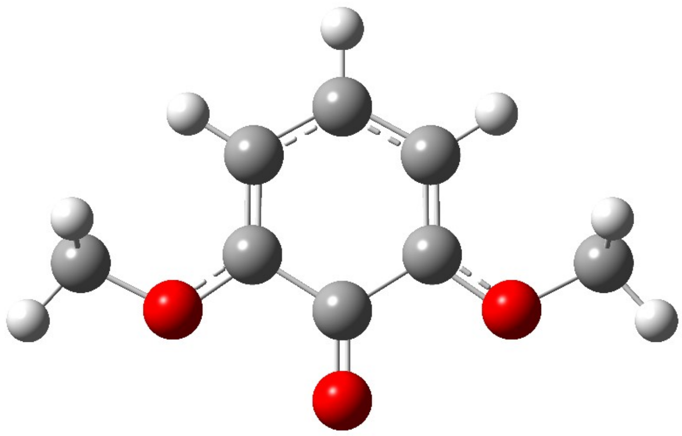

2-6-dimethoxyphenol_rad_B3LYP-D3BJ_6-311_plus_2d2p_H2O
Zero-point correction= 0.157225 (Hartree/Particle)
Thermal correction to Energy= 0.167588
Thermal correction to Enthalpy= 0.168532
Thermal correction to Gibbs Free Energy= 0.120818
Sum of electronic and zero-point Energies= -535.941556
Sum of electronic and thermal Energies= -535.931194
Sum of electronic and thermal Enthalpies= -535.930249
Sum of electronic and thermal Free Energies= -535.977963

C	-0.000007	-2.027994	-0.000016
C	1.226096	-1.356595	-0.00001
C	1.244655	0.024271	-0.000001
C	0	0.796774	0.000003
C	-1.244651	0.024274	-0.000004
C	-1.226093	-1.35661	-0.000014
H	2.140651	-1.926526	-0.000014
H	-2.140662	-1.926519	-0.000019
O	0	2.043636	0.000014
O	2.348032	0.784281	0.000005
C	3.621635	0.128301	0.000022
H	4.359902	0.92226	0.000041
H	3.736842	-0.486262	0.892239
H	3.736873	-0.486247	-0.892201
H	0.000012	-3.108384	-0.000024
O	-2.348025	0.784274	-0.000002
C	-3.621643	0.128307	-0.000004
H	-4.359893	0.922282	0.000002

H	-3.736868	-0.486236	-0.892231
H	-3.736866	-0.486247	0.892214

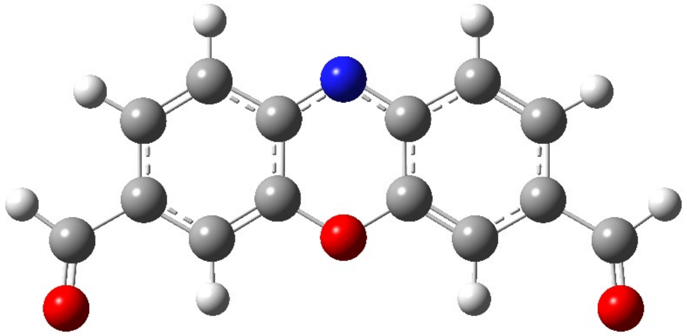

phenoxazine_rad_B3LYP-D3BJ_6-311_plus_2d2p_H2O
Zero-point correction= 0.185845 (Hartree/Particle)
Thermal correction to Energy= 0.199456
Thermal correction to Enthalpy= 0.200400
Thermal correction to Gibbs Free Energy= 0.144281
Sum of electronic and zero-point Energies= -818.827405
Sum of electronic and thermal Energies= -818.813795
Sum of electronic and thermal Enthalpies= -818.812851
Sum of electronic and thermal Free Energies= -818.868970

C	3.572978	1.180352	0.000011
C	2.395655	1.889086	0.000006
C	1.152798	1.215719	0.000004
C	1.171887	-0.207322	0.000006
C	2.349658	-0.921054	0.000011
C	3.561136	-0.227595	0.000014
C	-1.171887	-0.207322	-0.000001
C	-1.152798	1.215719	-0.000004
C	-2.395655	1.889086	-0.000009
H	-2.38453	2.968754	-0.000011
C	-3.572978	1.180351	-0.000012
C	-3.561136	-0.227595	-0.00001
C	-2.349658	-0.921054	-0.000004
H	4.5207	1.70061	0.000013
H	2.38453	2.968754	0.000004
H	2.333572	-2.000625	0.000014
H	-4.5207	1.70061	-0.000016
H	-2.333572	-2.000625	-0.000002
O	0	-0.903922	0.000005
N	0	1.92486	-0.000001
C	4.838199	-0.953301	0.000022
H	5.735596	-0.312668	-0.00004
C	-4.838198	-0.953301	-0.000015

H	-5.735597	-0.312668	0.00002
O	4.945284	-2.166588	-0.000042
O	-4.945284	-2.166588	0.000023

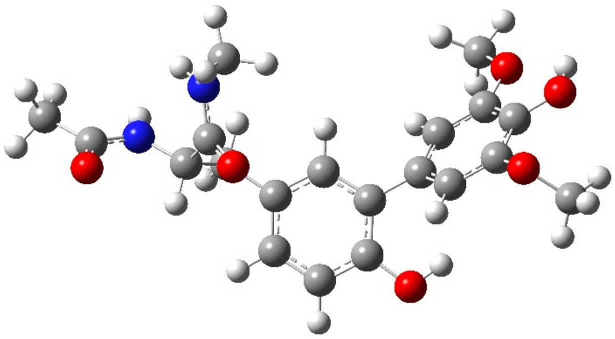

2-6-dimethoxyphenol_plus_peptide-phenol_B3LYP-D3BJ_6-311_plus_2d2p_H2O
Zero-point correction= 0.420520 (Hartree/Particle)
Thermal correction to Energy= 0.449820
Thermal correction to Enthalpy= 0.450765
Thermal correction to Gibbs Free Energy= 0.356681
Sum of electronic and zero-point Energies= -1337.585202
Sum of electronic and thermal Energies= -1337.555901
Sum of electronic and thermal Enthalpies= -1337.554957
Sum of electronic and thermal Free Energies= -1337.649041

C	1.616724	1.240561	-1.174102
C	0.434058	0.563265	-0.897646
C	-0.753785	1.228373	-0.586583
C	-0.741571	2.63164	-0.579742
C	0.43105	3.326193	-0.863759
C	1.598287	2.635382	-1.152516
H	0.430357	-0.518596	-0.896731
H	2.503258	3.189143	-1.364668
O	-1.863228	3.376444	-0.333589
H	0.410507	4.4067	-0.864769
C	2.890285	0.4826	-1.435729
C	3.775367	0.306752	-0.183025
H	3.489316	0.999898	-2.186014
H	2.653606	-0.505431	-1.832817
H	4.102029	1.281737	0.173619
C	5.998807	-0.638691	0.278023
C	7.164653	-1.428417	-0.265233
H	7.358662	-2.267443	0.401113
C	2.976482	-0.304914	0.97616
N	2.839914	-1.640143	0.955253
H	3.37032	-2.163692	0.280185
O	2.449756	0.412318	1.825023
N	4.935424	-0.489997	-0.549437
H	4.998721	-0.844009	-1.489176
O	6.002047	-0.162783	1.412215

H	6.995764	-1.803431	-1.271707
H	8.04833	-0.79185	-0.264913
C	2.07477	-2.359075	1.959719
H	1.051955	-1.988121	1.993928
H	2.061894	-3.412353	1.697043
H	2.517413	-2.24368	2.949116
C	-1.991283	0.465606	-0.294721
C	-2.433772	-0.515303	-1.192147
C	-2.722692	0.698185	0.870253
C	-3.579709	-1.24523	-0.915617
H	-1.879919	-0.68935	-2.10032
C	-3.876908	-0.031548	1.146547
H	-2.388465	1.415868	1.605907
C	-4.311549	-1.009822	0.259188
H	-2.613244	2.784406	-0.187938
O	-5.429483	-1.737761	0.534853
H	-5.564288	-2.357974	-0.19471
O	-4.543646	0.178171	2.330633
O	-4.108552	-2.22439	-1.702767
C	-5.778076	0.902643	2.190848
H	-6.471895	0.366839	1.544651
H	-6.196401	0.988063	3.189317
H	-5.585956	1.897154	1.785493
C	-3.429807	-2.552712	-2.917907
H	-4.007436	-3.349397	-3.373549
H	-3.397122	-1.691756	-3.585342
H	-2.417914	-2.900666	-2.711437

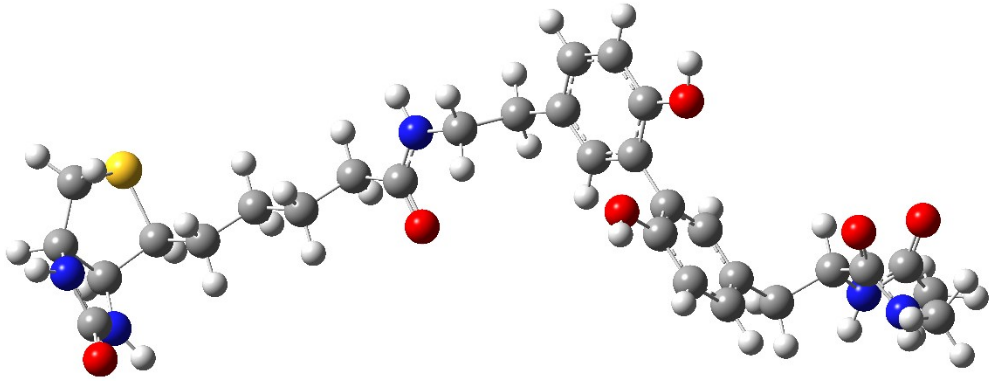

peptide-phenol_plus_tag-phenol_B3LYP-D3BJ_6-311_plus_2d2p_H2O
Zero-point correction= 0.671634 (Hartree/Particle)
Thermal correction to Energy= 0.714860
Thermal correction to Enthalpy= 0.715804
Thermal correction to Gibbs Free Energy= 0.584082
Sum of electronic and zero-point Energies= -2290.250414
Sum of electronic and thermal Energies= -2290.207188

Sum of electronic and thermal Enthalpies= -2290.206244
Sum of electronic and thermal Free Energies= -2290.337966

C	5.329601	-0.847818	0.991061
C	5.517092	-0.661007	2.357699
C	4.955087	0.428624	3.010878
C	4.199339	1.356941	2.301223
C	4.001006	1.200618	0.922713
C	4.566556	0.090585	0.298583
H	6.106619	-1.370434	2.922556
H	4.409003	-0.031754	-0.765172
O	3.600502	2.421398	2.917477
H	5.094183	0.555118	4.076876
C	5.959103	-2.008641	0.268474
C	7.244091	-1.638153	-0.501231
H	5.257016	-2.421975	-0.45709
H	6.195243	-2.800804	0.980031
H	7.018078	-0.873734	-1.242383
C	8.725206	-2.773731	-2.101851
C	9.075365	-4.073008	-2.785448
H	10.132417	-4.278836	-2.624503
C	8.282816	-1.005673	0.434126
N	9.055087	-1.86197	1.121465
H	9.004544	-2.839513	0.890938
O	8.34281	0.215007	0.572466
N	7.740921	-2.829661	-1.171064
H	7.246879	-3.696424	-1.040039
O	9.304338	-1.72026	-2.361715
H	3.809362	2.408301	3.857461
H	8.48895	-4.91616	-2.428531
H	8.918404	-3.956815	-3.85688
C	10.069716	-1.411868	2.059019
H	9.623929	-0.784842	2.828951
H	10.519856	-2.282038	2.526599
H	10.845942	-0.837606	1.552936
C	3.180654	2.159338	0.140381
C	1.985264	1.744253	-0.445755
C	3.594889	3.481267	-0.065558
C	1.190085	2.596122	-1.211598
H	1.669348	0.720703	-0.290542
C	2.814678	4.349774	-0.823423
C	1.622879	3.909644	-1.385776
H	3.148909	5.366951	-0.982877
H	1.030176	4.597738	-1.974005
O	4.793833	3.868624	0.469281
H	4.960825	4.791116	0.249081
C	-0.121915	2.121407	-1.779313
C	-1.271892	2.298459	-0.774498

H	-0.058292	1.06638	-2.045886
H	-0.360514	2.674195	-2.688703
H	-1.048306	1.751965	0.138823
H	-1.384206	3.35056	-0.518265
C	-2.944639	0.532449	-1.180009
C	-4.285426	0.197531	-1.801064
C	-5.320637	-0.189005	-0.738222
H	-4.126595	-0.643422	-2.478237
H	-4.657715	1.0316	-2.396689
C	-6.657061	-0.598657	-1.352778
H	-4.92283	-1.00677	-0.135466
H	-5.474344	0.65565	-0.061753
C	-7.695436	-0.96983	-0.297632
H	-7.041248	0.216109	-1.969184
H	-6.50256	-1.449744	-2.022433
H	-7.301944	-1.788792	0.308579
H	-7.848164	-0.130801	0.383347
N	-2.545009	1.818137	-1.286435
H	-3.111283	2.450035	-1.825796
O	-2.2753	-0.319486	-0.589205
C	-10.596829	0.5907	-0.209998
C	-10.827603	-0.616396	0.711214
C	-9.031965	-1.413929	-0.888851
C	-10.093257	-1.867475	0.1416
C	-9.587851	-1.639081	2.422918
S	-9.919093	-0.070847	-1.784747
H	-10.799061	-2.519558	-0.364937
H	-11.894119	-0.81928	0.800586
N	-9.591857	-2.495011	1.352413
H	-8.950624	-3.269263	1.338463
N	-10.216973	-0.479413	2.03063
H	-10.655215	0.080115	2.743086
O	-9.109106	-1.864042	3.530417
H	-8.864165	-2.205839	-1.617069
H	-11.513103	1.127492	-0.433816
H	-9.885184	1.278779	0.237748

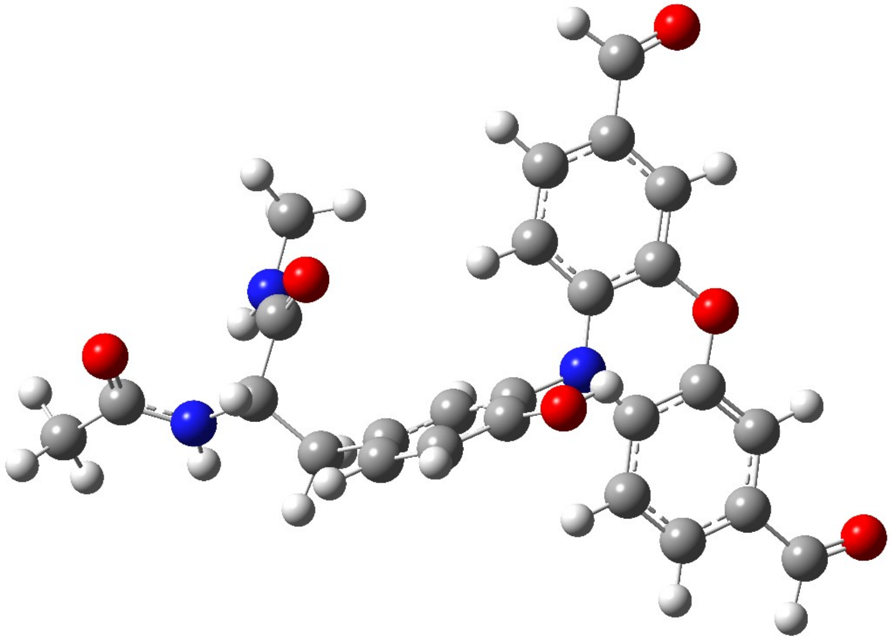

phenoxazine_plus_peptide-phenol_B3LYP-D3BJ_6-311_plus_2d2p_H2O
Zero-point correction= 0.450187 (Hartree/Particle)
Thermal correction to Energy= 0.482348
Thermal correction to Enthalpy= 0.483292
Thermal correction to Gibbs Free Energy= 0.383225
Sum of electronic and zero-point Energies= -1620.465580
Sum of electronic and thermal Energies= -1620.433419
Sum of electronic and thermal Enthalpies= -1620.432475
Sum of electronic and thermal Free Energies= -1620.532542

C	-1.624315	-2.104001	0.174977
C	-0.414674	-1.709121	-0.300417
C	0.84496	-1.806235	0.509679
C	0.578466	-2.120708	1.95787
C	-0.653177	-2.51069	2.393957
C	-1.753935	-2.540239	1.522165
H	-0.297095	-1.372234	-1.320508
H	-2.715947	-2.855713	1.896212
O	1.624908	-2.083405	2.824995
H	-0.774032	-2.777217	3.434681
C	-2.831463	-2.0969	-0.728149
C	-3.975871	-1.174185	-0.270061
H	-3.238437	-3.108055	-0.795976
H	-2.525163	-1.804449	-1.732425
H	-4.361971	-1.503333	0.692216
C	-6.262108	-0.706032	-1.044993
C	-7.286977	-0.916853	-2.132736
H	-7.615161	0.055951	-2.495973
C	-3.475888	0.258376	-0.032038
N	-3.363348	1.02645	-1.127605

H	-3.681305	0.648508	-2.003777
O	-3.167541	0.629274	1.09826
N	-5.037477	-1.245209	-1.261643
H	-4.895375	-1.809243	-2.082694
O	-6.513032	-0.070793	-0.022197
H	2.358285	-1.582314	2.448359
H	-6.909092	-1.500332	-2.968874
H	-8.151915	-1.421866	-1.705492
C	-2.878351	2.396084	-1.09644
H	-1.791205	2.434508	-1.152281
H	-3.297936	2.939205	-1.93861
H	-3.190875	2.87097	-0.170204
C	1.238875	0.666503	0.521234
C	3.053081	-0.78632	-0.102215
C	0.061613	0.931566	1.220696
C	1.958164	1.75345	-0.013362
C	3.72952	0.331183	-0.635313
C	3.754811	-1.993138	-0.01304
C	-0.375598	2.237402	1.382558
H	-0.529924	0.129222	1.623264
C	1.536381	3.047586	0.155054
C	5.025722	0.2452	-1.076382
H	3.296109	-2.867366	0.418778
C	5.06136	-2.086567	-0.470245
C	0.350873	3.304479	0.860496
H	-1.305648	2.415724	1.900771
H	2.108163	3.857224	-0.27319
C	5.709265	-0.977481	-1.007987
H	5.507014	1.119867	-1.487833
H	5.581904	-3.03125	-0.398026
N	1.732685	-0.62818	0.328436
O	3.089294	1.541364	-0.772618
C	-0.148399	4.665709	1.038241
H	-1.094318	4.73759	1.602298
C	7.086483	-1.106947	-1.480761
H	7.519085	-2.115757	-1.36687
O	7.748851	-0.205689	-1.968965
O	0.392998	5.675736	0.616748

H	1.425088	-2.635689	0.095325

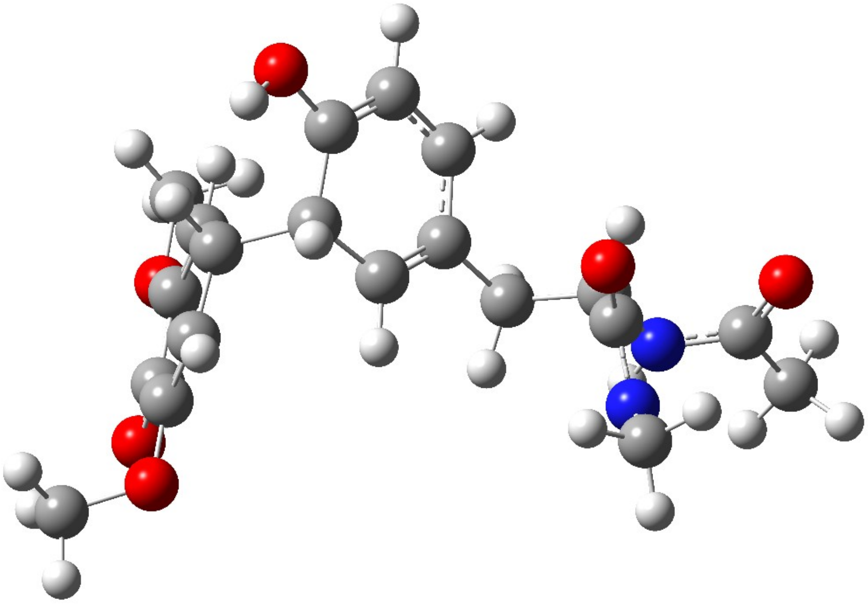

2-6-dimethoxyphenol-ketone_plus_peptide-phenol_rad_B3LYP-D3BJ_6-311_plus_2d2p_H2O_frozenbond

Zero-point correction= 0.429220 (Hartree/Particle)
Thermal correction to Energy= 0.459033
Thermal correction to Enthalpy= 0.459977
Thermal correction to Gibbs Free Energy= 0.363605
Sum of electronic and zero-point Energies= -1338.097691
Sum of electronic and thermal Energies= -1338.067879
Sum of electronic and thermal Enthalpies= -1338.066934
Sum of electronic and thermal Free Energies= -1338.163306

C	-0.552647	0.817333	0.349929
C	0.265097	0.581318	-0.70887
C	1.291784	1.562721	-1.16628
C	1.071201	2.920326	-0.59073
C	0.247365	3.132478	0.47776
C	-0.534194	2.085734	0.991271
H	0.247611	-0.378108	-1.205614
H	-1.167196	2.264558	1.848164
O	1.799157	3.962138	-1.084792
H	0.191406	4.122149	0.909618
C	-1.503354	-0.244551	0.836257
C	-2.983583	0.047611	0.521437
H	-1.415589	-0.346568	1.919589
H	-1.233987	-1.204779	0.396107
H	-3.287668	0.97795	0.996849
C	-5.139671	-0.971129	1.12047
C	-5.839984	-2.146232	1.758113
H	-6.514221	-2.589013	1.026241
C	-3.188215	0.273259	-0.982538

N	-3.345808	-0.831336	-1.728628
H	-3.455054	-1.712923	-1.257384
O	-3.148186	1.407009	-1.457658
N	-3.788144	-1.045408	1.043738
H	-3.319908	-1.824689	1.474983
O	-5.755796	-0.002481	0.678942
H	-5.154577	-2.90984	2.11776
H	-6.442672	-1.784905	2.589781
C	-3.530385	-0.775635	-3.168774
H	-2.701731	-0.247551	-3.636791
H	-3.567524	-1.790065	-3.553427
H	-4.456696	-0.262262	-3.427428
C	2.777001	1.03964	-0.787754
C	2.923775	0.926872	0.683037
C	3.05801	-0.204038	-1.53697
C	3.101131	-0.2516	1.30144
H	2.805176	1.836665	1.249621
C	3.250554	-1.384395	-0.93523
H	3.051483	-0.158419	-2.61817
C	3.217669	-1.515113	0.536645
H	2.218794	3.717819	-1.917272
O	3.276038	-2.607381	1.093856
O	3.389197	-2.528794	-1.674364
O	3.168824	-0.447437	2.640015
C	4.686823	-3.145444	-1.597467
H	4.936563	-3.401079	-0.57019
H	4.628679	-4.047908	-2.19826
H	5.441506	-2.47562	-2.012263
C	2.996152	0.701148	3.470481
H	3.053459	0.342129	4.492456
H	3.785676	1.432559	3.291709
H	2.023975	1.162924	3.292536
H	3.446267	1.822802	-1.155982
H	1.302846	1.625142	-2.259763

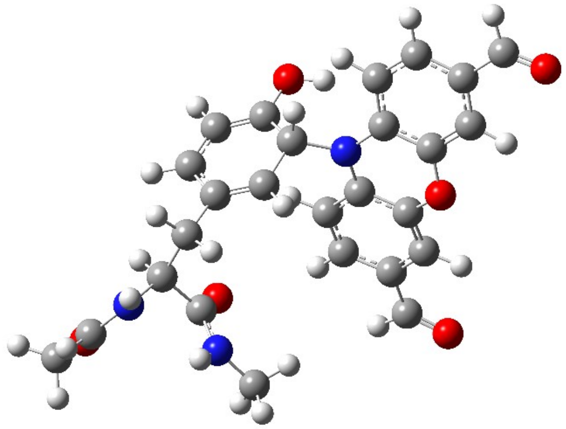

phenoxazine_plus_peptide-phenol_rad_B3LYP-D3BJ_6-311_plus_2d2p_H2O
Zero-point correction= 0.459656 (Hartree/Particle)
Thermal correction to Energy= 0.492301
Thermal correction to Enthalpy= 0.493245
Thermal correction to Gibbs Free Energy= 0.392000
Sum of electronic and zero-point Energies= -1620.998655
Sum of electronic and thermal Energies= -1620.966010
Sum of electronic and thermal Enthalpies= -1620.965065
Sum of electronic and thermal Free Energies= -1621.066311

C	-1.624315	-2.104001	0.174977
C	-0.414674	-1.709121	-0.300417
C	0.84496	-1.806235	0.509679
C	0.578466	-2.120708	1.95787
C	-0.653177	-2.51069	2.393957
C	-1.753935	-2.540239	1.522165
H	-0.297095	-1.372234	-1.320508
H	-2.715947	-2.855713	1.896212
O	1.624908	-2.083405	2.824995
H	-0.774032	-2.777217	3.434681
C	-2.831463	-2.0969	-0.728149
C	-3.975871	-1.174185	-0.270061
H	-3.238437	-3.108055	-0.795976
H	-2.525163	-1.804449	-1.732425
H	-4.361971	-1.503333	0.692216
C	-6.262108	-0.706032	-1.044993
C	-7.286977	-0.916853	-2.132736
H	-7.615161	0.055951	-2.495973
C	-3.475888	0.258376	-0.032038
N	-3.363348	1.02645	-1.127605
H	-3.681305	0.648508	-2.003777
O	-3.167541	0.629274	1.09826
N	-5.037477	-1.245209	-1.261643
H	-4.895375	-1.809243	-2.082694
O	-6.513032	-0.070793	-0.022197
H	2.358285	-1.582314	2.448359
H	-6.909092	-1.500332	-2.968874
H	-8.151915	-1.421866	-1.705492
C	-2.878351	2.396084	-1.09644
H	-1.791205	2.434508	-1.152281
H	-3.297936	2.939205	-1.93861
H	-3.190875	2.87097	-0.170204
C	1.238875	0.666503	0.521234
C	3.053081	-0.78632	-0.102215
C	0.061613	0.931566	1.220696
C	1.958164	1.75345	-0.013362
C	3.72952	0.331183	-0.635313

C	3.754811	-1.993138	-0.01304
C	-0.375598	2.237402	1.382558
H	-0.529924	0.129222	1.623264
C	1.536381	3.047586	0.155054
C	5.025722	0.2452	-1.076382
H	3.296109	-2.867366	0.418778
C	5.06136	-2.086567	-0.470245
C	0.350873	3.304479	0.860496
H	-1.305648	2.415724	1.900771
H	2.108163	3.857224	-0.27319
C	5.709265	-0.977481	-1.007987
H	5.507014	1.119867	-1.487833
H	5.581904	-3.03125	-0.398026
N	1.732685	-0.62818	0.328436
O	3.089294	1.541364	-0.772618
C	-0.148399	4.665709	1.038241
H	-1.094318	4.73759	1.602298
C	7.086483	-1.106947	-1.480761
H	7.519085	-2.115757	-1.36687
O	7.748851	-0.205689	-1.968965
O	0.392998	5.675736	0.616748
H	1.425088	-2.635689	0.095325

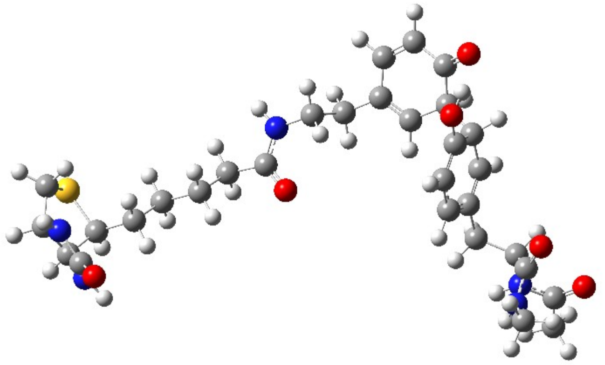

peptide-phenol_rad_plus_tag-ketone_B3LYP-D3BJ_6-311_plus_2d2p_H2O_3_frozenbond
Zero-point correction= 0.680488 (Hartree/Particle)
Thermal correction to Energy= 0.723880
Thermal correction to Enthalpy= 0.724824
Thermal correction to Gibbs Free Energy= 0.590281
Sum of electronic and zero-point Energies= -2290.764676
Sum of electronic and thermal Energies= -2290.721284
Sum of electronic and thermal Enthalpies= -2290.720340
Sum of electronic and thermal Free Energies= -2290.854883

C	4.433481	-0.254664	-0.098972
C	3.488538	-0.383422	0.951578
C	3.372804	0.613216	1.937096
C	4.111006	1.757337	1.855644
C	4.966976	2.073978	0.670013
C	5.165526	0.887185	-0.216472

H	2.893592	-1.281439	1.02854
H	5.886796	0.994246	-1.016432
O	4.023897	2.687473	2.84431
H	2.714257	0.472463	2.783197
C	4.640657	-1.404544	-1.052561
C	5.754167	-2.381818	-0.623602
H	4.904112	-1.027394	-2.04057
H	3.708655	-1.962683	-1.154632
H	6.708946	-1.859804	-0.612107
C	6.821494	-4.366412	-1.61049
C	6.78786	-5.389537	-2.719631
H	6.759422	-6.383878	-2.276488
C	5.546732	-2.88047	0.813429
N	4.683822	-3.897544	0.963761
H	4.326548	-4.347303	0.138441
O	6.107834	-2.328904	1.758795
N	5.806946	-3.467704	-1.590631
H	5.135682	-3.47824	-2.340379
O	7.711072	-4.351062	-0.761098
H	5.936196	-5.271266	-3.385022
H	7.707312	-5.308845	-3.297479
C	4.360984	-4.455818	2.26558
H	3.968904	-3.682107	2.923478
H	3.607883	-5.226797	2.135451
H	5.242041	-4.893686	2.73475
C	4.364982	3.283172	-0.16316
C	3.059865	2.95352	-0.800487
C	4.400631	4.590825	0.610422
C	2.033235	3.81611	-0.854956
H	2.971862	1.969547	-1.240651
C	3.307062	5.515589	0.402362
C	2.194496	5.132093	-0.262781
H	3.383255	6.488809	0.865673
H	1.368612	5.827633	-0.348606
O	5.328989	4.835915	1.391636
C	0.704524	3.451249	-1.459055
C	-0.32244	3.101235	-0.369522
H	0.818158	2.593747	-2.119661
H	0.320216	4.280484	-2.055901
H	0.043263	2.257096	0.210979
H	-0.45878	3.94105	0.309679
C	-1.931236	1.502605	-1.332272
C	-3.332633	1.311547	-1.874352
C	-4.185142	0.451363	-0.933174
H	-3.242567	0.815627	-2.841897
H	-3.818821	2.273145	-2.041889
C	-5.59075	0.211587	-1.479071
H	-3.682022	-0.503088	-0.770616

H	-4.252344	0.942297	0.040985
C	-6.440152	-0.646028	-0.54496
H	-6.086566	1.169906	-1.644242
H	-5.522943	-0.276938	-2.455411
H	-5.941558	-1.607202	-0.402203
H	-6.494114	-0.179849	0.440294
N	-1.62208	2.749859	-0.914891
H	-2.299816	3.480533	-1.047174
O	-1.134536	0.564361	-1.248997
C	-9.316575	0.466486	0.620606
C	-9.367489	-1.033431	0.947462
C	-7.847619	-0.910869	-1.075873
C	-8.717698	-1.851465	-0.208673
C	-7.842076	-2.576374	1.844813
S	-8.892375	0.603743	-1.162472
H	-9.486956	-2.281157	-0.843815
H	-10.399947	-1.349211	1.09242
N	-8.014157	-2.891859	0.521915
H	-7.371946	-3.52642	0.079466
N	-8.545157	-1.419062	2.091049
H	-8.864108	-1.255564	3.031386
O	-7.180186	-3.209209	2.661935
H	-7.788186	-1.298223	-2.091761
H	-10.26858	0.961976	0.781726
H	-8.556265	0.959705	1.219763
H	5.933013	2.449892	1.01949
H	5.090204	3.43529	-0.974497
H	4.635148	3.417868	2.660544

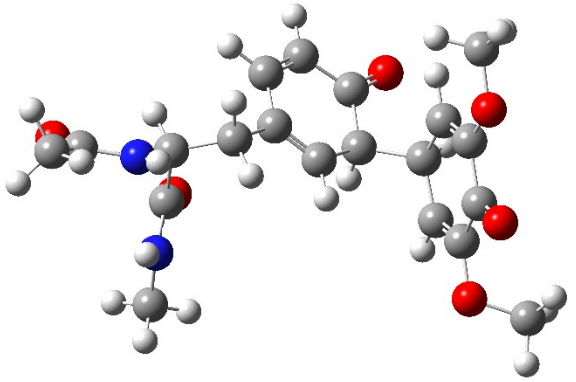

2-6-dimethoxyphenol-ketone_plus_peptide-ketone_B3LYP-D3BJ_6-311_plus_2d2p_H2O
Zero-point correction= 0.419352 (Hartree/Particle)
Thermal correction to Energy= 0.448597
Thermal correction to Enthalpy= 0.449541
Thermal correction to Gibbs Free Energy= 0.354378
Sum of electronic and zero-point Energies= -1337.527794
Sum of electronic and thermal Energies= -1337.498550

Sum of electronic and thermal Enthalpies= -1337.497606
Sum of electronic and thermal Free Energies= -1337.592769

C	0.788274	-1.042764	0.084655
C	-0.188351	-0.385291	-0.551796
C	-1.157937	-1.056327	-1.466155
C	-1.013176	-2.571768	-1.591065
C	0.082243	-3.203116	-0.875899
C	0.911593	-2.48084	-0.096189
H	-0.299466	0.68331	-0.423225
H	1.703894	-2.988945	0.439261
O	-1.806598	-3.211774	-2.276291
H	0.186287	-4.274127	-0.97425
C	1.766924	-0.343992	0.98596
C	3.198225	-0.282128	0.415413
H	1.819179	-0.862629	1.945415
H	1.419409	0.670546	1.179468
H	3.581989	-1.288702	0.25859
C	5.399771	0.397089	1.265481
C	6.162134	1.075784	2.376784
H	6.80932	1.836293	1.943244
C	3.193747	0.368409	-0.974136
N	3.286664	1.70584	-1.003773
H	3.483245	2.188824	-0.143782
O	3.047546	-0.32328	-1.980952
N	4.046985	0.408505	1.370505
H	3.627938	0.793448	2.200338
O	5.963927	-0.131087	0.309326
H	5.515276	1.536847	3.11923
H	6.796632	0.337697	2.865701
C	3.269165	2.458996	-2.246667
H	2.371061	2.228084	-2.81618
H	3.277718	3.518315	-2.010096
H	4.138216	2.223775	-2.861254
C	-2.643692	-0.692934	-1.10732
C	-2.969869	-1.144879	0.274229
C	-2.910383	0.742031	-1.368516
C	-3.396184	-0.305819	1.231341
H	-2.822286	-2.193243	0.484091
C	-3.349812	1.58608	-0.428718
H	-2.720134	1.116698	-2.365766
C	-3.585794	1.141446	0.963057
O	-3.909004	1.924967	1.847239
O	-3.509782	2.916516	-0.704383
O	-3.682268	-0.633569	2.51178
C	-4.875648	3.370643	-0.714494
H	-5.355584	3.181791	0.243149
H	-4.837164	4.438955	-0.903522

H	-5.425536	2.874322	-1.515263
C	-3.491759	-1.997245	2.891002
H	-3.756336	-2.054253	3.941255
H	-4.139568	-2.655431	2.310496
H	-2.451027	-2.293983	2.753894
H	-3.239537	-1.280331	-1.814953
H	-0.986468	-0.672103	-2.478518

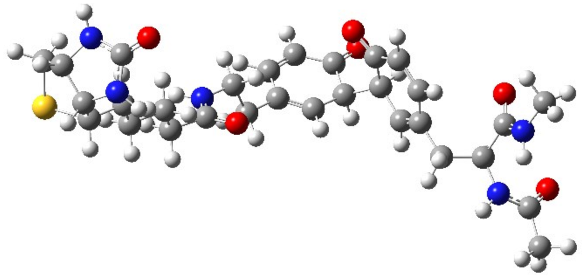

peptide-ketone_plus_tag-ketone_B3LYP-D3BJ_6-311_plus_2d2p_H2O
Zero-point correction= 0.670238 (Hartree/Particle)
Thermal correction to Energy= 0.713268
Thermal correction to Enthalpy= 0.714213
Thermal correction to Gibbs Free Energy= 0.582086
Sum of electronic and zero-point Energies= -2290.193241
Sum of electronic and thermal Energies= -2290.150210
Sum of electronic and thermal Enthalpies= -2290.149266
Sum of electronic and thermal Free Energies= -2290.281393

C	4.763818	-0.288761	-0.105642
C	4.014603	-0.698432	1.072121
C	3.469354	0.174134	1.943672
C	3.574449	1.607443	1.730986
C	4.493477	2.079508	0.609836
C	4.961669	1.017997	-0.327859
H	3.871479	-1.759396	1.232022
H	5.516781	1.345724	-1.198094
O	2.960219	2.421022	2.415853
H	2.877607	-0.160803	2.783567
C	5.291768	-1.345469	-1.039979
C	6.666616	-1.917189	-0.636032
H	5.398155	-0.925529	-2.039207
H	4.575906	-2.16702	-1.106379
H	7.402772	-1.115714	-0.648692
C	8.312918	-3.40646	-1.686588
C	8.588866	-4.390069	-2.79712
H	8.914791	-5.33123	-2.356468
C	6.661631	-2.438461	0.80697
N	6.301206	-3.718596	0.977361
H	6.178884	-4.29183	0.160235

O	6.919227	-1.679586	1.74111
N	7.044221	-2.932126	-1.605071
H	6.383245	-3.189639	-2.318682
O	9.179342	-3.059934	-0.886018
H	7.724183	-4.573871	-3.430173
H	9.405469	-4.007875	-3.40748
C	6.232325	-4.337612	2.290595
H	5.549397	-3.786914	2.934795
H	5.87067	-5.354498	2.175323
H	7.213233	-4.358868	2.765195
C	3.893557	3.309625	-0.115244
C	2.56317	3.0056	-0.724325
C	3.952064	4.551258	0.769953
C	1.504994	3.819053	-0.609208
H	2.486204	2.084236	-1.288523
C	2.773854	5.403693	0.798053
C	1.642568	5.050156	0.156773
H	2.837153	6.31627	1.37356
H	0.780735	5.704132	0.211911
O	4.982548	4.831755	1.376637
C	0.162418	3.489036	-1.201966
C	-0.825108	3.010618	-0.125247
H	0.267793	2.706466	-1.951008
H	-0.254568	4.367807	-1.698108
H	-0.431474	2.116735	0.353475
H	-0.953565	3.772185	0.641843
C	-2.433106	1.500604	-1.221365
C	-3.84424	1.343298	-1.748642
C	-4.652954	0.352483	-0.902273
H	-3.768703	0.972994	-2.772128
H	-4.355567	2.305747	-1.784241
C	-6.062274	0.136817	-1.448855
H	-4.120608	-0.599207	-0.863549
H	-4.71332	0.722131	0.124483
C	-6.87076	-0.842836	-0.602699
H	-6.585992	1.093288	-1.497905
H	-6.000319	-0.237851	-2.474677
H	-6.338044	-1.795608	-0.565666
H	-6.928401	-0.483843	0.426255
N	-2.135475	2.69684	-0.668643
H	-2.827904	3.425032	-0.701214
O	-1.616961	0.57663	-1.268506
C	-9.77376	0.04645	0.689095
C	-9.767763	-1.479307	0.865687
C	-8.274432	-1.101927	-1.146096
C	-9.100909	-2.154742	-0.369977
C	-8.176812	-3.044412	1.595578
S	-9.371945	0.375498	-1.073962

H	-9.860697	-2.548336	-1.039005
H	-10.786712	-1.845395	0.985484
N	-8.351661	-3.235181	0.249062
H	-7.686474	-3.793179	-0.258123
N	-8.920609	-1.945508	1.959913
H	-9.234343	-1.88661	2.914201
O	-7.482038	-3.72816	2.34124
H	-8.213062	-1.384422	-2.195918
H	-10.741296	0.488439	0.904108
H	-9.026057	0.505274	1.329939
H	5.38695	2.457838	1.128247
H	4.582345	3.540999	-0.938112

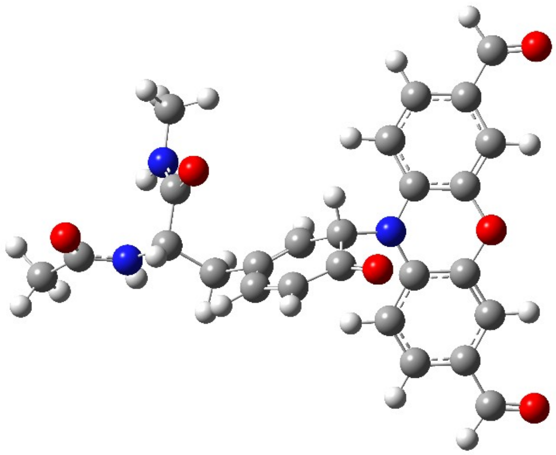

phenoxazine_plus_peptide-ketone_B3LYP-D3BJ_6-311_plus_2d2p_H2O
Zero-point correction= 0.449319 (Hartree/Particle)
Thermal correction to Energy= 0.481601
Thermal correction to Enthalpy= 0.482545
Thermal correction to Gibbs Free Energy= 0.380899
Sum of electronic and zero-point Energies= -1620.426744
Sum of electronic and thermal Energies= -1620.394462
Sum of electronic and thermal Enthalpies= -1620.393518
Sum of electronic and thermal Free Energies= -1620.495164

C	-1.9012	-1.262956	0.427637
C	-0.8464	-0.493208	0.133487
C	0.235793	-0.229122	1.133329
C	0.288478	-1.184098	2.341472
C	-0.932084	-1.930576	2.601191
C	-1.939035	-1.960409	1.704132
H	-0.776152	0.030371	-0.810087
H	-2.809396	-2.567878	1.91665
O	1.281068	-1.242927	3.048699
H	-0.964289	-2.519458	3.506311
C	-3.057085	-1.418475	-0.519172
C	-4.310429	-0.637234	-0.071441

H	-3.33361	-2.470397	-0.604928
H	-2.761054	-1.072294	-1.508905
H	-4.680234	-1.035379	0.871709
C	-6.625748	-0.428362	-0.857003
C	-7.606744	-0.707029	-1.968975
H	-8.095409	0.225956	-2.244849
C	-3.949935	0.825534	0.223428
N	-3.981153	1.667579	-0.818899
H	-4.356656	1.334235	-1.690269
O	-3.591231	1.157144	1.352477
N	-5.336597	-0.785439	-1.087609
H	-5.111627	-1.274162	-1.937955
O	-6.963333	0.103143	0.198724
H	-7.142274	-1.141611	-2.850783
H	-8.37232	-1.38654	-1.596997
C	-3.624585	3.071299	-0.694729
H	-2.61313	3.172698	-0.305793
H	-3.675374	3.529564	-1.677446
H	-4.307195	3.591017	-0.022665
C	2.021079	1.278807	0.306775
C	2.277274	-1.094699	0.070364
C	1.222819	2.422046	0.386155
C	3.376784	1.434802	-0.048968
C	3.624443	-0.893814	-0.286163
C	1.735387	-2.372997	-0.068162
C	1.769608	3.676519	0.156857
H	0.172146	2.344992	0.612632
C	3.916929	2.671763	-0.289824
C	4.400023	-1.920486	-0.760366
H	0.702653	-2.557216	0.178514
C	2.51951	-3.417876	-0.534335
C	3.114263	3.817575	-0.178117
H	1.139536	4.551838	0.230248
H	4.961408	2.75792	-0.549624
C	3.851993	-3.20683	-0.883689
H	5.43189	-1.737379	-1.020278
H	2.088777	-4.404627	-0.631232
N	1.547938	-0.009073	0.550396
O	4.212681	0.340775	-0.114589
C	3.66022	5.152622	-0.414924
H	2.931283	5.97395	-0.305919
C	4.651837	-4.327465	-1.374497
H	4.111825	-5.288167	-1.431696
O	5.823958	-4.260879	-1.708237
O	4.819016	5.394826	-0.711659
H	-0.048543	0.702428	1.636054

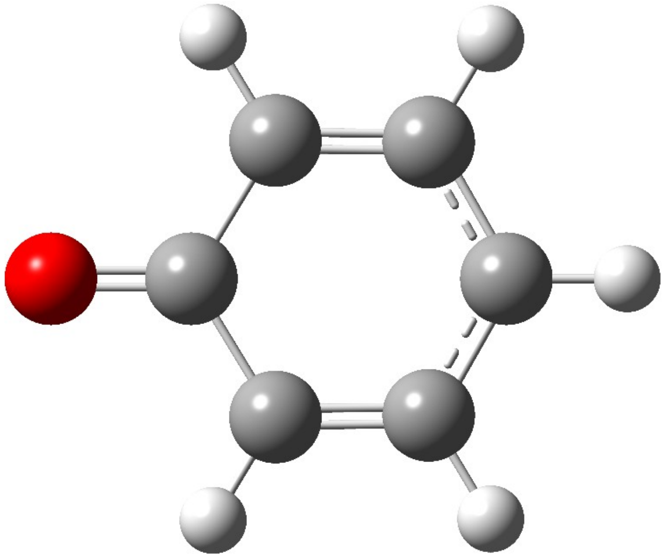

phenoxyradical_B3LYP-D3BJ_6-311_plus_2d2p_H2O
Zero-point correction= 0.091477 (Hartree/Particle)
Thermal correction to Energy= 0.096772
Thermal correction to Enthalpy= 0.097716
Thermal correction to Gibbs Free Energy= 0.061915
Sum of electronic and zero-point Energies= -306.860840
Sum of electronic and thermal Energies= -306.855545
Sum of electronic and thermal Enthalpies= -306.854601
Sum of electronic and thermal Free Energies= -306.890403

C	1.777357	-0.000001	-0.000002
C	1.082139	1.222767	0.000045
C	-0.288457	1.237542	-0.00002
C	-1.040496	-0.000003	-0.000078
C	-0.288458	-1.237542	0.000022
C	1.08214	-1.222764	-0.000013
H	2.857656	0.000005	-0.000017
H	1.637334	2.149756	-0.000005
H	-0.847577	2.162217	-0.00001
H	-0.847565	-2.162226	0.000038
H	1.637332	-2.149755	0.000005
O	-2.297816	0.000002	0.000033

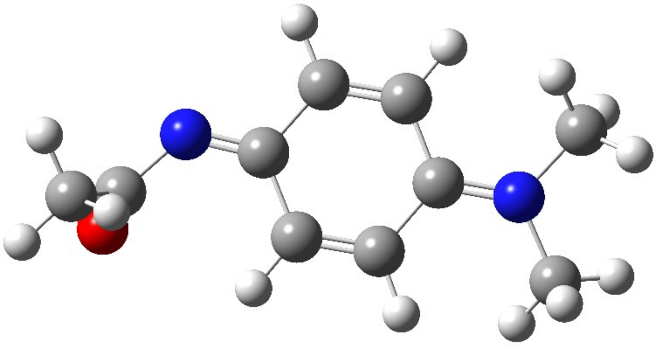

dimethyliminium_probe_B3LYP-D3BJ_6-311_plus_2d2p_H2O
Zero-point correction= 0.216460 (Hartree/Particle)
Thermal correction to Energy= 0.229938
Thermal correction to Enthalpy= 0.230882
Thermal correction to Gibbs Free Energy= 0.175266
Sum of electronic and zero-point Energies= -573.435475
Sum of electronic and thermal Energies= -573.421997
Sum of electronic and thermal Enthalpies= -573.421053
Sum of electronic and thermal Free Energies= -573.476670

C	-0.111662	1.610371	-0.153832
C	-1.083609	0.52364	-0.096525
C	-0.560432	-0.837472	0.011353
C	0.758456	-1.073158	0.048577
C	1.715331	0.021443	0.000721
C	1.207652	1.378275	-0.098564
H	-0.499076	2.613029	-0.252108
H	-1.25914	-1.65823	0.07005
H	1.113078	-2.083331	0.155298
H	1.896752	2.201422	-0.168538
N	3.004405	-0.213341	0.048843
N	-2.329879	0.820813	-0.137166
C	4.009072	0.8556	0.160027
H	4.863824	0.448942	0.689441
H	4.32015	1.167859	-0.835592
H	3.62167	1.697835	0.717184
C	3.579949	-1.566488	-0.00434
H	3.700901	-1.95053	1.007272
H	2.960407	-2.231755	-0.590379
H	4.553902	-1.48997	-0.475513
C	-3.368174	-0.117423	-0.146002
O	-3.675968	-0.686209	-1.175119
C	-4.104624	-0.242672	1.15581
H	-4.905484	-0.968294	1.052248
H	-3.418952	-0.549072	1.946402
H	-4.513713	0.726773	1.440087

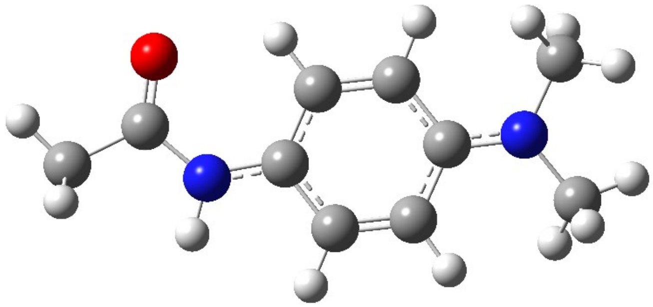

dimethylamine_rad_probe_B3LYP-D3BJ_6-311_plus_2d2p_H2O
Zero-point correction= 0.228600 (Hartree/Particle)
Thermal correction to Energy= 0.242254
Thermal correction to Enthalpy= 0.243198
Thermal correction to Gibbs Free Energy= 0.186480
Sum of electronic and zero-point Energies= -574.065864
Sum of electronic and thermal Energies= -574.052210
Sum of electronic and thermal Enthalpies= -574.051265
Sum of electronic and thermal Free Energies= -574.107983

C	-0.126138	-1.496907	0.03634
C	-0.993011	-0.376357	0.012705
C	-0.43139	0.921106	-0.010884
C	0.926723	1.081705	-0.011927
C	1.807544	-0.038598	0.004797
C	1.228012	-1.341993	0.03061
H	-0.54828	-2.491343	0.062146
H	-1.078928	1.778119	-0.031917
H	1.325532	2.081368	-0.040656
H	1.85396	-2.217355	0.0589
N	3.143468	0.127383	-0.004874
N	-2.343113	-0.626179	0.013266
C	4.049694	-1.020652	-0.073411
H	5.052008	-0.659767	-0.264896
H	4.042052	-1.565373	0.870363
H	3.755645	-1.688055	-0.87885
C	3.741903	1.462652	0.05293
H	3.568449	1.995463	-0.881667
H	3.320359	2.031436	0.877351
H	4.807999	1.358058	0.207224
C	-3.411358	0.270882	-0.006912
O	-3.253723	1.475838	-0.022695
C	-4.758778	-0.40007	-0.009217
H	-5.532131	0.359853	-0.005895
H	-4.868304	-1.027565	-0.894244
H	-4.869717	-1.038456	0.867552
H	-2.600555	-1.602118	0.02721

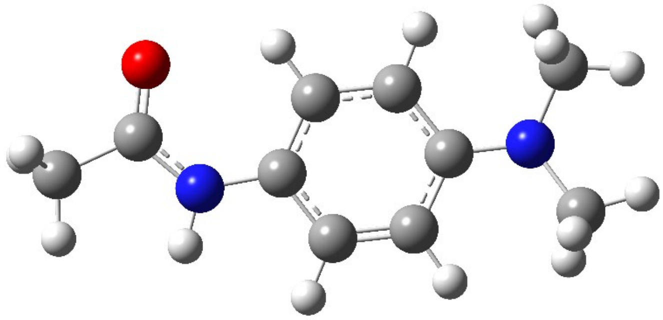

dimethylamine_probe_B3LYP-D3BJ_6-311_plus_2d2p_H2O
Zero-point correction= 0.227746 (Hartree/Particle)
Thermal correction to Energy= 0.241223
Thermal correction to Enthalpy= 0.242168
Thermal correction to Gibbs Free Energy= 0.186422
Sum of electronic and zero-point Energies= -574.246906
Sum of electronic and thermal Energies= -574.233428
Sum of electronic and thermal Enthalpies= -574.232484
Sum of electronic and thermal Free Energies= -574.288230

C	-0.134116	-1.473064	-0.034886
C	-0.992987	-0.372031	-0.014331
C	-0.427745	0.903595	-0.022028
C	0.951512	1.063326	-0.057632
C	1.828825	-0.036142	-0.099139
C	1.24066	-1.31671	-0.069536
H	-0.546207	-2.473901	-0.016522
H	-1.06582	1.769057	0.005313
H	1.339666	2.068638	-0.054678
H	1.853714	-2.202679	-0.076
N	3.204949	0.129834	-0.185831
N	-2.38271	-0.627944	0.029566
C	4.058802	-1.010299	0.111673
H	5.095232	-0.715279	-0.02137
H	3.930154	-1.377111	1.137329
H	3.860571	-1.833541	-0.572622
C	3.762363	1.439727	0.115097
H	3.371871	2.193819	-0.566233
H	3.550851	1.762024	1.141978
H	4.839123	1.401184	-0.019439
C	-3.423739	0.245659	0.016155
O	-3.289646	1.468189	-0.035972
C	-4.79521	-0.39026	0.071602
H	-5.358267	-0.081318	-0.80812
H	-4.770983	-1.476637	0.113494
H	-5.318727	-0.012993	0.94912
H	-2.629879	-1.602816	0.067533

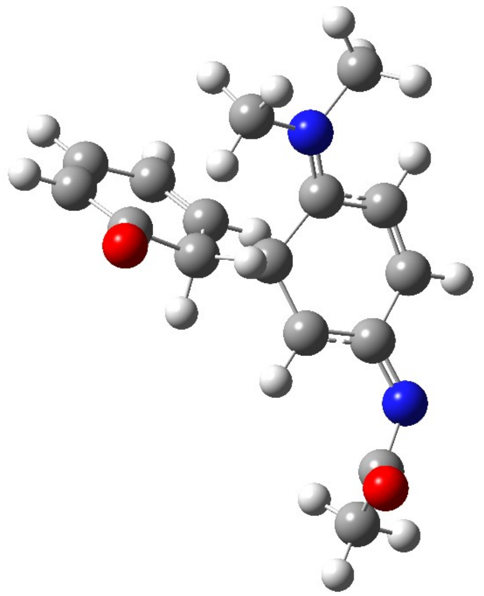

dimethyliminium_probe_C-rad_plus_phenol-ketone_B3LYP-D3BJ_6-311_plus_2d2p_H2O
Zero-point correction= 0.310974 (Hartree/Particle)
Thermal correction to Energy= 0.330354
Thermal correction to Enthalpy= 0.331298
Thermal correction to Gibbs Free Energy= 0.261387
Sum of electronic and zero-point Energies= -880.281524
Sum of electronic and thermal Energies= -880.262144
Sum of electronic and thermal Enthalpies= -880.261200
Sum of electronic and thermal Free Energies= -880.331111

C	0.10853	0.079983	-0.74372
C	-1.368541	-0.030845	-0.635076
C	0.672689	1.283399	-0.026593
C	-2.108344	0.634995	0.347491
C	-0.07387	1.810078	1.092664
C	-1.375573	1.517034	1.253886
H	0.400224	2.493358	1.775379
H	-1.934053	1.953633	2.068706
H	0.374547	0.083047	-1.798398
H	-1.849453	-0.726366	-1.306145
N	-3.404561	0.507496	0.547132
N	1.801433	1.836422	-0.370575
C	2.597784	1.481747	-1.552127
H	2.802031	2.399987	-2.096014
H	3.538286	1.047456	-1.220793
H	2.083282	0.783912	-2.193679
C	2.380696	2.95106	0.410944
H	3.348106	3.187597	-0.012121
H	1.734676	3.822359	0.34819
H	2.51126	2.650916	1.445684
C	-4.22317	-0.248654	-0.290448
O	-4.555742	0.165091	-1.387191

C	-4.727624	-1.525818	0.319261
H	-3.886538	-2.155179	0.612391
H	-5.294033	-1.301792	1.22313
H	-5.357191	-2.050642	-0.392909
C	0.744255	-1.265959	-0.167235
C	2.071088	-1.580146	-0.871686
C	0.767188	-1.298015	1.325852
C	3.230281	-1.834868	-0.0397
C	1.892271	-1.550731	2.003002
H	-0.163861	-1.126896	1.846441
C	3.138129	-1.804029	1.307412
H	4.165108	-2.033867	-0.542977
H	1.889415	-1.577704	3.082541
H	4.022749	-1.982728	1.903572
O	2.107334	-1.606885	-2.096722
H	0.069854	-2.055554	-0.51115

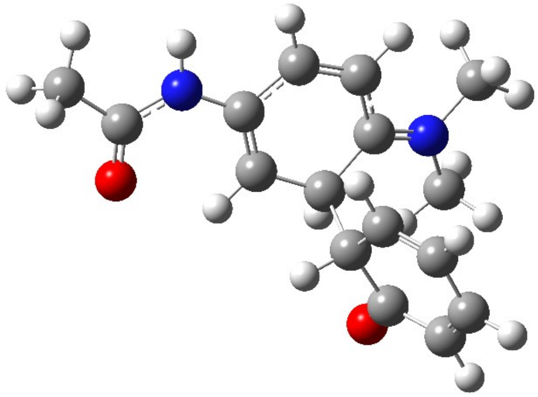

dimethyliminium_probe_plus_phenol-ketone_B3LYP-D3BJ_6-311_plus_2d2p_H2O
Zero-point correction= 0.325163 (Hartree/Particle)
Thermal correction to Energy= 0.344632
Thermal correction to Enthalpy= 0.345576
Thermal correction to Gibbs Free Energy= 0.275667
Sum of electronic and zero-point Energies= -880.933112
Sum of electronic and thermal Energies= -880.913643
Sum of electronic and thermal Enthalpies= -880.912699
Sum of electronic and thermal Free Energies= -880.982608

C	0.188332	-0.02554	-0.759532
C	-1.290171	-0.205517	-0.656162
C	0.705154	1.283303	-0.217038
C	-2.054204	0.571766	0.133942
C	-0.119137	1.972081	0.731222
C	-1.420441	1.639025	0.875743
H	0.271795	2.802215	1.292142
H	-2.022459	2.211554	1.568671
H	0.47537	-0.158056	-1.801065
H	-1.710989	-1.040127	-1.187605
N	-3.435056	0.439172	0.331456

N	1.868183	1.779064	-0.561183
C	2.723386	1.259478	-1.632049
H	3.018886	2.099614	-2.254676
H	3.61349	0.811106	-1.195736
H	2.208574	0.526433	-2.234424
C	2.405726	2.987812	0.092434
H	3.425603	3.124925	-0.243783
H	1.814939	3.857621	-0.185131
H	2.40576	2.863123	1.170579
C	-4.273532	-0.494758	-0.21814
O	-3.883053	-1.359079	-0.992406
C	-5.717163	-0.391869	0.208958
H	-5.93579	0.501466	0.788485
H	-6.344454	-0.407601	-0.679871
H	-5.963368	-1.269154	0.806734
H	-3.857047	1.108763	0.953105
C	0.863973	-1.247386	0.009805
C	2.193479	-1.654234	-0.634081
C	0.882226	-1.044045	1.488767
C	3.350747	-1.77452	0.230867
C	2.005249	-1.183256	2.20205
H	-0.052982	-0.789233	1.96654
C	3.252713	-1.541061	1.557988
H	4.287838	-2.049125	-0.231195
H	2.000483	-1.03544	3.271811
H	4.13526	-1.627451	2.177498
H	0.204961	-2.094428	-0.201448
O	2.234831	-1.874863	-1.84024

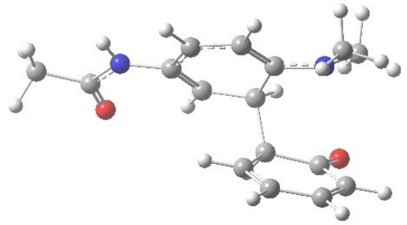

dimethylamine_probe_C-rad_plus_phenol-ketone_B3LYP-D3BJ_6-311_plus_2d2p_H2O_frozenbond_2

Zero-point correction= 0.321733 (Hartree/Particle)
Thermal correction to Energy= 0.341424
Thermal correction to Enthalpy= 0.342368
Thermal correction to Gibbs Free Energy= 0.271112
Sum of electronic and zero-point Energies= -881.077308
Sum of electronic and thermal Energies= -881.057617
Sum of electronic and thermal Enthalpies= -881.056673
Sum of electronic and thermal Free Energies= -881.127929

C	0.185248	0.005178	-0.847405

C	-1.30981	-0.087708	-0.78057
C	0.746433	1.232678	-0.163666
C	-2.029273	0.631383	0.120796
C	-0.036051	1.910283	0.750992
C	-1.38541	1.606852	0.937542
H	0.386186	2.720521	1.324874
H	-1.965398	2.168156	1.656454
H	0.478556	-0.019655	-1.898374
H	-1.790043	-0.819568	-1.405382
N	-3.426254	0.514488	0.306033
N	2.094048	1.483761	-0.329668
C	2.64776	1.606047	-1.677792
H	2.523906	2.627716	-2.053489
H	3.709694	1.369592	-1.652502
H	2.167352	0.918513	-2.361775
C	2.767615	2.339264	0.63338
H	3.841496	2.229354	0.498507
H	2.514021	3.39831	0.507835
H	2.512388	2.032373	1.644522
C	-4.292489	-0.393937	-0.221069
O	-3.956857	-1.281719	-1.001505
C	-5.731663	-0.237477	0.218262
H	-5.880667	0.550445	0.952723
H	-6.341881	-0.023988	-0.658616
H	-6.07157	-1.183611	0.636253
H	-3.825888	1.187728	0.938001
C	0.817492	-1.264506	-0.247597
C	2.256242	-1.447597	-0.720355
C	0.623079	-1.349929	1.231464
C	3.303786	-1.549463	0.281001
C	1.645943	-1.491852	2.083768
H	-0.391504	-1.264294	1.594922
C	3.007219	-1.581598	1.59786
H	4.321613	-1.625277	-0.075166
H	1.473591	-1.536605	3.14975
H	3.804218	-1.677599	2.323517
H	0.267688	-2.093622	-0.705087
O	2.493726	-1.532274	-1.927394

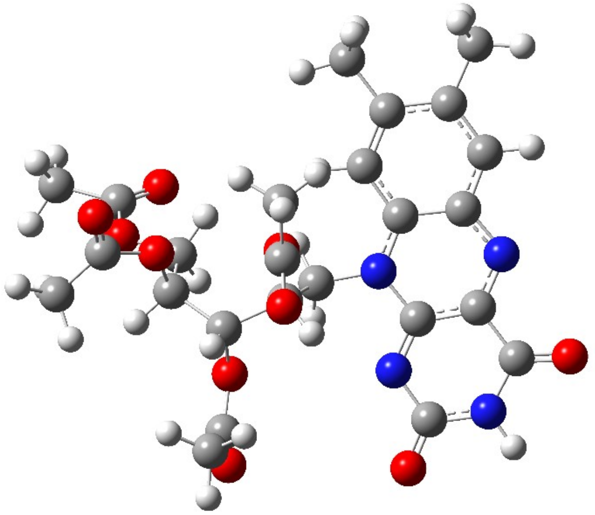

RFT_triplet_B3LYP-D3BJ_6-311_plus_2d2p_H2O_maxdisk8GB_2
Zero-point correction= 0.519245 (Hartree/Particle)
Thermal correction to Energy= 0.558682
Thermal correction to Enthalpy= 0.559626
Thermal correction to Gibbs Free Energy= 0.440269
Sum of electronic and zero-point Energies= -1941.068660
Sum of electronic and thermal Energies= -1941.029224
Sum of electronic and thermal Enthalpies= -1941.028280
Sum of electronic and thermal Free Energies= -1941.147636

C	5.069392	-1.520901	-0.099751
C	4.077586	-0.457954	-0.210953
C	2.743244	-0.860342	-0.449085
C	3.230815	-3.135208	-0.446879
C	2.093263	1.449462	-0.364162
C	3.489498	1.762021	-0.138848
C	3.822777	3.103703	0.032326
H	4.864694	3.339424	0.191464
C	2.874575	4.123729	0.013235
C	1.495878	3.802852	-0.186223
C	1.141288	2.486302	-0.371979
H	5.208028	-3.567958	-0.14934
H	0.099371	2.254677	-0.499474
N	1.751714	0.13533	-0.567685
N	4.473382	0.824346	-0.081248
N	2.319775	-2.099957	-0.565091
N	4.552011	-2.80396	-0.228937
C	0.450877	4.878058	-0.185377
H	0.442038	5.41771	0.763164
H	0.641372	5.614943	-0.967402
H	-0.538296	4.457464	-0.346851
C	3.296833	5.541736	0.206462
H	3.00679	6.151192	-0.652225

H	2.79783	5.97806	1.074557
H	4.371102	5.621929	0.344565
O	2.87979	-4.307055	-0.534114
O	6.2696	-1.351499	0.093294
C	0.380087	-0.29724	-0.850076
C	-0.325127	-0.725978	0.434212
O	-0.641276	0.472803	1.156271
C	-0.744552	0.384037	2.505373
O	-0.57373	-0.649307	3.105888
H	0.371849	-1.309467	1.027089
H	0.433985	-1.137318	-1.527486
H	-0.138734	0.507985	-1.352043
C	-1.106106	1.709623	3.09589
H	-0.470848	2.49244	2.686137
H	-2.136772	1.936359	2.822209
H	-1.015992	1.670329	4.175938
C	-1.591026	-1.587421	0.230142
H	-1.760575	-2.144502	1.145771
O	-1.388645	-2.477848	-0.876732
C	-0.859297	-3.727009	-0.735808
O	-0.734805	-4.380278	-1.740792
C	-0.483434	-4.203287	0.636609
H	0.248474	-3.540841	1.09315
H	-1.358528	-4.248555	1.284043
H	-0.053133	-5.193315	0.544568
C	-2.912709	-0.861648	-0.063765
H	-3.608466	-1.63433	-0.373498
C	-2.844935	0.198425	-1.139109
H	-2.374948	-0.196062	-2.037978
H	-2.305086	1.074219	-0.791985
O	-3.348585	-0.248083	1.162137
O	-4.204371	0.564898	-1.430392
C	-4.376073	1.623379	-2.250068
C	-4.584592	-0.436882	1.689329
C	-5.539742	-1.374802	1.006782
H	-6.466436	-1.380991	1.569161
H	-5.733772	-1.054144	-0.014944
H	-5.133804	-2.385663	0.974814
C	-5.831307	1.919533	-2.455683
H	-6.305095	2.119568	-1.495477
H	-5.942873	2.776401	-3.110905
H	-6.324891	1.051559	-2.891189
O	-4.845074	0.168155	2.701724
O	-3.450244	2.229895	-2.737368

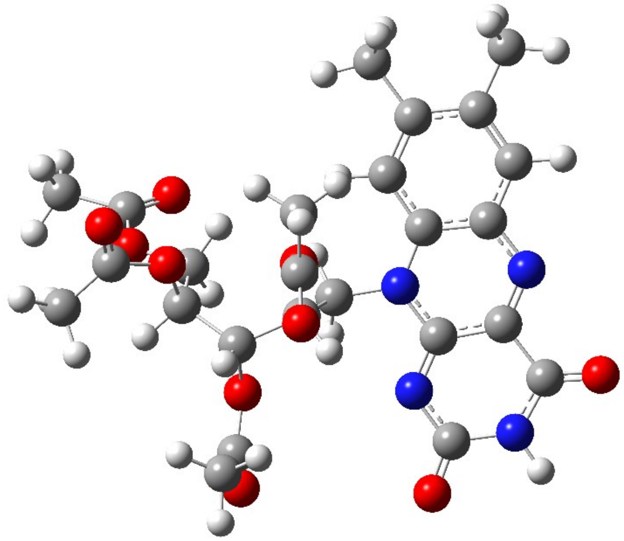

RFT_singlet_B3LYP-D3BJ_6-311_plus_2d2p_H2O_maxdisk8GB_2

Zero-point correction= 0.522244 (Hartree/Particle)
Thermal correction to Energy= 0.561251
Thermal correction to Enthalpy= 0.562195
Thermal correction to Gibbs Free Energy= 0.445085
Sum of electronic and zero-point Energies= -1941.137255
Sum of electronic and thermal Energies= -1941.098248
Sum of electronic and thermal Enthalpies= -1941.097304
Sum of electronic and thermal Free Energies= -1941.214414

C	5.050139	-1.570165	-0.083691
C	4.032889	-0.491479	-0.189727
C	2.671476	-0.897164	-0.445565
C	3.17337	-3.153158	-0.469553
C	2.103945	1.432477	-0.372435
C	3.470176	1.723922	-0.121099
C	3.868505	3.062615	0.064559
H	4.917371	3.24356	0.250917
C	2.968382	4.098826	0.019316
C	1.597122	3.797026	-0.219725
C	1.188893	2.488978	-0.410479
H	5.159928	-3.605323	-0.167837
H	0.140744	2.301799	-0.559521
N	1.747533	0.107401	-0.558906
N	4.405958	0.744602	-0.042168
N	2.262415	-2.141319	-0.569941
N	4.520142	-2.825696	-0.231544
C	0.580018	4.898686	-0.255418
H	0.567044	5.445594	0.688743
H	0.819451	5.623543	-1.035132
H	-0.417403	4.509476	-0.441527
C	3.414171	5.518359	0.225612
H	3.17435	6.137105	-0.640386

H	2.914351	5.967747	1.085111
H	4.487371	5.56659	0.392263
O	2.875184	-4.335926	-0.574844
O	6.238063	-1.380353	0.116461
C	0.363987	-0.292971	-0.853439
C	-0.338625	-0.724872	0.428312
O	-0.64148	0.47492	1.156443
C	-0.741642	0.380154	2.504743
O	-0.577135	-0.657293	3.100394
H	0.355886	-1.317061	1.015169
H	0.410938	-1.126062	-1.541346
H	-0.138483	0.530406	-1.341219
C	-1.092494	1.705083	3.103506
H	-0.462543	2.488331	2.686698
H	-2.126955	1.933103	2.845357
H	-0.987589	1.662932	4.182148
C	-1.61127	-1.575835	0.225742
H	-1.782247	-2.135836	1.139227
O	-1.420371	-2.462776	-0.885879
C	-0.888167	-3.711753	-0.754442
O	-0.770366	-4.360059	-1.763647
C	-0.501842	-4.19421	0.612737
H	0.248625	-3.542367	1.054129
H	-1.367078	-4.225622	1.273941
H	-0.088217	-5.190766	0.514654
C	-2.927742	-0.837937	-0.060851
H	-3.630698	-1.602869	-0.373417
C	-2.852771	0.227975	-1.1298
H	-2.387549	-0.165026	-2.031795
H	-2.304616	1.096616	-0.777829
O	-3.355857	-0.227476	1.169487
O	-4.209538	0.60786	-1.416756
C	-4.373586	1.67324	-2.228872
C	-4.591457	-0.410212	1.699333
C	-5.555493	-1.337345	1.014437
H	-6.480593	-1.339848	1.579462
H	-5.7501	-1.009624	-0.004951
H	-5.157071	-2.350978	0.975677
C	-5.826629	1.982941	-2.430255
H	-6.296973	2.181702	-1.468086
H	-5.932026	2.844408	-3.080449
H	-6.328446	1.121685	-2.869665
O	-4.844719	0.191163	2.715801
O	-3.443525	2.275289	-2.713723

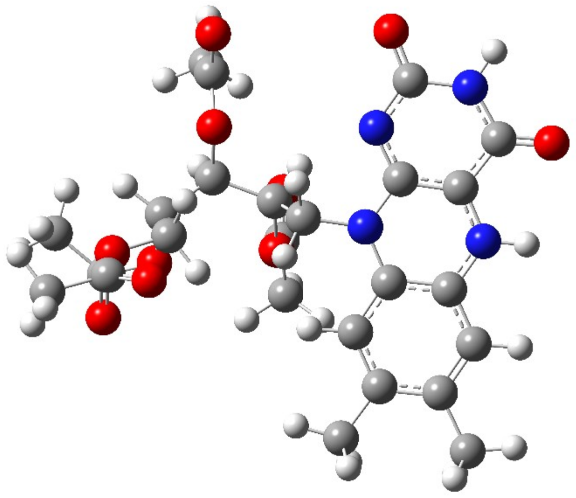

H-RFT_rad_B3LYP-D3BJ_6-311_plus_2d2p_H2O_maxdisk8GB_2

Zero-point correction= 0.533430 (Hartree/Particle)
Thermal correction to Energy= 0.572958
Thermal correction to Enthalpy= 0.573902
Thermal correction to Gibbs Free Energy= 0.454293
Sum of electronic and zero-point Energies= -1941.730553
Sum of electronic and thermal Energies= -1941.691025
Sum of electronic and thermal Enthalpies= -1941.690081
Sum of electronic and thermal Free Energies= -1941.809690

C	5.022573	-1.552497	-0.088382
C	4.012734	-0.537278	-0.207978
C	2.676934	-0.907945	-0.447962
C	3.185566	-3.167862	-0.467883
C	2.087319	1.443686	-0.365533
C	3.438412	1.778793	-0.13543
C	3.815098	3.110987	0.046847
H	4.859689	3.331927	0.218409
C	2.884069	4.132577	0.017892
C	1.525823	3.806578	-0.19602
C	1.155422	2.481183	-0.381721
H	5.18079	-3.593053	-0.157699
H	0.110652	2.264386	-0.513367
N	1.737778	0.097265	-0.563308
N	4.366384	0.767529	-0.084109
N	2.270971	-2.163555	-0.567402
N	4.526405	-2.82768	-0.229182
C	0.475208	4.88052	-0.212805
H	0.454895	5.425714	0.73246
H	0.673868	5.614232	-0.995937
H	-0.512611	4.459545	-0.383629
C	3.311623	5.558517	0.220585
H	3.046523	6.175178	-0.639764
H	2.818003	5.998046	1.088932

H	4.386503	5.626883	0.369854
O	2.89626	-4.359587	-0.573525
O	6.215198	-1.31016	0.119713
C	0.361117	-0.30846	-0.851028
C	-0.347806	-0.740128	0.428695
O	-0.654335	0.45786	1.160816
C	-0.751959	0.363275	2.507312
O	-0.584548	-0.672986	3.105479
H	0.347246	-1.331879	1.015195
H	0.405063	-1.145964	-1.534207
H	-0.147637	0.508134	-1.345565
C	-1.104876	1.687954	3.106706
H	-0.481434	2.473443	2.684365
H	-2.142111	1.910864	2.855193
H	-0.992911	1.648281	4.18478
C	-1.621759	-1.588631	0.228905
H	-1.796933	-2.143618	1.144732
O	-1.433203	-2.483649	-0.877637
C	-0.896295	-3.728775	-0.73976
O	-0.780208	-4.384493	-1.744946
C	-0.500517	-4.200121	0.628611
H	0.263637	-3.550836	1.050129
H	-1.357051	-4.213798	1.3015
H	-0.099309	-5.20243	0.537667
C	-2.936121	-0.849765	-0.064974
H	-3.638961	-1.613914	-0.37968
C	-2.85507	0.214698	-1.134795
H	-2.387192	-0.180426	-2.034402
H	-2.306202	1.082585	-0.782314
O	-3.370042	-0.236739	1.162511
O	-4.210211	0.597123	-1.427539
C	-4.369114	1.662643	-2.24008
C	-4.608073	-0.417488	1.686095
C	-5.569103	-1.346283	0.999022
H	-6.496569	-1.347874	1.56017
H	-5.759532	-1.020393	-0.021756
H	-5.170346	-2.35988	0.963623
C	-5.820815	1.97605	-2.445932
H	-6.291177	2.184401	-1.485742
H	-5.922246	2.83303	-3.102663
H	-6.325748	1.113396	-2.87886
O	-4.866981	0.186786	2.699649
O	-3.436237	2.263101	-2.721698
H	5.342429	0.967699	0.091666

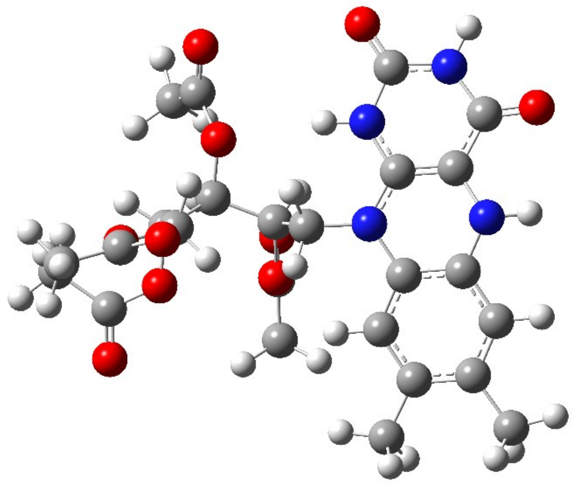

H2-RFT_B3LYP_6-311_plus_2d2p_H2O_maxdisk8GB
Zero-point correction= 0.545606 (Hartree/Particle)
Thermal correction to Energy= 0.585430
Thermal correction to Enthalpy= 0.586374
Thermal correction to Gibbs Free Energy= 0.468058
Sum of electronic and zero-point Energies= -1942.321474
Sum of electronic and thermal Energies= -1942.281650
Sum of electronic and thermal Enthalpies= -1942.280706
Sum of electronic and thermal Free Energies= -1942.399022

C	4.539854	2.497564	0.351126
C	3.838914	1.276309	0.537
C	2.473153	1.246484	0.521441
C	2.360211	3.658534	0.204444
C	2.520324	-1.14956	0.421054
C	3.920788	-1.110445	0.471196
C	4.637818	-2.277921	0.248187
H	5.718615	-2.233781	0.286445
C	4.008094	-3.497967	-0.002867
C	2.610649	-3.539184	-0.043176
C	1.891318	-2.358139	0.160248
H	4.197309	4.513401	0.064292
H	0.818065	-2.402328	0.094397
N	1.787063	0.063705	0.650278
N	4.565973	0.094865	0.798712
N	1.76959	2.421509	0.373074
N	3.728518	3.629086	0.193559
C	1.875362	-4.824695	-0.315037
H	2.135854	-5.235377	-1.292777
H	2.120505	-5.59039	0.423552
H	0.798341	-4.670854	-0.29194
C	4.828289	-4.739449	-0.22726
H	4.596207	-5.507823	0.512779
H	4.62723	-5.176126	-1.207425

H	5.892694	-4.522795	-0.166287
O	1.700422	4.679739	0.070898
O	5.771299	2.605183	0.331926
C	0.33443	0.061199	0.745687
C	-0.382123	0.22483	-0.608838
O	-0.670226	-1.088834	-1.110523
C	-0.806949	-1.232207	-2.452094
O	-0.689726	-0.309696	-3.222472
H	0.287008	0.721635	-1.308095
H	0.038881	0.85575	1.423256
H	0.03281	-0.866135	1.218716
C	-1.114439	-2.652715	-2.805685
H	-0.318493	-3.303406	-2.446179
H	-2.037519	-2.952416	-2.311528
H	-1.218152	-2.750232	-3.880617
C	-1.680569	1.053169	-0.57941
H	-1.911135	1.309262	-1.606758
O	-1.390924	2.23375	0.190998
C	-1.782778	3.492661	-0.207261
O	-1.73801	4.357724	0.6246
C	-2.17483	3.703191	-1.639915
H	-1.40661	3.322862	-2.311769
H	-3.109952	3.198333	-1.87725
H	-2.303183	4.767939	-1.798125
C	-2.94683	0.394867	-0.002415
H	-3.656091	1.196967	0.177758
C	-2.745668	-0.373635	1.283977
H	-2.23295	0.240103	2.02168
H	-2.184331	-1.287388	1.110235
O	-3.446086	-0.501075	-1.011329
O	-4.056885	-0.706877	1.768457
C	-4.10637	-1.485812	2.870332
C	-4.740891	-0.517665	-1.421488
C	-5.711668	0.474041	-0.844764
H	-6.688804	0.272681	-1.268849
H	-5.757067	0.389058	0.23872
H	-5.420648	1.493939	-1.09499
C	-5.522887	-1.774864	3.26662
H	-6.040898	-2.269353	2.445878
H	-5.536108	-2.405889	4.148427
H	-6.045164	-0.840686	3.469627
O	-5.039445	-1.344416	-2.249453
O	-3.113206	-1.878476	3.437138
H	0.76048	2.437353	0.389297
H	5.528182	0.163822	0.49964

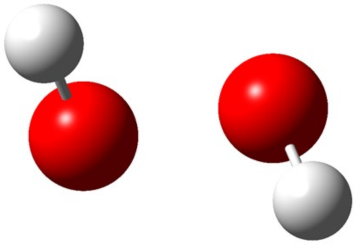

HOOH_B3LYP-D3BJ_6-311_plus_2d2p_H2O
Zero-point correction= 0.026491 (Hartree/Particle)
Thermal correction to Energy= 0.029706
Thermal correction to Enthalpy= 0.030650
Thermal correction to Gibbs Free Energy= 0.004201
Sum of electronic and zero-point Energies= -151.591121
Sum of electronic and thermal Energies= -151.587905
Sum of electronic and thermal Enthalpies= -151.586961
Sum of electronic and thermal Free Energies= -151.613410

O	-0.716939	-0.105009	-0.066592
O	0.716938	0.104998	-0.066639
H	-1.013953	0.594004	0.532888
H	1.013954	-0.593915	0.532957

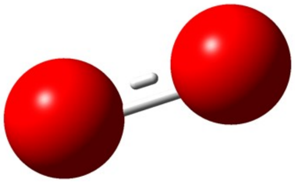

O2_triplet_B3LYP-D3BJ_6-311_plus_2d2p_H2O
Zero-point correction= 0.003710 (Hartree/Particle)
Thermal correction to Energy= 0.006074
Thermal correction to Enthalpy= 0.007018
Thermal correction to Gibbs Free Energy= -0.016259
Sum of electronic and zero-point Energies= -150.371512
Sum of electronic and thermal Energies= -150.369149
Sum of electronic and thermal Enthalpies= -150.368205
Sum of electronic and thermal Free Energies= -150.391481

O	0	0	0.604018
O	0	0	-0.604018

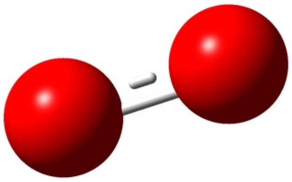

O2_singlet_B3LYP-D3BJ_6-311_plus_2d2p_H2O

Zero-point correction= 0.003685 (Hartree/Particle)
Thermal correction to Energy= 0.006048
Thermal correction to Enthalpy= 0.006993
Thermal correction to Gibbs Free Energy= -0.015246
Sum of electronic and zero-point Energies= -150.310839
Sum of electronic and thermal Energies= -150.308476
Sum of electronic and thermal Enthalpies= -150.307532
Sum of electronic and thermal Free Energies= -150.329770

O	0	0	0.603783
O	0	0	-0.603783

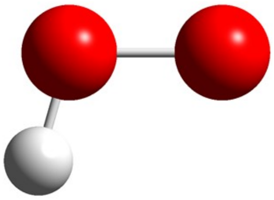

HOO_rad_B3LYP-D3BJ_6-311_plus_2d2p_H2O
Zero-point correction= 0.014213 (Hartree/Particle)
Thermal correction to Energy= 0.017070
Thermal correction to Enthalpy= 0.018014
Thermal correction to Gibbs Free Energy= -0.007961
Sum of electronic and zero-point Energies= -150.956635
Sum of electronic and thermal Energies= -150.953778
Sum of electronic and thermal Enthalpies= -150.952834
Sum of electronic and thermal Free Energies= -150.978808

O	0.05527	-0.60853	0
O	0.05527	0.717945	0
H	-0.884312	-0.875317	0

<u>B3LYP/6-311+G (2d,2p) //CPCM(water)</u>

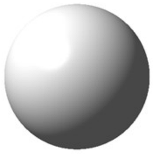

H_b3lyp_6311gplus2d2p_H2O
Zero-point correction= 0.000000 (Hartree/Particle)
Thermal correction to Energy= 0.001416
Thermal correction to Enthalpy= 0.002360
Thermal correction to Gibbs Free Energy= -0.010654
Sum of electronic and zero-point Energies= -0.502177
Sum of electronic and thermal Energies= -0.500761

Sum of electronic and thermal Enthalpies= -0.499817
Sum of electronic and thermal Free Energies= -0.512831

H 0 0 0

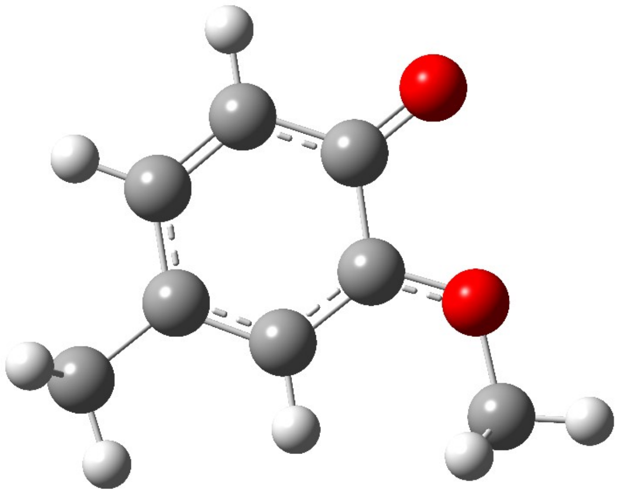

2-methoxy4-methyl-phenol_rad_b3lyp_6311gplus2d2p_H2O_2
Zero-point correction= 0.151694 (Hartree/Particle)
Thermal correction to Energy= 0.161325
Thermal correction to Enthalpy= 0.162269
Thermal correction to Gibbs Free Energy= 0.116003
Sum of electronic and zero-point Energies= -460.680697
Sum of electronic and thermal Energies= -460.671065
Sum of electronic and thermal Enthalpies= -460.670121
Sum of electronic and thermal Free Energies= -460.716387

C	-1.632535	-0.473051	0.000008
C	-0.322809	-0.95757	0.000032
C	0.76091	-0.089846	0.000033
C	0.556208	1.365544	-0.000009
C	-0.81615	1.811905	-0.000015
C	-1.856976	0.930547	-0.000015
H	-0.163895	-2.024982	0.000056
H	-2.875594	1.295493	-0.000029
O	1.522359	2.162859	-0.000009
O	2.043379	-0.467018	0.000067
C	2.35831	-1.867243	-0.000058
H	3.441283	-1.921269	-0.000166
H	1.963116	-2.350231	0.89277
H	1.962941	-2.350112	-0.892873
H	-0.980784	2.880509	-0.000027
C	-2.80135	-1.412751	-0.000004
H	-2.48369	-2.452812	0.000022
H	-3.431485	-1.244251	0.876032

H	-3.431434	-1.244282	-0.876084

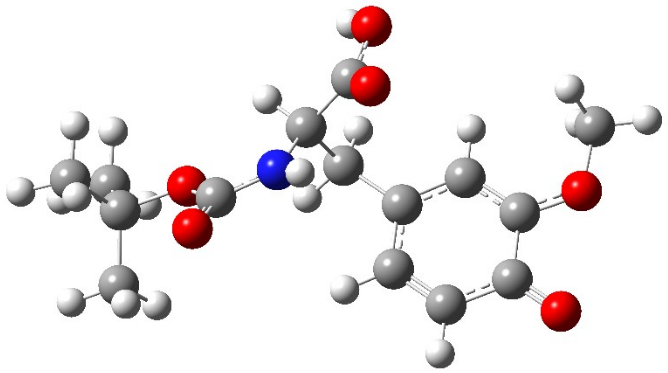

2-methoxy-4ethyl-COOH-NHBoc-phenol_rad_b3lyp_6311gplus2d2p_H2O_2
Zero-point correction= 0.338743 (Hartree/Particle)
Thermal correction to Energy= 0.361862
Thermal correction to Enthalpy= 0.362806
Thermal correction to Gibbs Free Energy= 0.283347
Sum of electronic and zero-point Energies= -1089.774401
Sum of electronic and thermal Energies= -1089.751282
Sum of electronic and thermal Enthalpies= -1089.750338
Sum of electronic and thermal Free Energies= -1089.829797

C	1.353339	-0.323161	-0.65031
C	2.636654	0.224137	-0.733032
C	3.745256	-0.486386	-0.29447
C	3.594247	-1.838282	0.26636
C	2.248846	-2.357626	0.317506
C	1.181229	-1.630347	-0.122792
H	2.758969	1.211038	-1.152387
H	0.186952	-2.051548	-0.07953
O	4.582716	-2.49246	0.666143
O	5.00349	-0.044597	-0.336736
C	5.26862	1.264168	-0.865289
H	6.342251	1.394852	-0.790037
H	4.959454	1.326166	-1.907845
H	4.759463	2.02566	-0.276169
H	2.125907	-3.354458	0.717264
C	0.162813	0.471378	-1.114154
H	0.466558	1.184187	-1.880593
H	-0.574727	-0.188405	-1.568351
C	-0.598417	1.247168	0.01219
H	-1.38853	1.813523	-0.48003
C	0.323468	2.233618	0.727336
O	0.732492	3.298927	0.018116
H	0.309963	3.348212	-0.849491
O	0.709552	2.083949	1.859085
N	-1.189462	0.384592	1.010856
H	-0.718908	0.323024	1.899618

C	-2.429956	-0.177751	0.954711
O	-2.907514	-0.811675	1.88252
O	-3.01228	0.051526	-0.23346
C	-4.377867	-0.437366	-0.551928
C	-4.40489	-1.963612	-0.520384
H	-5.369948	-2.310704	-0.889661
H	-3.628654	-2.3711	-1.168026
H	-4.261514	-2.343518	0.486752
C	-5.391389	0.190935	0.40161
H	-6.398148	-0.064276	0.070725
H	-5.258764	-0.168776	1.417729
H	-5.298187	1.276947	0.393728
C	-4.584002	0.07708	-1.974208
H	-4.520202	1.164263	-2.00441
H	-3.832706	-0.335857	-2.646454
H	-5.568346	-0.220459	-2.333039

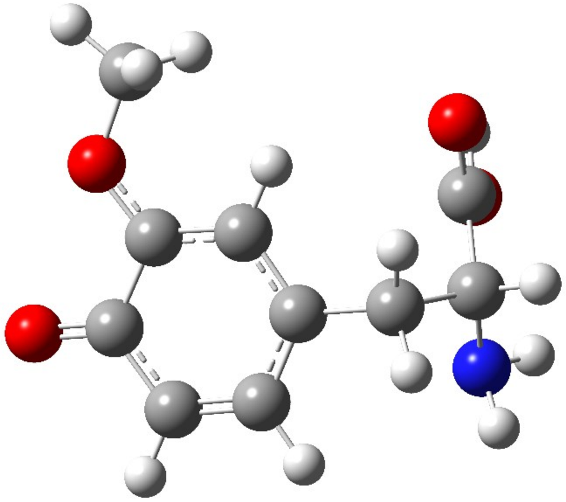

2-methoxy-4ethyl-COOH-NH2-phenol_rad_b3lyp_6311gplus2d2p_H2O
Zero-point correction= 0.213190 (Hartree/Particle)
Thermal correction to Energy= 0.227851
Thermal correction to Enthalpy= 0.228795
Thermal correction to Gibbs Free Energy= 0.169116
Sum of electronic and zero-point Energies= -743.952612
Sum of electronic and thermal Energies= -743.937951
Sum of electronic and thermal Enthalpies= -743.937007
Sum of electronic and thermal Free Energies= -743.996686

C	0.126842	-0.757243	0.784165
C	-0.653558	0.393151	0.633866
C	-1.923057	0.327269	0.077496
C	-2.483129	-0.959837	-0.362308
C	-1.640953	-2.115627	-0.171973
C	-0.395552	-2.013002	0.375864
H	-0.253594	1.339581	0.962664

H	0.209916	-2.898393	0.509118
O	-3.626854	-1.037035	-0.864937
O	-2.735339	1.372565	-0.102786
C	-2.2893	2.678932	0.291548
H	-3.104243	3.35036	0.04516
H	-2.093617	2.710491	1.362612
H	-1.39468	2.962179	-0.261479
H	-2.044435	-3.069272	-0.482819
C	1.505555	-0.66357	1.383771
H	1.541834	0.146314	2.109912
H	1.734838	-1.587101	1.916092
C	2.656869	-0.432524	0.36884
H	3.567451	-0.334507	0.974256
C	2.523736	0.933214	-0.292139
O	2.612527	0.904657	-1.63096
H	2.560666	1.817772	-1.953509
O	2.388397	1.96098	0.332343
N	2.735495	-1.523084	-0.599822
H	3.474528	-1.349173	-1.269964
H	2.978301	-2.38058	-0.117343

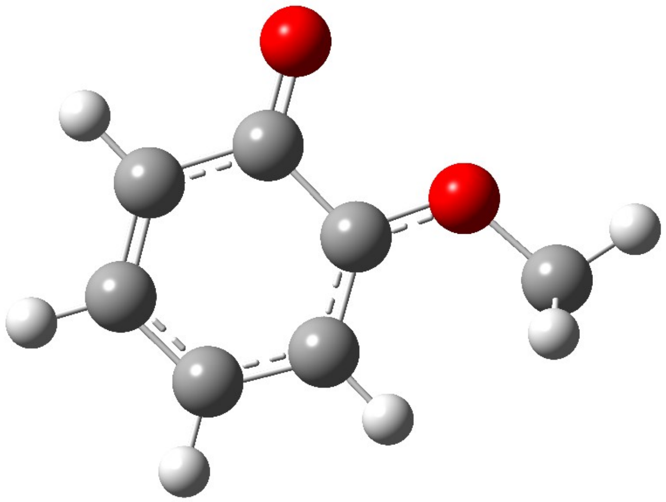

2-methoxyphenol_rad_b3lyp_6311gplus2d2p_H2O
Zero-point correction= 0.124431 (Hartree/Particle)
Thermal correction to Energy= 0.132223
Thermal correction to Enthalpy= 0.133168
Thermal correction to Gibbs Free Energy= 0.091430
Sum of electronic and zero-point Energies= -421.375763
Sum of electronic and thermal Energies= -421.367970
Sum of electronic and thermal Enthalpies= -421.367026
Sum of electronic and thermal Free Energies= -421.408763

C	-1.232309	-1.68152	-0.00002
C	0.114994	-1.338221	-0.000002
C	0.488978	0.001079	0.000003

C	-0.533549	1.061614	-0.000012
C	-1.911985	0.629846	-0.000031
C	-2.245027	-0.695026	-0.000034
H	0.859888	-2.11839	0.000007
H	-3.282209	-0.999222	-0.000048
O	-0.213226	2.271043	-0.000006
O	1.746051	0.447199	0.000018
C	2.823599	-0.502508	0.000062
H	3.731759	0.089651	0.000106
H	2.785622	-1.124786	0.893182
H	2.785703	-1.124774	-0.89307
H	-2.663945	1.406514	-0.000042
H	-1.507625	-2.72651	-0.000024

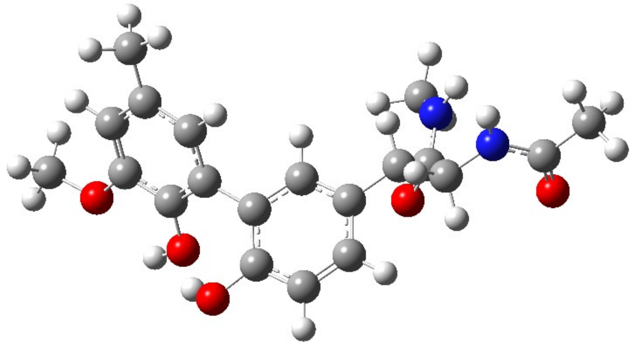

phenol-peptide_plus_2-methoxy4-methyl-phenol_b3lyp_6311gplus2d2p_H2O
Zero-point correction= 0.415548 (Hartree/Particle)
Thermal correction to Energy= 0.443625
Thermal correction to Enthalpy= 0.444570
Thermal correction to Gibbs Free Energy= 0.353253
Sum of electronic and zero-point Energies= -1262.257325
Sum of electronic and thermal Energies= -1262.229248
Sum of electronic and thermal Enthalpies= -1262.228304
Sum of electronic and thermal Free Energies= -1262.319620

C	1.262917	-1.175678	0.871518
C	0.06045	-0.483185	0.791635
C	-1.151089	-1.090294	0.424235
C	-1.117117	-2.459486	0.100794
C	0.079637	-3.170752	0.195083
C	1.251553	-2.543044	0.582684
H	0.050463	0.573927	1.021382
H	2.164179	-3.121035	0.652145
O	-2.231397	-3.175628	-0.248792
H	0.067684	-4.226366	-0.038255
C	2.538511	-0.477414	1.27935
C	3.554966	-0.233802	0.13702
H	3.053733	-1.080812	2.029357
H	2.300347	0.478611	1.748059
H	3.780709	-1.177783	-0.355529

C	5.973321	0.253255	0.065295
C	7.18063	0.727092	0.840919
H	7.665633	1.525673	0.281568
C	2.972847	0.66763	-0.966397
N	3.149979	1.991264	-0.817757
H	3.741528	2.313117	-0.070632
O	2.345597	0.176485	-1.904776
N	4.783122	0.294256	0.718545
H	4.778807	0.558538	1.690112
O	6.059057	-0.142707	-1.095982
H	6.937604	1.086315	1.838179
H	7.888006	-0.097483	0.922526
C	2.647528	2.959271	-1.780794
H	1.567655	2.870229	-1.886715
H	2.886964	3.95698	-1.425466
H	3.103114	2.812035	-2.760172
C	-2.399271	-0.279481	0.473527
C	-2.634171	0.562261	1.579059
C	-3.353939	-0.283327	-0.537086
C	-3.761727	1.362874	1.681002
H	-1.912501	0.564828	2.383806
C	-4.504026	0.518295	-0.453823
C	-4.705927	1.336802	0.64404
H	-2.805958	-2.615229	-0.798606
H	-5.590137	1.953092	0.709746
O	-3.18091	-1.075242	-1.652279
H	-3.956689	-0.965436	-2.221122
O	-5.337712	0.39741	-1.529962
C	-6.539862	1.172518	-1.552174
H	-6.313123	2.238525	-1.531934
H	-7.036597	0.92116	-2.48329
H	-7.182563	0.913925	-0.710572
C	-3.988915	2.244325	2.884479
H	-4.067811	3.293992	2.595914
H	-4.915643	1.98208	3.397992
H	-3.172084	2.152767	3.598133

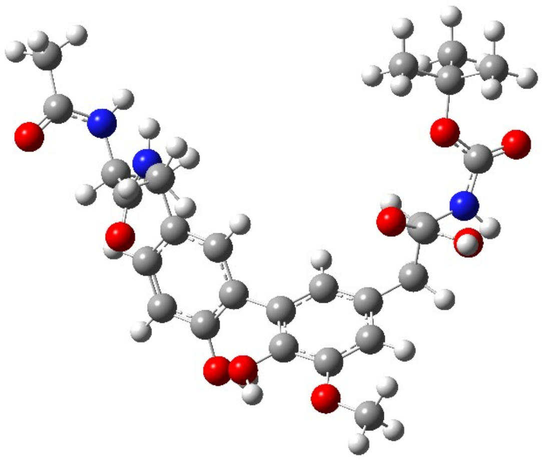

phenol-peptide_plus_2-methoxy-4ethyl-COOH-NHBoc-phenol_b3lyp_6311gplus2d2p_H2O
Zero-point correction= 0.601853 (Hartree/Particle)
Thermal correction to Energy= 0.644120
Thermal correction to Enthalpy= 0.645064
Thermal correction to Gibbs Free Energy= 0.517758
Sum of electronic and zero-point Energies= -1891.355380
Sum of electronic and thermal Energies= -1891.313113
Sum of electronic and thermal Enthalpies= -1891.312169
Sum of electronic and thermal Free Energies= -1891.439475

C	-3.148796	-0.202538	-1.496734
C	-1.950935	-0.686546	-0.978628
C	-1.507262	-1.994345	-1.204002
C	-2.305983	-2.834854	-1.991627
C	-3.504341	-2.365742	-2.527469
C	-3.917516	-1.065782	-2.282296
H	-1.334333	-0.036506	-0.371379
H	-4.848612	-0.720274	-2.712887
O	-1.949834	-4.118066	-2.303942
H	-4.095142	-3.02861	-3.14425
C	-3.594413	1.216594	-1.235714
C	-4.786877	1.368676	-0.259833
H	-3.903898	1.676672	-2.176431
H	-2.754143	1.800672	-0.857043
H	-5.62821	0.77966	-0.620223
C	-6.399256	3.17984	0.2047
C	-6.704693	4.653025	0.062146
H	-6.954837	5.055758	1.042567
C	-4.465255	0.800482	1.133961
N	-3.919981	1.651534	2.019205
H	-3.860593	2.624362	1.770955
O	-4.670115	-0.387406	1.383706
N	-5.178907	2.772485	-0.230782
H	-4.583584	3.444962	-0.68568
O	-7.206658	2.391491	0.693894
H	-5.881039	5.224881	-0.358949

H	-7.58006	4.767025	-0.57625
C	-3.562746	1.250947	3.371461
H	-2.859993	0.41988	3.351482
H	-3.097644	2.095604	3.870777
H	-4.443001	0.944623	3.9371
C	-0.197507	-2.445141	-0.654417
C	0.991261	-1.82043	-1.059129
C	-0.116121	-3.473902	0.283798
C	2.229341	-2.199116	-0.555729
H	0.929198	-1.030493	-1.795658
C	1.131044	-3.873501	0.794652
C	2.292263	-3.241265	0.37721
H	-1.122628	-4.344342	-1.862429
H	3.249248	-3.553519	0.767336
O	-1.252488	-4.093944	0.721969
H	-0.993176	-4.77534	1.357555
O	1.056107	-4.892074	1.700661
C	2.267064	-5.368475	2.29547
H	2.764426	-4.573253	2.850876
H	1.970644	-6.160081	2.975415
H	2.939764	-5.768473	1.536682
C	3.491728	-1.508467	-1.017439
H	4.358876	-2.133133	-0.799971
H	3.461615	-1.365845	-2.098042
C	3.725543	-0.104129	-0.405763
H	2.866246	0.524679	-0.62071
C	3.810156	-0.18241	1.119024
O	2.879151	0.033404	1.857862
O	5.025671	-0.552791	1.556363
H	4.996379	-0.608141	2.523727
N	4.90663	0.474814	-1.019982
H	5.625127	-0.148022	-1.350981
C	5.298884	1.779279	-0.921726
O	6.359201	2.184088	-1.371453
O	4.368937	2.514769	-0.292844
C	4.512648	3.981653	-0.10924
C	3.222036	4.347146	0.619265
H	2.352275	4.093158	0.014379
H	3.152662	3.819494	1.569792
H	3.203322	5.417999	0.817388
C	5.728893	4.279902	0.764188
H	6.655118	4.027499	0.256599
H	5.742369	5.342711	1.005853
H	5.67194	3.721344	1.698437
C	4.578557	4.671875	-1.469455
H	4.54368	5.751492	-1.323409
H	5.49369	4.423415	-1.998798
H	3.724015	4.385866	-2.082691

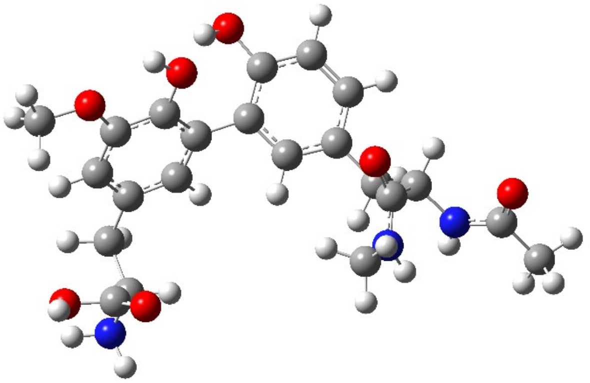

phenol-peptide_plus_2-methoxy-4ethyl-COOH-NH2-phenol_b3lyp_6311gplus2d2p_H2O
Zero-point correction= 0.476894 (Hartree/Particle)
Thermal correction to Energy= 0.510193
Thermal correction to Enthalpy= 0.511137
Thermal correction to Gibbs Free Energy= 0.406247
Sum of electronic and zero-point Energies= -1545.533583
Sum of electronic and thermal Energies= -1545.500284
Sum of electronic and thermal Enthalpies= -1545.499340
Sum of electronic and thermal Free Energies= -1545.604230

C	2.125352	1.096014	-1.200983
C	0.816823	0.778813	-0.856258
C	-0.196528	1.742141	-0.728032
C	0.157649	3.087752	-0.937682
C	1.463499	3.417864	-1.301758
C	2.432349	2.438259	-1.440257
H	0.560447	-0.25662	-0.675684
H	3.436122	2.722832	-1.728675
O	-0.740299	4.11998	-0.868867
H	1.698555	4.458397	-1.477521
C	3.178148	0.021992	-1.336983
C	4.226676	-0.028641	-0.198752
H	3.734267	0.180283	-2.263141
H	2.698762	-0.954837	-1.416345
H	4.693053	0.948896	-0.090984
C	6.488643	-0.996825	0.001809
C	7.486195	-1.979788	-0.566102
H	7.857298	-2.608845	0.241579
C	3.57773	-0.319261	1.166274
N	3.480546	-1.610705	1.523727
H	3.930118	-2.300448	0.946215
O	3.142406	0.602532	1.856306
N	5.253475	-0.996934	-0.563779
H	5.094823	-1.583214	-1.366716
O	6.774227	-0.241005	0.929024
H	7.071234	-2.610904	-1.34856
H	8.332171	-1.423912	-0.969001
C	2.886058	-2.031622	2.783096

H	1.85589	-1.687013	2.855851
H	2.899911	-3.116552	2.826012
H	3.44282	-1.635227	3.632381
C	-1.586971	1.281557	-0.458574
C	-2.102337	0.184858	-1.171311
C	-2.417133	1.869798	0.492591
C	-3.381452	-0.310636	-0.953325
H	-1.48252	-0.269053	-1.932084
C	-3.714286	1.389655	0.72373
C	-4.19405	0.304236	0.00703
H	-1.364704	3.946696	-0.143646
H	-5.19398	-0.063817	0.178828
O	-1.969416	2.943044	1.22958
H	-2.689407	3.231142	1.809535
O	-4.395873	2.081716	1.683901
C	-5.728128	1.67114	2.007058
H	-5.736886	0.648035	2.382867
H	-6.065815	2.349505	2.783309
H	-6.380077	1.753566	1.137464
C	-3.888243	-1.499453	-1.7395
H	-4.978649	-1.514562	-1.733078
H	-3.574485	-1.422324	-2.780484
C	-3.37101	-2.862224	-1.233526
H	-2.283525	-2.866285	-1.273385
C	-3.725631	-3.090233	0.23452
O	-2.922134	-3.221726	1.13001
O	-5.056196	-3.157126	0.439145
H	-5.212532	-3.326067	1.380486
N	-3.85892	-3.930773	-2.109606
H	-4.866504	-4.018294	-2.038994
H	-3.463933	-4.824173	-1.83979

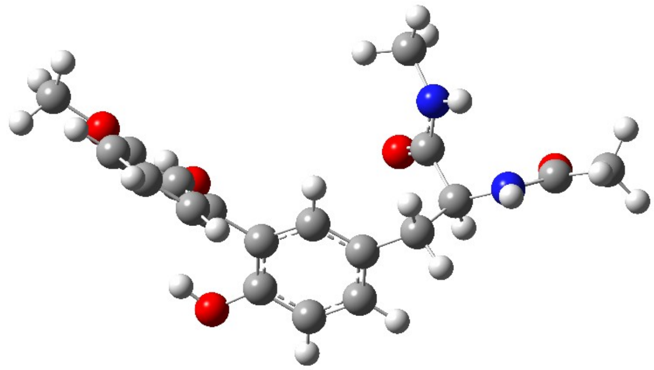

phenol-peptide_plus_2-methoxyphenol_b3lyp_6311gplus2d2p_H2O
Zero-point correction= 0.387726 (Hartree/Particle)
Thermal correction to Energy= 0.414369
Thermal correction to Enthalpy= 0.415313
Thermal correction to Gibbs Free Energy= 0.326959
Sum of electronic and zero-point Energies= -1222.954287

Sum of electronic and thermal Energies= -1222.927644
Sum of electronic and thermal Enthalpies= -1222.926700
Sum of electronic and thermal Free Energies= -1223.015055

C	1.105005	-1.495289	0.465116
C	-0.083167	-0.834066	0.764108
C	-1.32111	-1.244467	0.258015
C	-1.351538	-2.374869	-0.570686
C	-0.173967	-3.055644	-0.875252
C	1.037788	-2.619084	-0.362666
H	-0.058509	0.036943	1.4063
H	1.93991	-3.165329	-0.606801
O	-2.51152	-2.884362	-1.086314
H	-0.226167	-3.932787	-1.505171
C	2.422904	-1.019752	1.028177
C	3.341823	-0.277554	0.026917
H	2.990972	-1.876873	1.395093
H	2.240062	-0.368494	1.884259
H	3.536954	-0.918938	-0.830628
C	5.738306	0.316405	0.008043
C	6.997156	0.470939	0.829848
H	7.435655	1.445341	0.620688
C	2.664539	0.976242	-0.555182
N	2.81328	2.116733	0.139616
H	3.442962	2.118888	0.923913
O	1.992185	0.899801	-1.583357
N	4.605814	0.012313	0.693306
H	4.678561	-0.166494	1.681465
O	5.73498	0.462024	-1.213222
H	6.825979	0.377793	1.899795
H	7.712731	-0.288984	0.517282
C	2.226896	3.377072	-0.290169
H	1.148605	3.278905	-0.402338
H	2.437193	4.130819	0.462692
H	2.64448	3.701267	-1.243722
C	-2.563757	-0.515375	0.638933
C	-2.970419	-0.455984	1.982281
C	-3.335998	0.152681	-0.311827
C	-4.107814	0.23957	2.35548
H	-2.380584	-0.97169	2.726781
C	-4.494841	0.856876	0.066752
C	-4.880977	0.900255	1.39759
H	-3.255549	-2.328168	-0.82638
H	-5.767344	1.438214	1.695961
O	-2.958981	0.144588	-1.625643
H	-3.612605	0.653225	-2.125253
O	-5.137588	1.460665	-0.976453
C	-6.314581	2.225294	-0.69994

H	-6.089379	3.052066	-0.026083
H	-6.647224	2.612983	-1.657008
H	-7.0926	1.595868	-0.267799
H	-4.407872	0.270894	3.393195

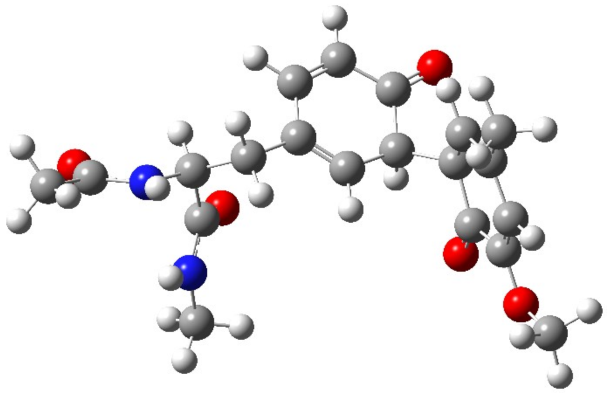

ketone-peptide_plus_2-methoxy4-methyl-phenone_b3lyp_6311gplus2d2p_H2O
Zero-point correction= 0.413558 (Hartree/Particle)
Thermal correction to Energy= 0.441792
Thermal correction to Enthalpy= 0.442736
Thermal correction to Gibbs Free Energy= 0.350182
Sum of electronic and zero-point Energies= -1262.196089
Sum of electronic and thermal Energies= -1262.167855
Sum of electronic and thermal Enthalpies= -1262.166911
Sum of electronic and thermal Free Energies= -1262.259465

C	-0.834984	1.112928	0.213948
C	0.311814	0.511766	-0.136372
C	1.3654	1.180896	-0.962524
C	1.158384	2.673924	-1.211042
C	-0.132185	3.234608	-0.840477
C	-1.045086	2.502841	-0.168552
H	0.489018	-0.520355	0.140033
H	-1.975662	2.973555	0.124826
O	2.04289	3.353394	-1.726903
H	-0.305655	4.275112	-1.077015
C	-1.894278	0.4154	1.029371
C	-3.243067	0.189881	0.305259
H	-2.111408	1.014717	1.91709
H	-1.509845	-0.544535	1.37532
H	-3.627678	1.139294	-0.063412
C	-5.542097	-0.256534	1.076374
C	-6.411222	-0.757756	2.205986
H	-7.067271	-1.538326	1.823336
C	-3.068474	-0.682466	-0.950275
N	-3.263223	-2.002221	-0.802765
H	-3.605532	-2.337781	0.081459
O	-2.727267	-0.165417	-2.014188

N	-4.198639	-0.349496	1.262471
H	-3.859168	-0.66994	2.154556
O	-6.019164	0.20346	0.040836
H	-5.842122	-1.150057	3.04552
H	-7.038725	0.062286	2.552904
C	-3.122182	-2.94199	-1.904831
H	-2.116175	-2.900594	-2.319165
H	-3.310164	-3.943787	-1.530743
H	-3.832605	-2.720592	-2.701213
C	2.811084	0.87194	-0.493998
C	3.070153	1.234967	0.934122
C	3.244963	-0.525472	-0.920668
C	3.801439	0.462171	1.750159
H	2.681727	2.183722	1.281545
C	4.103705	-1.275701	0.015646
C	4.33755	-0.801746	1.263564
H	4.937318	-1.379663	1.952094
O	2.922392	-0.98437	-2.0085
O	4.548311	-2.448099	-0.490445
C	5.348572	-3.278265	0.354438
H	4.79223	-3.578863	1.243397
H	5.594558	-4.15341	-0.237646
H	6.264266	-2.764667	0.650537
C	4.109746	0.841625	3.173776
H	3.740489	0.083982	3.868058
H	5.187557	0.919443	3.331414
H	3.656232	1.795707	3.434355
H	1.290593	0.739595	-1.967878
H	3.448343	1.527347	-1.107138

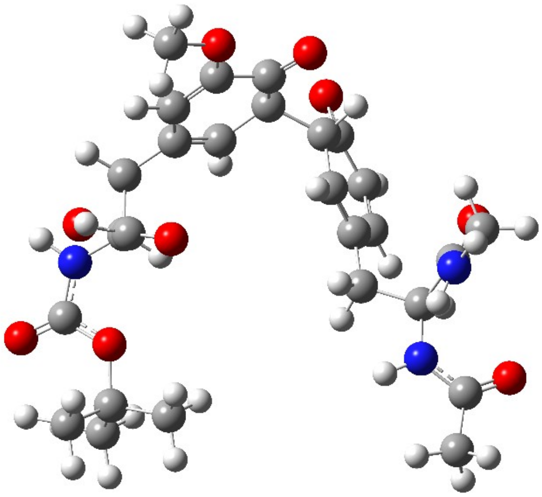

ketone-peptide_plus_2-methoxy-4ethyl-COOH-NHBoc-phenone_b3lyp_6311gplus2d2p_H2O
Zero-point correction= 0.600304 (Hartree/Particle)
Thermal correction to Energy= 0.642454
Thermal correction to Enthalpy= 0.643398

Thermal correction to Gibbs Free Energy= 0.515849
Sum of electronic and zero-point Energies= -1891.293046
Sum of electronic and thermal Energies= -1891.250896
Sum of electronic and thermal Enthalpies= -1891.249951
Sum of electronic and thermal Free Energies= -1891.377501

C	2.243993	0.182505	-1.1644
C	1.68098	1.264622	-0.606429
C	1.517561	2.564068	-1.33128
C	1.833965	2.529875	-2.825821
C	2.490519	1.336047	-3.336134
C	2.663492	0.247439	-2.558003
H	1.361403	1.23705	0.427987
H	3.115726	-0.636138	-2.992022
O	1.562706	3.487254	-3.546605
H	2.779881	1.337533	-4.37772
C	2.413243	-1.114071	-0.413924
C	3.876691	-1.549002	-0.163623
H	1.94038	-1.91821	-0.98302
H	1.892963	-1.0482	0.541966
H	4.410638	-1.614244	-1.110085
C	4.941327	-3.700768	0.400952
C	4.743448	-5.085655	0.971798
H	5.506599	-5.265889	1.727188
C	4.644019	-0.495164	0.653879
N	4.67041	-0.661995	1.9854
H	4.283726	-1.508013	2.368136
O	5.162101	0.464909	0.083346
N	3.866102	-2.87017	0.448277
H	2.991205	-3.237364	0.785136
O	6.014847	-3.339096	-0.076688
H	3.760745	-5.232868	1.413679
H	4.887106	-5.814312	0.174523
C	5.350821	0.265515	2.876724
H	4.952149	1.271737	2.759686
H	5.194056	-0.060978	3.900356
H	6.421118	0.292403	2.672125
C	0.165461	3.268387	-1.047637
C	-1.027596	2.418899	-1.345555
C	0.170991	3.966882	0.307025
C	-2.111826	2.40414	-0.556214
H	-1.007999	1.84284	-2.261794
C	-1.095518	3.966926	1.065288
C	-2.145119	3.215819	0.651156
H	-3.05535	3.206116	1.232875
O	1.170719	4.536658	0.721691
O	-1.048966	4.726951	2.180469
C	-2.209484	4.762189	3.01543

H	-2.444213	3.767167	3.395392
H	-1.959864	5.420653	3.84057
H	-3.067066	5.161329	2.472228
C	-3.329686	1.57704	-0.902474
H	-4.23453	2.099358	-0.585846
H	-3.389773	1.454748	-1.982678
C	-3.345003	0.145557	-0.308514
H	-2.463127	-0.383815	-0.657595
C	-3.23614	0.162979	1.217206
O	-2.197022	0.027835	1.818098
O	-4.415603	0.376452	1.823968
H	-4.266284	0.396994	2.78175
N	-4.532941	-0.535591	-0.790897
H	-5.35373	0.016133	-0.980217
C	-4.774755	-1.877144	-0.691959
O	-5.842164	-2.378097	-1.00687
O	-3.696146	-2.526936	-0.228909
C	-3.664796	-4.005293	-0.084829
C	-2.252989	-4.25135	0.440535
H	-1.509096	-3.892411	-0.269772
H	-2.10133	-3.743234	1.392144
H	-2.098666	-5.318894	0.591412
C	-4.707303	-4.45012	0.937998
H	-5.717338	-4.282558	0.576028
H	-4.580474	-5.514459	1.136094
H	-4.572315	-3.912835	1.876668
C	-3.856297	-4.664867	-1.448316
H	-3.694427	-5.738341	-1.350433
H	-4.857851	-4.499608	-1.834311
H	-3.13082	-4.277372	-2.163589
H	0.135874	4.10791	-1.759503
H	2.281901	3.241897	-0.922094

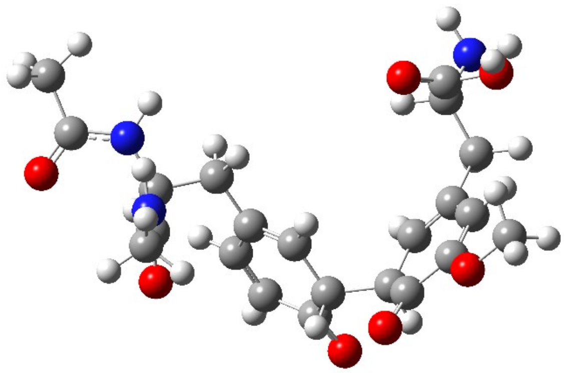

ketone-peptide_plus_2-methoxy-4ethyl-COOH-NH2-phenone_b3lyp_6311gplus2d2p_H2O
Zero-point correction= 0.474907 (Hartree/Particle)
Thermal correction to Energy= 0.508454
Thermal correction to Enthalpy= 0.509398
Thermal correction to Gibbs Free Energy= 0.403371

Sum of electronic and zero-point Energies= -1545.470117
Sum of electronic and thermal Energies= -1545.436571
Sum of electronic and thermal Enthalpies= -1545.435627
Sum of electronic and thermal Free Energies= -1545.541653

C	-1.472884	-0.490673	-1.095842
C	-0.40868	-0.935249	-0.411093
C	0.402388	-2.117547	-0.84116
C	0.08386	-2.664754	-2.231745
C	-1.117075	-2.161873	-2.881431
C	-1.824674	-1.143488	-2.349781
H	-0.12855	-0.463202	0.522391
H	-2.686639	-0.770756	-2.889835
O	0.805053	-3.511614	-2.753684
H	-1.384384	-2.598968	-3.833349
C	-2.289937	0.690864	-0.638036
C	-3.755387	0.372795	-0.256021
H	-2.329059	1.428629	-1.443298
H	-1.795273	1.165962	0.20981
H	-4.256495	-0.119166	-1.087788
C	-5.79714	1.743393	-0.060861
C	-6.359625	3.132579	0.130905
H	-7.057777	3.115875	0.966619
C	-3.826976	-0.635875	0.903882
N	-3.922855	-0.128693	2.142883
H	-4.047929	0.864228	2.24343
O	-3.755674	-1.842913	0.672307
N	-4.444909	1.626251	0.014603
H	-3.897062	2.464284	0.120031
O	-6.518901	0.769651	-0.266743
H	-5.596088	3.883798	0.318969
H	-6.918458	3.408359	-0.762534
C	-3.988465	-0.968781	3.329252
H	-3.12319	-1.627288	3.379549
H	-3.998022	-0.328475	4.206076
H	-4.889532	-1.582327	3.32728
C	1.927951	-1.93307	-0.631832
C	2.485456	-0.730985	-1.323431
C	2.313528	-2.103877	0.832741
C	3.407147	0.062064	-0.756521
H	2.150482	-0.544203	-2.335778
C	3.393469	-1.239001	1.346051
C	3.879787	-0.223315	0.59087
H	4.653173	0.415902	0.990662
O	1.776967	-2.950766	1.533802
O	3.771327	-1.549003	2.605475
C	4.780963	-0.742533	3.217952
H	4.454187	0.295155	3.296062

H	4.928598	-1.156505	4.209735
H	5.713901	-0.793353	2.655032
C	3.981764	1.255558	-1.487641
H	5.036122	1.378961	-1.233018
H	3.928291	1.086468	-2.56171
C	3.247183	2.588924	-1.230214
H	2.2036	2.471803	-1.51518
C	3.2252	2.972221	0.247945
O	2.224907	3.085028	0.919348
O	4.459045	3.205687	0.738912
H	4.374199	3.473978	1.666342
N	3.831633	3.635934	-2.073379
H	4.781428	3.840062	-1.783289
H	3.304701	4.497774	-1.990947
H	0.120552	-2.942435	-0.169398
H	2.383245	-2.811832	-1.113895

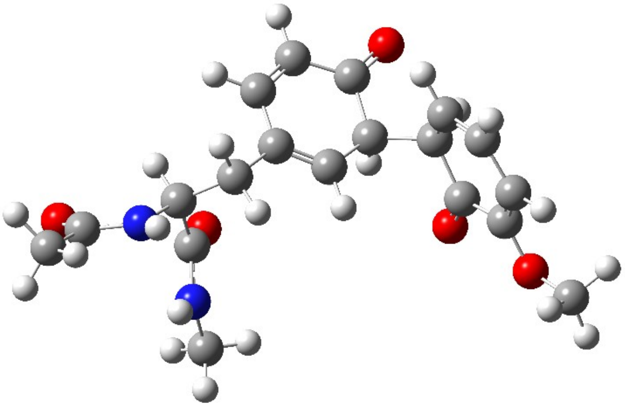

ketone-peptide_plus_2-methoxyphenone_b3lyp_6311gplus2d2p_H2O
Zero-point correction= 0.386041 (Hartree/Particle)
Thermal correction to Energy= 0.412608
Thermal correction to Enthalpy= 0.413552
Thermal correction to Gibbs Free Energy= 0.324157
Sum of electronic and zero-point Energies= -1222.892863
Sum of electronic and thermal Energies= -1222.866296
Sum of electronic and thermal Enthalpies= -1222.865352
Sum of electronic and thermal Free Energies= -1222.954746

C	-0.716264	1.143175	0.406231
C	0.46218	0.579599	0.101086
C	1.553743	1.31123	-0.615763
C	1.343776	2.816098	-0.775123
C	0.017759	3.33325	-0.472656
C	-0.926544	2.548901	0.087295
H	0.640469	-0.463713	0.330616
H	-1.884877	2.98696	0.338717
O	2.254913	3.53981	-1.169659
H	-0.158182	4.383481	-0.658918

C	-1.814223	0.385789	1.109179
C	-3.099748	0.160736	0.277543
H	-2.112091	0.939776	2.002787
H	-1.430928	-0.579268	1.442005
H	-3.483755	1.115742	-0.077063
C	-5.435279	-0.374453	0.862186
C	-6.367531	-0.942491	1.906755
H	-6.978949	-1.717422	1.446677
C	-2.809258	-0.649047	-0.998279
N	-2.974352	-1.978857	-0.921697
H	-3.373875	-2.361845	-0.081782
O	-2.407235	-0.075869	-2.010899
N	-4.105892	-0.4456	1.138107
H	-3.821778	-0.793374	2.039259
O	-5.851117	0.11881	-0.184283
H	-5.848238	-1.360824	2.765785
H	-7.034926	-0.150757	2.244978
C	-2.727737	-2.8643	-2.049771
H	-1.702659	-2.760701	-2.401303
H	-2.889583	-3.888297	-1.727083
H	-3.400764	-2.641656	-2.877822
C	2.974417	0.978861	-0.093405
C	3.150181	1.214063	1.372952
C	3.461654	-0.369693	-0.619012
C	3.85394	0.366338	2.132728
H	2.729782	2.118347	1.791769
C	4.282866	-1.196084	0.288321
C	4.44171	-0.839546	1.587738
H	5.009478	-1.467377	2.257943
O	3.208064	-0.727394	-1.76146
O	4.771528	-2.309644	-0.301231
C	5.539823	-3.207231	0.503701
H	4.941601	-3.598639	1.327625
H	5.832625	-4.018323	-0.154552
H	6.42897	-2.712911	0.897326
H	4.004995	0.566583	3.184345
H	3.634347	1.697418	-0.603685
H	1.538031	0.941114	-1.652487

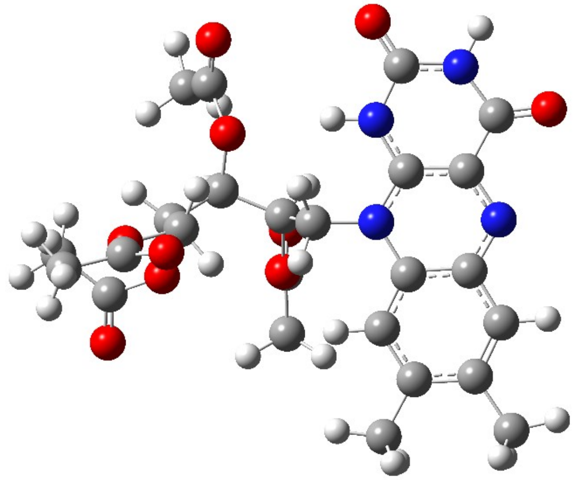

RTA-H_1ered_rad_neut_b3lyp_6311gplus2d2p_H2O_4_opttight
Zero-point correction= 0.531717 (Hartree/Particle)
Thermal correction to Energy= 0.571402
Thermal correction to Enthalpy= 0.572347
Thermal correction to Gibbs Free Energy= 0.452629
Sum of electronic and zero-point Energies= -1941.566106
Sum of electronic and thermal Energies= -1941.526421
Sum of electronic and thermal Enthalpies= -1941.525477
Sum of electronic and thermal Free Energies= -1941.645194

C	4.508285	2.586692	0.490005
C	3.814615	1.303383	0.450754
C	2.421917	1.298016	0.411534
C	2.312067	3.735064	0.44756
C	2.470563	-1.093315	0.368508
C	3.886732	-1.00556	0.427919
C	4.618991	-2.210226	0.443484
H	5.696175	-2.125706	0.494554
C	4.017594	-3.454373	0.397723
C	2.608178	-3.524144	0.323336
C	1.864487	-2.344057	0.306465
H	4.139322	4.611905	0.514532
H	0.7943	-2.430726	0.220105
N	1.729707	0.119392	0.381281
N	4.563545	0.173232	0.46961
N	1.723554	2.482718	0.408009
N	3.679587	3.712935	0.484969
C	1.901238	-4.851403	0.254663
H	2.215534	-5.425666	-0.619553
H	2.122915	-5.466279	1.129577
H	0.822576	-4.720229	0.200943
C	4.853912	-4.706955	0.421448
H	4.602937	-5.336518	1.277431
H	4.691125	-5.311749	-0.472791
H	5.913586	-4.467135	0.478224

O	1.649825	4.760129	0.446931
O	5.72295	2.736088	0.531028
C	0.264243	0.108826	0.465993
C	-0.433392	0.258233	-0.892391
O	-0.429954	-1.027352	-1.530477
C	-0.366345	-1.055548	-2.888273
O	-0.344127	-0.053786	-3.562937
H	0.136374	0.944448	-1.516732
H	-0.048022	0.905679	1.134695
H	-0.030543	-0.815057	0.947167
C	-0.333545	-2.464617	-3.396287
H	0.516194	-2.993396	-2.96658
H	-1.237805	-2.986571	-3.084641
H	-0.262715	-2.461467	-4.478561
C	-1.861252	0.851297	-0.851201
H	-2.18688	0.906816	-1.885178
O	-1.710009	2.169673	-0.289276
C	-2.132092	3.317518	-0.916422
O	-2.039504	4.338217	-0.286753
C	-2.648024	3.235123	-2.325023
H	-1.900785	2.80672	-2.992293
H	-3.545802	2.621543	-2.386047
H	-2.886295	4.241143	-2.651939
C	-3.001932	0.151137	-0.076447
H	-3.873629	0.787855	-0.204433
C	-2.785696	-0.040682	1.413971
H	-2.420253	0.875684	1.872368
H	-2.095432	-0.853989	1.620183
O	-3.223604	-1.130832	-0.688462
O	-4.073493	-0.372137	1.968471
C	-4.108231	-0.629115	3.295137
C	-4.432652	-1.553813	-1.148013
C	-5.619244	-0.631751	-1.079559
H	-6.475712	-1.156848	-1.4877
H	-5.827587	-0.340747	-0.051517
H	-5.448207	0.274077	-1.660407
C	-5.495893	-0.976369	3.748783
H	-5.837079	-1.874012	3.233763
H	-5.499284	-1.143396	4.820498
H	-6.183188	-0.170369	3.495063
O	-4.481318	-2.672745	-1.599553
O	-3.124526	-0.577663	3.996574
H	0.716353	2.509161	0.365809

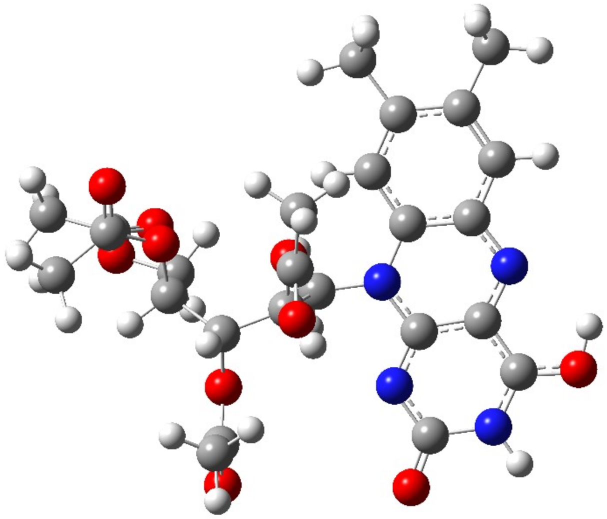

RTA-H_1ered_rad-OH_neut_b3lyp_6311gplus2d2p_H2O_6_readwrite_freqonly

Zero-point correction= 0.531465 (Hartree/Particle)
Thermal correction to Energy= 0.571136
Thermal correction to Enthalpy= 0.572080
Thermal correction to Gibbs Free Energy= 0.451744
Sum of electronic and zero-point Energies= -1941.559531
Sum of electronic and thermal Energies= -1941.519860
Sum of electronic and thermal Enthalpies= -1941.518916
Sum of electronic and thermal Free Energies= -1941.639252

C	4.70701	-2.021028	-0.575385
C	3.906144	-0.885885	-0.499693
C	2.499642	-1.123313	-0.546428
C	2.745322	-3.427434	-0.737868
C	2.245717	1.265247	-0.350507
C	3.665164	1.393213	-0.331063
C	4.21806	2.686752	-0.229119
H	5.297459	2.757271	-0.221179
C	3.442954	3.825643	-0.141782
C	2.034283	3.686943	-0.146392
C	1.468803	2.417383	-0.245537
H	4.726956	-4.058439	-0.742973
H	0.394421	2.347058	-0.215927
N	1.68252	-0.026034	-0.476153
N	4.50062	0.324902	-0.403575
N	1.954484	-2.325391	-0.657721
N	4.143069	-3.235965	-0.685633
C	1.140989	4.893371	-0.037872
H	1.330382	5.44729	0.884062
H	1.311948	5.589399	-0.861915
H	0.091156	4.608501	-0.050461
C	4.087217	5.183079	-0.036954
H	3.787714	5.831078	-0.863005

H	3.796632	5.692164	0.884135
H	5.172065	5.101007	-0.048887
O	2.323743	-4.577193	-0.849829
O	6.030827	-1.95805	-0.548184
C	0.233585	-0.230966	-0.613953
C	-0.429013	-0.628934	0.703844
O	-0.382112	0.504251	1.591172
C	-0.248672	0.259827	2.917532
O	-0.206085	-0.856512	3.381162
H	0.154948	-1.433505	1.144717
H	0.08566	-1.037678	-1.32443
H	-0.193614	0.672986	-1.026953
C	-0.168241	1.537991	3.697303
H	0.671608	2.134762	3.343669
H	-1.075022	2.121052	3.539676
H	-0.049765	1.316777	4.752535
C	-1.872844	-1.174988	0.612085
H	-2.161457	-1.4217	1.629248
O	-1.832262	-2.356586	-0.208047
C	-1.892901	-3.624198	0.293715
O	-1.850471	-4.527428	-0.504059
C	-2.015484	-3.820327	1.779526
H	-1.225736	-3.303501	2.323013
H	-2.973071	-3.44066	2.137342
H	-1.960437	-4.883864	1.983565
C	-3.032641	-0.322622	0.050351
H	-3.901915	-0.973214	0.087245
C	-2.884004	0.166754	-1.377502
H	-2.521007	-0.629532	-2.023092
H	-2.218279	1.02257	-1.441499
O	-3.218809	0.815217	0.911345
O	-4.201442	0.56882	-1.80452
C	-4.299046	1.080512	-3.050595
C	-4.374685	1.078328	1.575455
C	-5.531538	0.122544	1.463838
H	-6.349044	0.514905	2.05846
H	-5.85469	0.018875	0.429129
H	-5.263158	-0.866004	1.835772
C	-5.712194	1.469799	-3.373634
H	-6.054378	2.230171	-2.67223
H	-5.763543	1.853664	-4.386818
H	-6.368636	0.606864	-3.268984
O	-4.406756	2.088909	2.236895
O	-3.346462	1.19702	-3.787153
H	6.264843	-1.014623	-0.469092

WB97XD

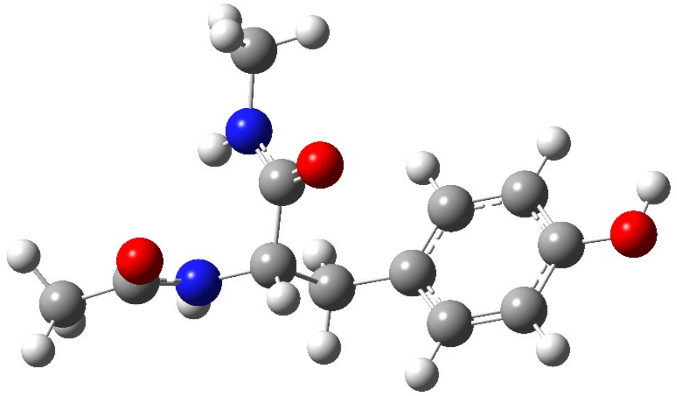

phenol-peptide_wb97xd_6311gplus2d2p_H2O
Zero-point correction= 0.273741 (Hartree/Particle)
Thermal correction to Energy= 0.291537
Thermal correction to Enthalpy= 0.292481
Thermal correction to Gibbs Free Energy= 0.225311
Sum of electronic and zero-point Energies= -801.871738
Sum of electronic and thermal Energies= -801.853942
Sum of electronic and thermal Enthalpies= -801.852998
Sum of electronic and thermal Free Energies= -801.920168

C	1.358383	-0.709904	-0.656394
C	2.036752	0.428913	-1.073923
C	3.339631	0.682328	-0.6682
C	3.984746	-0.215687	0.172997
C	3.324193	-1.363359	0.597981
C	2.025191	-1.600534	0.182031
H	1.542162	1.138485	-1.725646
H	1.520611	-2.498347	0.517562
O	5.261248	-0.026511	0.608041
H	3.851374	1.574858	-1.005851
H	3.835778	-2.060398	1.24739
C	-0.065952	-0.963193	-1.072022
C	-1.102758	-0.580572	-0.000388
H	-0.205692	-2.022642	-1.293849
H	-0.28804	-0.409283	-1.986375
H	-0.924293	-1.16101	0.904496
C	-3.523095	-0.850791	0.272981
C	-4.822752	-1.257068	-0.371883
H	-5.511067	-0.414461	-0.324448
C	-0.94714	0.886437	0.418143
N	-1.533281	1.789397	-0.379206
H	-2.13397	1.459266	-1.113559
O	-0.274476	1.19347	1.39523
N	-2.423727	-0.878386	-0.515045

H	-2.507469	-1.226487	-1.454244
O	-3.459495	-0.499604	1.444876
H	5.610037	0.793817	0.253631
H	-4.710011	-1.567296	-1.408073
H	-5.254916	-2.07457	0.202735
C	-1.44985	3.211811	-0.125073
H	-0.409449	3.532213	-0.091736
H	-1.956244	3.739342	-0.928066
H	-1.922063	3.470302	0.823099

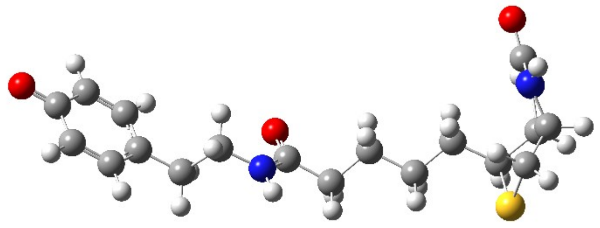

biotin-tyramide_rad_wb97xd_6311gplus2d2p_h2o_optfreqtight_geom2
Zero-point correction= 0.411593 (Hartree/Particle)
Thermal correction to Energy= 0.435802
Thermal correction to Enthalpy= 0.436746
Thermal correction to Gibbs Free Energy= 0.350463
Sum of electronic and zero-point Energies= -1488.130167
Sum of electronic and thermal Energies= -1488.105958
Sum of electronic and thermal Enthalpies= -1488.105014
Sum of electronic and thermal Free Energies= -1488.191297

C	-7.590772	1.343641	-0.587541
C	-6.418644	0.689799	-0.833057
C	-8.452892	0.936491	0.50221
C	-6.01038	-0.402025	-0.037378
H	-5.784699	1.003898	-1.652549
C	-8.010041	-0.18442	1.304266
C	-6.831534	-0.816881	1.032005
H	-8.645853	-0.499057	2.120414
H	-6.514383	-1.654467	1.640124
O	-9.52854	1.523701	0.738306
C	-4.695752	-1.070586	-0.290986
C	-3.560112	-0.354678	0.462589
H	-4.464367	-1.053778	-1.356067
H	-4.73244	-2.111058	0.032351
H	-3.514826	0.688124	0.152222
H	-3.750562	-0.378615	1.534218
C	-1.524095	-0.672072	-0.866836
C	-0.194231	-1.384684	-0.969425
C	0.968202	-0.426324	-0.699002
H	-0.115127	-1.782014	-1.982306
H	-0.148909	-2.227953	-0.278801
C	2.324271	-1.107845	-0.84072

H	0.903901	0.416739	-1.389948
H	0.871024	-0.016758	0.310133
C	3.479803	-0.154376	-0.563489
H	2.378009	-1.95808	-0.156942
H	2.423322	-1.512634	-1.852549
H	3.409557	0.688458	-1.255596
H	3.381238	0.26505	0.440879
N	-2.270889	-0.960978	0.218505
H	-1.951113	-1.676412	0.846396
O	-1.883877	0.149148	-1.706033
C	5.697079	-0.857713	1.744358
C	6.354627	0.300082	0.981817
C	4.855697	-0.793331	-0.731619
C	6.051266	0.166676	-0.535916
C	5.601902	2.354309	0.155808
S	5.215964	-2.098918	0.496963
H	6.909586	-0.2505	-1.056889
H	7.431571	0.296232	1.150113
N	5.841283	1.545588	-0.919371
H	5.498038	1.812876	-1.82443
N	5.792097	1.605347	1.289598
H	6.047212	2.089137	2.132731
O	5.276373	3.531958	0.116177
H	4.919953	-1.265428	-1.711764
H	6.372284	-1.327509	2.453298
H	4.816905	-0.506616	2.277876
H	-7.911197	2.178516	-1.195538

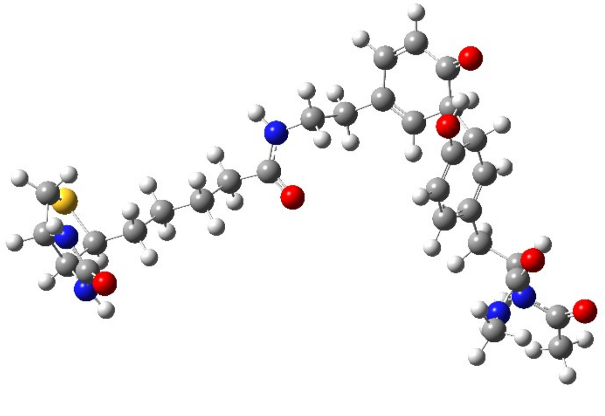

peptide-OH-rad_plus_biotin-tyramide_ketone_wb97xd_6311gplus2d2p_h2o_mfgeom_unfreeze1to54_optfreqtight_2

Zero-point correction= 0.687492 (Hartree/Particle)
Thermal correction to Energy= 0.730392
Thermal correction to Enthalpy= 0.731336
Thermal correction to Gibbs Free Energy= 0.600042
Sum of electronic and zero-point Energies= -2289.972983

Sum of electronic and thermal Energies= -2289.930083
Sum of electronic and thermal Enthalpies= -2289.929139
Sum of electronic and thermal Free Energies= -2290.060433

C	4.653706	-0.191136	-0.030406
C	3.805691	-0.469265	1.072413
C	3.571001	0.508877	2.056195
C	4.092142	1.759767	1.931977
C	4.83226	2.205698	0.705324
C	5.169025	1.054126	-0.192971
H	3.383774	-1.457978	1.188891
H	5.818457	1.271066	-1.032942
O	3.924473	2.659303	2.927756
H	2.996958	0.267156	2.940999
C	5.00318	-1.299357	-0.99134
C	6.302335	-2.044015	-0.63638
H	5.123552	-0.89704	-1.997781
H	4.183537	-2.020487	-1.025051
H	7.142284	-1.352091	-0.691007
C	7.648046	-3.811498	-1.673169
C	7.73992	-4.839718	-2.770709
H	7.978027	-5.803688	-2.324469
C	6.282989	-2.544794	0.812836
N	5.625479	-3.691468	1.029841
H	5.291629	-4.210271	0.237175
O	6.798717	-1.8897	1.710732
N	6.490962	-3.115117	-1.594149
H	5.787432	-3.262177	-2.296913
O	8.565904	-3.616457	-0.885649
H	6.82559	-4.929953	-3.352574
H	8.559644	-4.5663	-3.433641
C	5.489543	-4.261384	2.353039
H	4.990607	-3.559913	3.020852
H	4.894617	-5.167237	2.283071
H	6.463711	-4.507767	2.775933
C	4.033205	3.28694	-0.096215
C	2.763013	2.78504	-0.698477
C	3.909123	4.625569	0.603933
C	1.712456	3.579229	-0.928686
H	2.743464	1.743963	-0.99675
C	2.831991	5.492917	0.152547
C	1.796081	4.979615	-0.534141
H	2.840905	6.520393	0.488164
H	0.963303	5.625434	-0.788584
O	4.687073	4.963816	1.490749
C	0.436992	3.079036	-1.547115
C	-0.669896	2.913939	-0.498939
H	0.609752	2.116859	-2.027828

H	0.09642	3.775818	-2.316254
H	-0.344877	2.205383	0.261683
H	-0.874001	3.863062	-0.004309
C	-2.154138	1.126133	-1.290729
C	-3.497605	0.793774	-1.901341
C	-4.426751	0.136935	-0.877825
H	-3.316977	0.105202	-2.728066
H	-3.970886	1.686605	-2.312646
C	-5.774267	-0.240326	-1.482227
H	-3.940383	-0.752605	-0.472392
H	-4.583287	0.822019	-0.040146
C	-6.70267	-0.892753	-0.465581
H	-6.250852	0.652512	-1.893454
H	-5.617068	-0.925825	-2.320603
H	-6.215876	-1.789412	-0.073851
H	-6.847126	-0.225116	0.387577
N	-1.909333	2.433421	-1.071559
H	-2.584789	3.106928	-1.385263
O	-1.351	0.248818	-0.983047
C	-9.653834	0.3867	0.152576
C	-9.739947	-0.996981	0.809982
C	-8.054367	-1.300008	-1.046266
C	-8.992591	-2.039972	-0.065985
C	-8.307029	-2.260822	2.158413
S	-9.089682	0.116744	-1.561006
H	-9.702913	-2.620312	-0.649738
H	-10.782759	-1.289577	0.932684
N	-8.356872	-2.868089	0.935297
H	-7.683382	-3.577254	0.707593
N	-9.027343	-1.096085	2.073722
H	-9.428863	-0.731283	2.919797
O	-7.725707	-2.678197	3.149496
H	-7.899021	-1.90901	-1.936655
H	-10.612908	0.895089	0.122152
H	-8.94123	1.01317	0.683954
H	5.755715	2.703933	1.020161
H	4.678622	3.546163	-0.953323
H	4.350544	3.493761	2.682894

References

1. Szklarczyk, D., Gable, A.L., Lyon, D., Junge, A., Wyder, S., Huerta-Cepas, J., Simonovic, M., Doncheva, N.T., Morris, J.H., Bork, P. and Jensen, L.J., 2019. STRING v11: protein–protein association networks with increased coverage, supporting functional discovery in genome-wide experimental datasets. *Nucleic acids research*, **2019**, *47*, D607-D613.
2. Rehm, D. and Weller, A., Kinetics of fluorescence quenching by electron and H-atom transfer. *Israel Journal of Chemistry*, **1970**, *8*, 259-271
3. Romero, N.A. and Nicewicz, D.A., Organic photoredox catalysis. *Chemical reviews*, **2016**, *116*, 10075-10166
4. Close, D.M. and Wardman, P., Calculation of standard reduction potentials of amino acid radicals and the effects of water and incorporation into peptides. *The Journal of Physical Chemistry A*, **2018**, *122*, 439-445.
5. Lind, J., Shen, X., Eriksen, T.E. and Merenyi, G., 1990. The one-electron reduction potential of 4-substituted phenoxyl radicals in water. *Journal of the American Chemical Society*, **1990**, *112*, 479-482
6. Sjöback, R., Nygren, J. and Kubista, M., Absorption and fluorescence properties of fluorescein. *Spectrochimica Acta Part A: Molecular and Biomolecular Spectroscopy*, **1995**, *51*, L7-L21.
7. Perkin Elmer, https://www.perkinelmer.com/lab-solutions/resources/docs/APP_Determination_of_Relative_FluorescenceQuantum_Yields_using_FL6500_Fluorescence_Spect.pdf, accessed 2022-07-28
8. Pitre, S.P., McTiernan, C.D., Vine, W., DiPucchio, R., Grenier, M. and Scaiano, J.C., Visible-light actinometry and intermittent illumination as convenient tools to study Ru (bpy) 3Cl2 mediated photoredox transformations. *Scientific reports*, **2015**, *5*, 1-10.
9. Wilkinson, F., Helman, W.P. and Ross, A.B., Rate constants for the decay and reactions of the lowest electronically excited singlet state of molecular oxygen in solution. An expanded and revised compilation. *Journal of Physical and Chemical Reference Data*, **1995**, *24*, 663-677
10. Burton, G.W. and Ingold, K.U., Vitamin E: application of the principles of physical organic chemistry to the exploration of its structure and function. *Accounts of chemical research*, **1986**, *19*, 194-201.
11. Oslund, R. C.; Reyes-Robles, T.; White, C. H.; Tomlinson, J. H.; Crotty, K. A.; Bowman, E. P.; Chang, D.; Peterson, V. M.; Li, L.; Frutos, S.; et al. Detection of Cell-Cell Interactions via Photocatalytic Cell Tagging. *Nat. Chem. Bio.*, **2022**, https://doi.org/10.1038/s41589-022-01044-0
12. Geri, J. B.; Oakley, J. V.; Reyes-Robles, T.; Wang, T.; McCarver, S. J.; White, C. H.; Rodriguez-Rivera, F. P.; Parker, D. L.; Hett, E. C.; Fadeyi, O. O.; Oslund, R. C.; MacMillan, D. W. Microenvironment mapping via Dexter energy transfer on immune cells. *Science,* **2020**, *367*, 1091−1097.
13. Gaussian 09, Revision C.01, M. J. Frisch, G. W. Trucks, H. B. Schlegel, G. E. Scuseria, M. A. Robb, J. R. Cheeseman, G. Scalmani, V. Barone, G. A. Petersson, H. Nakatsuji, X. Li, M. Caricato, A. Marenich, J. Bloino, B. G. Janesko, R. Gomperts, B. Mennucci, H. P. Hratchian, J. V. Ortiz, A. F. Izmaylov, J. L. Sonnenberg, D. Williams-Young, F. Ding, F. Lipparini, F. Egidi, J. Goings, B. Peng, A. Petrone, T. Henderson, D. Ranasinghe, V. G. Zakrzewski, J. Gao, N. Rega, G. Zheng, W. Liang, M. Hada, M. Ehara, K. Toyota, R. Fukuda, J. Hasegawa, M. Ishida, T. Nakajima, Y. Honda, O. Kitao, H. Nakai, T. Vreven, K. Throssell, J. A. Montgomery, Jr., J. E. Peralta, F. Ogliaro, M. Bearpark, J. J. Heyd, E. Brothers, K. N. Kudin, V. N. Staroverov, T. Keith, R. Kobayashi, J. Normand, K. Raghavachari, A. Rendell, J. C. Burant, S. S. Iyengar, J. Tomasi, M. Cossi, J. M. Millam, M. Klene, C. Adamo, R. Cammi, J. W. Ochterski, R. L. Martin, K. Morokuma, O. Farkas, J. B. Foresman, and D. J. Fox, Gaussian, Inc., Wallingford CT, 2016.

14. Gaussian 16, Revision C.01, M. J. Frisch, G. W. Trucks, H. B. Schlegel, G. E. Scuseria, M. A. Robb, J. R. Cheeseman, G. Scalmani, V. Barone, G. A. Petersson, H. Nakatsuji, X. Li, M. Caricato, A. V. Marenich, J. Bloino, B. G. Janesko, R. Gomperts, B. Mennucci, H. P. Hratchian, J. V. Ortiz, A. F. Izmaylov, J. L. Sonnenberg, D. Williams-Young, F. Ding, F. Lipparini, F. Egidi, J. Goings, B. Peng, A. Petrone, T. Henderson, D. Ranasinghe, V. G. Zakrzewski, J. Gao, N. Rega, G. Zheng, W. Liang, M. Hada, M. Ehara, K. Toyota, R. Fukuda, J. Hasegawa, M. Ishida, T. Nakajima, Y. Honda, O. Kitao, H. Nakai, T. Vreven, K. Throssell, J. A. Montgomery, Jr., J. E. Peralta, F. Ogliaro, M. J. Bearpark, J. J. Heyd, E. N. Brothers, K. N. Kudin, V. N. Staroverov, T. A. Keith, R. Kobayashi, J. Normand, K. Raghavachari, A. P. Rendell, J. C. Burant, S. S. Iyengar, J. Tomasi, M. Cossi, J. M. Millam, M. Klene, C. Adamo, R. Cammi, J. W. Ochterski, R. L. Martin, K. Morokuma, O. Farkas, J. B. Foresman, and D. J. Fox, Gaussian, Inc., Wallingford CT, 2016
15. Becke, A.D., Density functional thermochemistry. I. The effect of the exchange only gradient correction. *The Journal of chemical physics*, **1992**, *96*, 2155-2160.
16. Lee, C., Yang, W. and Parr, R.G., Development of the Colle-Salvetti correlation-energy formula into a functional of the electron density. *Physical review B*, **1988**, *37*, 785.
17. Barone, V. and Cossi, M., Quantum calculation of molecular energies and energy gradients in solution by a conductor solvent model. *The Journal of Physical Chemistry A*, **1998**, *102*, 1995-2001.
18. Johnson, E. R.; Becke, A. D. *J. Chem. Phys.* **2006**, 124, 174104.
19. Grimme S, Ehrlich S, Goerigk L. Effect of the damping function in dispersion corrected density functional theory, *Journal of computational chemistry*, **2011** 32(7), 1456-65.
20. Sato, S. and Nakamura, H., Ligand Directed Selective Protein Modification Based on Local Single Electron Transfer Catalysis. *Angewandte Chemie*, **2013**, *125*, 8843-8846.
21. Tsushima, M., Sato, S. and Nakamura, H., Selective purification and chemical labeling of a target protein on ruthenium photocatalyst-functionalized affinity beads. *Chemical Communications*, **2017**, *53*, 4838-4841.
22. Li, B.X., Kim, D.K., Bloom, S., Huang, R.Y.C., Qiao, J.X., Ewing, W.R., Oblinsky, D.G., Scholes, G.D. and MacMillan, D.W., Site-selective tyrosine bioconjugation via photoredox catalysis for native-to-bioorthogonal protein transformation. *Nature chemistry*, **2021**, *13*, 902-908
